# Acute Cervical Carotid Management During Thrombectomy for Tandem Occlusion: A Systematic Review and Network Meta-Analysis

**DOI:** 10.3390/brainsci16101128

**Published:** 2026-10-19

**Authors:** Qin Yang, Xuzi Li, Muke Zhou

**Affiliations:** 1 Department of Neurology, West China Hospital, Sichuan University, No. 37 Guoxue Alley, Wuhou District, Chengdu 610041, China; qinyang92647@163.com (Q.Y.); lixuzi1999@163.com (X.L.); 2 Center of Cerebrovascular Disease, Department of Neurology, West China Hospital, Sichuan University, No. 37 Guoxue Alley, Wuhou District, Chengdu 610041, China

**Keywords:** acute ischaemic stroke, tandem occlusion, mechanical thrombectomy, carotid stenting, balloon angioplasty, network meta-analysis

## Abstract

**Highlights:**

**What are the main findings?**

- Emergent carotid stenting was associated with greater functional independence than balloon angioplasty alone during thrombectomy for tandem occlusion.
- No difference in symptomatic intracranial hemorrhage was detected, although the confidence interval included clinically important differences.

**What are the implications of the main findings?**

- Balloon angioplasty and no acute cervical revascularization should remain distinct comparators in evidence synthesis.
- The findings identify a clinically relevant comparison for randomized trials that specify accompanying antiplatelet treatment.

**Abstract:**

Background/Objectives: The optimal cervical carotid strategy during thrombectomy for anterior-circulation tandem occlusion remains uncertain. We compared emergent carotid stenting, balloon angioplasty alone, and no targeted acute cervical revascularization. Methods: We searched PubMed, Ovid Embase, Cochrane CENTRAL, Web of Science, and Scopus for comparative studies published from January 2015 to 25 August 2026. A frequentist random-effects network meta-analysis estimated odds ratios (ORs) and 95% confidence intervals (CIs), with outcome-specific cohort-overlap auditing. The event-count network was exploratory; adjusted evidence was analyzed separately. Risk of bias was assessed using RoB 2 or ROBINS-I-aligned domains; confidence was assessed with CINeMA. PROSPERO registration: CRD420261494924. Results: The review included 60 reports representing 49 cohort or report families. Fifteen cohorts contributed 1556 patients for functional independence and 15 contributed 1676 for symptomatic intracranial hemorrhage (sICH). Stenting was associated with greater 90-day functional independence than angioplasty alone (OR, 1.73; 95% CI, 1.22–2.45), without a detected difference in sICH (OR, 1.01; 95% CI, 0.60–1.67). All leave-one-cohort-out functional estimates remained above 1, with 95% CIs excluding 1. Evidence was predominantly nonrandomized. Conclusions: Emergent carotid stenting was associated with greater 90-day functional independence than balloon angioplasty alone, with no detected difference in sICH. Serious or critical risk of bias in all studies in the strict network limits causal interpretation. Imprecision and heterogeneous antiplatelet protocols preclude establishing noninferiority or excluding increased hemorrhagic risk.

## 1. Introduction

Mechanical thrombectomy improves functional outcomes in eligible patients with anterior-circulation large-vessel occlusion and is central to contemporary stroke care [1,2,3]. Tandem occlusion adds a severe stenosis or occlusion of the cervical internal carotid artery to the ipsilateral intracranial occlusion. The cervical lesion can impede access to the intracranial thrombus, restrict inflow after recanalization, and remain a source of early re-embolization [4].

Three acute cervical strategies are commonly used. Emergent carotid stenting provides durable luminal support but usually requires periprocedural antiplatelet treatment. Balloon angioplasty can establish access without leaving an implant, although recoil and reocclusion may compromise the result. A third approach avoids targeted acute cervical revascularization and leaves definitive treatment for a later stage. Surveys of stroke specialists show substantial variation in the choice and sequence of these strategies and in their accompanying antiplatelet regimens [5].

Earlier meta-analyses generally compared emergent stenting with a broad nonstenting group or made a conventional pairwise comparison with balloon angioplasty [6,7,8,9,10]. Pooling no acute treatment and balloon angioplasty combines two clinically different decisions. It also obscures the indirect comparison needed to distinguish whether any association with outcome is specific to stenting or reflects cervical intervention more broadly. Since those reviews, multicenter cohorts, trial subgroups, and individual-participant-data analyses have expanded the adjusted evidence base [11,12,13,14,15,16].

We treated no targeted acute cervical revascularization, balloon angioplasty alone, and emergent carotid stenting as separate nodes. We also audited overlapping registries by outcome and time point. The co-primary outcomes were functional independence at approximately 90 days and symptomatic intracranial hemorrhage (sICH).

## 2. Materials and Methods

### 2.1. Protocol and Reporting

This report was prepared with reference to PRISMA 2020 and the PRISMA extension for network meta-analyses. Search reporting was guided by PRISMA-S, and reporting of observational evidence was informed by MOOSE [17,18,19,20]. The review was registered in PROSPERO (CRD420261494924). The protocol and an amendment summary are provided in Supplementary File S1.

The protocol planned a primary network of adjusted estimates targeting the total effect of treatment across nodes A, B, and C, with an event-count network as a secondary analysis. After data extraction and the cohort-overlap audit, no connected adjusted network across all three nodes was available. On 2 September 2026, the analysis plan was amended before finalization of the analyses. The strict A/B/C event-count network was retained as an exploratory synthesis. Adjusted comparisons of emergent stenting with a source-defined mixed nonstenting node D were reported separately and were not decomposed into A and B. The propensity-score-matched estimate from Farooqui et al. was treated as sensitivity evidence because the matching set included periprocedural antiplatelet treatment, which may occur after treatment allocation.

### 2.2. Eligibility Criteria and Treatment Nodes

We included randomized, quasi-randomized, prospective comparative, and retrospective comparative studies of adults aged 18 years or older who underwent intracranial mechanical thrombectomy for acute anterior-circulation tandem occlusion. Eligible cervical lesions were at least 50% stenosis or occlusion of the cervical internal carotid artery. Eligible intracranial lesions involved the ipsilateral internal carotid artery terminus, M1 segment, proximal M2 segment, or another explicitly defined anterior-circulation large-vessel occlusion. Atherosclerotic and dissection-related lesions were eligible.

We excluded isolated cervical or intracranial lesions, posterior-circulation stroke, chronic carotid occlusion, pseudo-occlusion without a true cervical lesion, pediatric studies, and reports from which the target population, treatment node, or outcome could not be separated. No database language filter was applied. Reports published in Chinese were excluded during screening, whereas English-language studies conducted in China remained eligible. The publication window began on 1 January 2015 to reflect contemporary thrombectomy devices and workflows. We used published or otherwise verifiable aggregate data and did not impute unavailable node-specific results from author correspondence.

Node A comprised no therapeutic cervical angioplasty or permanent stent during the thrombectomy procedure, and later endarterectomy, stenting, or medical treatment was permitted. Node B comprised therapeutic cervical balloon angioplasty during the procedure without a permanent acute stent. Node C comprised placement of a permanent cervical carotid stent during the procedure, with or without angioplasty. Treatment order, stent type, and antithrombotic regimen did not define separate nodes. A source group that combined untreated patients with patients who underwent angioplasty was retained intact as mixed nonstenting node D and entered only expanded or adjusted sensitivity analyses.

### 2.3. Information Sources and Search Strategy

Qin Yang searched PubMed, Ovid Embase, Cochrane CENTRAL, Web of Science Core Collection, and Scopus on 25 August 2026, for records published from 1 January 2015 to that date. Controlled vocabulary and free-text terms covered ischemic stroke, tandem lesions, mechanical thrombectomy, and cervical carotid management. We also checked reference lists of included reports and relevant reviews and performed forward-citation searches in Web of Science and Scopus. Candidates found by citation chasing could be matched to the database exports and were not counted twice. The complete one-time strategies for all five databases are provided in the Supplementary Materials.

### 2.4. Study Selection

After automated and manual deduplication, two reviewers independently screened titles and abstracts and then assessed full texts. Disagreements were resolved by MuKe Zhou. Reasons for exclusion at full-text were assigned from a prespecified hierarchy, and the selection process was recorded in a PRISMA 2020 flow diagram.

### 2.5. Data Extraction and Cohort-Overlap Audit

Two reviewers independently extracted all prespecified variables and separately verified the event counts and denominators for both co-primary outcomes. MuKe Zhou adjudicated disagreements. Extracted variables included study design, centers, recruitment period, tandem-occlusion definition, age, sex, prestroke modified Rankin Scale (mRS), National Institutes of Health Stroke Scale score, Alberta Stroke Program Early CT Score or infarct-core volume, collateral status, intracranial occlusion site, cervical etiology and severity, intravenous thrombolysis, procedural timing, treatment order, devices, antiplatelet or anticoagulant regimen, outcome definition, events, denominators, adjusted estimates, and adjustment variables.

For nonrandomized studies, we preferred estimates based on propensity weighting or matching, target-trial emulation, or comprehensive baseline adjustment. Models that conditioned on reperfusion, procedure duration, postprocedural antiplatelet treatment, bleeding, or other post-treatment variables were not selected as the primary total-effect estimate.

We created a cohort fingerprint from registry name, centers and countries, recruitment dates, eligibility criteria, sample size, author group, treatment distribution, and follow-up. TITAN, ETIS, STOP-CAD, CERES-TANDEM, SECURIS, and related center reports were cross-checked at the outcome and time-point level. Each cohort contributed only once to a given comparison and outcome. We prioritized the report with the clearest node definition, largest relevant sample, most complete follow-up, and effect model closest to the target estimand. CERES-TANDEM was presented separately from potentially overlapping multicenter registry analyses.

### 2.6. Outcomes

The co-primary outcomes were functional independence at approximately 90 days and sICH. Functional independence was defined as mRS 0–2 at 90 days. When 90-day data were unavailable, we used the assessment nearest to 90 days within a 60- to 120-day window. For sICH, we used each study’s prespecified definition within 24–72 h or during the index admission, prioritizing Heidelberg, SITS-MOST, ECASS, and then another explicit definition.

Secondary outcomes included ordinal mRS, mRS 0–1, successful reperfusion, 90-day mortality, any intracranial hemorrhage, cervical carotid patency or reocclusion, in-stent thrombosis, restenosis, recurrent ischemic stroke, and procedural complications.

### 2.7. Risk of Bias and Confidence in the Network Evidence

The randomized EASI pilot study was assessed by outcome with Cochrane RoB 2 [21]. Qin Yang and Xuzi Li independently assessed nonrandomized studies by outcome using ROBINS-I-aligned domains [22]. MuKe Zhou adjudicated disagreements. The assessment covered confounding, participant selection, strategy classification, deviations from the intended strategy, missing data, outcome measurement, and selective reporting. Prespecified confounders included age, prestroke function, baseline stroke severity, ASPECTS or core volume, collateral status, intracranial occlusion site, cervical etiology and severity, intravenous thrombolysis, and treatment timing. Study-level judgments were mapped to network comparisons through the contribution matrix. Additional result-specific assessments are presented in Table S6b–d.

CINeMA covered within-study bias, reporting bias, indirectness, imprecision, heterogeneity, and incoherence for B versus A, C versus A, and C versus B [23]. The primary range of clinical importance was OR 0.80–1.25. OR 0.90–1.11 and 0.75–1.33 were used as narrow and wide sensitivity ranges. Three reviewers approved the thresholds before the final assessment. Two reviewers independently completed RoB 2. CINeMA judgments were reviewed by the authors. Concerns arising from the same evidence limitation were grouped before deriving overall confidence to avoid duplicate downgrading.

### 2.8. Statistical Analysis

Binary outcomes were expressed as ORs with 95% CIs. For functional independence, an OR greater than 1 favored the first-listed strategy. For sICH, an OR greater than 1 indicated higher odds of bleeding with the first-listed strategy. The strict A/B/C network used a frequentist contrast-based random-effects model with a common heterogeneity variance, restricted maximum likelihood estimation, and preservation of within-study correlation in multiarm studies. Analyses were conducted in R version 4.4.1 with netmeta 3.3.1, meta 8.2.1, and metafor 4.8.0 [24].

We also pooled each direct comparison with a random-effects pairwise model. Hartung–Knapp intervals were used when at least three studies were available. A continuity correction of 0.5 was applied only to studies with a zero-event arm. Heterogeneity was summarized with tau squared and I squared, and prediction intervals were reported when estimable. Global incoherence was evaluated with a design-by-treatment interaction model. SIDE or direct-indirect separation was used for local assessment in closed loops. *p*-scores were descriptive and interpreted alongside relative effects and uncertainty intervals [25]. Network geometry and comparison-adjusted funnel plots followed established methods [18,26].

Sensitivity analyses excluded preprints, the conference abstract, reports with unclear treatment-group allocation, and dissection-only cohorts. We omitted one cohort at a time and substituted overlapping reports. We expanded the network to four treatment nodes by adding the intact D node while restricting cohort overlap. Adjusted C-versus-D evidence was separated into the CERES-TANDEM estimate and a non-CERES core. Harbord’s test was used to assess small-study effects for direct C-versus-B comparisons when at least 10 studies were available. Transitivity was assessed across age, baseline stroke severity, ASPECTS, cervical etiology, thrombolysis, treatment era, treatment order, and antiplatelet regimen. Two reviewers independently assessed these effect modifiers, with adjudication by MuKe Zhou.

### 2.9. Ethics

This review used published or publicly verifiable aggregate data and did not constitute human-participant research. Institutional review board approval and informed consent were not required.

### 2.10. Use of AI-Assisted Tools

OpenAI Codex was used as an assistive tool to check the statistical methods for internal consistency and methodological reasonableness. It did not make analytic decisions. The authors evaluated all suggestions and determined and verified the final methods, analyses, and interpretations.

## 3. Results

### 3.1. Study Selection and Network Composition

The five databases yielded 3323 records: 770 from PubMed, 1176 from Ovid Embase, 91 from CENTRAL, 630 from Web of Science, and 656 from Scopus. Deduplication removed 1805 records, leaving 1518 for title and abstract screening. Of these, 1410 were excluded. The remaining 108 record identifiers were consolidated into 101 reports sought for retrieval. One report could not be obtained, and 100 reports underwent full-text assessment.

**Figure 1:**
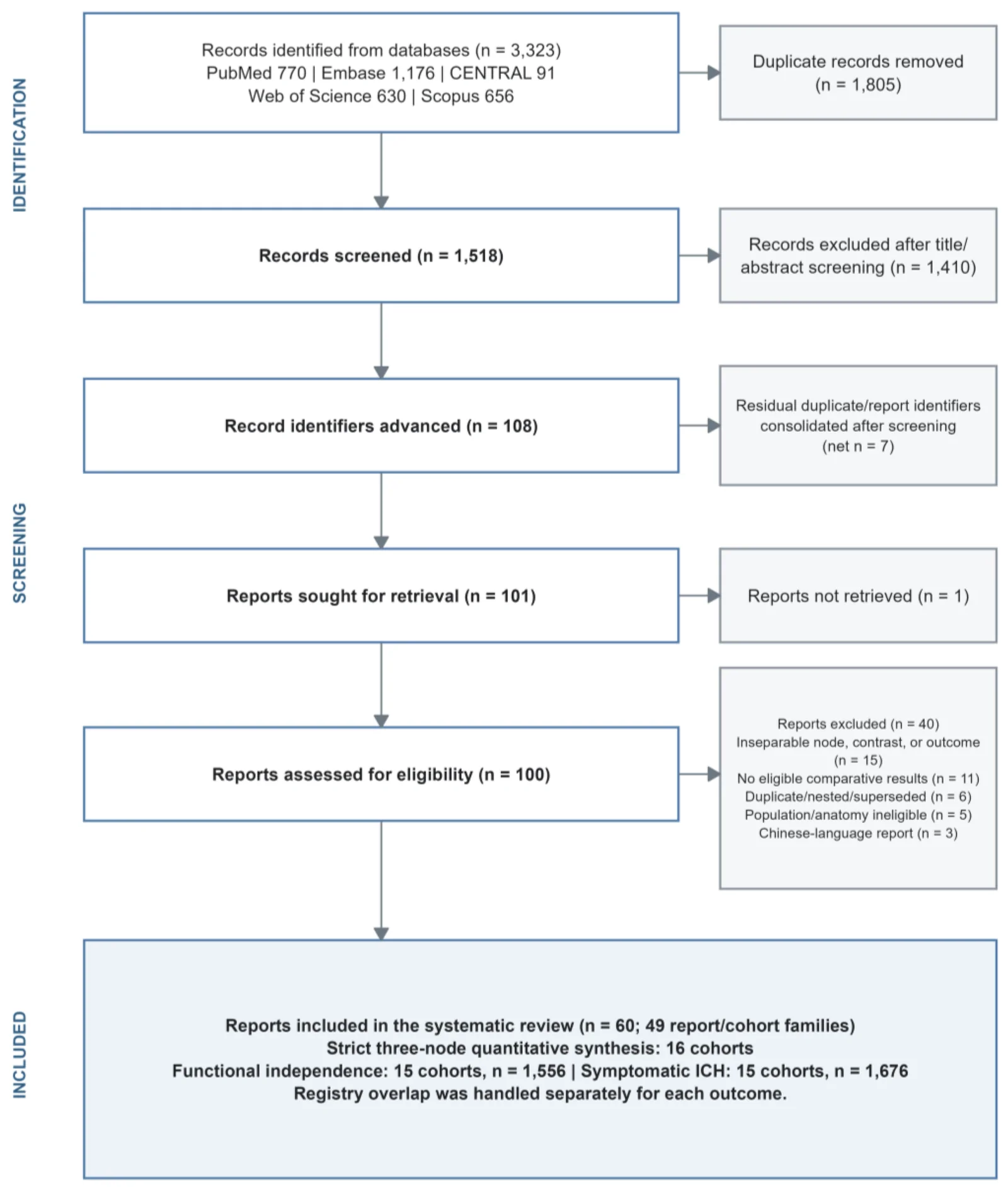
**PRISMA 2020 Flow Diagram.** Records identified from PubMed, Ovid Embase, Cochrane CENTRAL, Web of Science, and Scopus were deduplicated and screened. Residual consolidation converted 108 record identifiers to 101 reports sought for retrieval. Sixty reports representing 49 cohort or report families were included in the systematic review. The strict network contained 16 unique cohorts contributing at least one co-primary outcome.

Forty reports were excluded: 15 because the treatment node, comparison, or outcome could not be separated; 11 because no eligible comparison or outcome data were available; six because they were duplicate, nested, or superseded reports; five because the population or anatomic comparison was ineligible; and three because they were published in Chinese. Sixty reports representing 49 cohort or report families entered the systematic review (Figure 1). Report-level exclusions are provided in Table S4.

**Figure 2:**
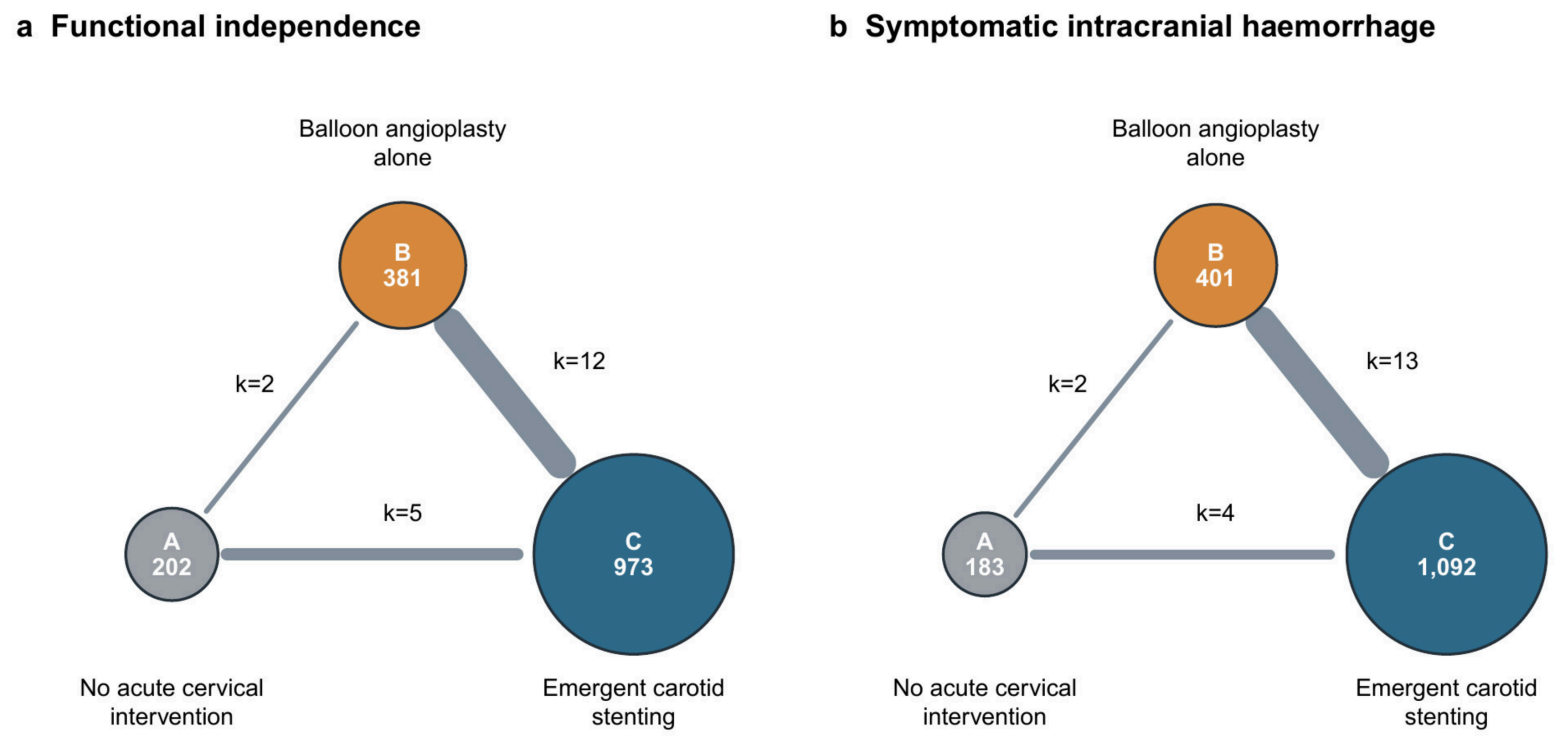
**Outcome-Specific Geometry of the Strict Three-Node Exploratory Network.** Panel (**a**) shows functional independence at approximately 90 days, and panel (**b**) shows symptomatic intracranial hemorrhage. Numeric node labels give the outcome-specific denominator, and node area is proportional to that denominator. Edge width and labels indicate the number of studies providing each direct comparison. A denotes no targeted acute cervical revascularization or deferred treatment, B denotes balloon angioplasty alone, and C denotes emergent carotid stenting.

Sixteen distinct cohorts contributed at least one co-primary outcome to the strict A/B/C network [27,28,29,30,31,32,33,34,35,36,37,38,39,40,41,42]. The functional-independence network contained 15 cohorts and 1556 outcome-evaluable patients: 202 in A, 381 in B, and 973 in C. The sICH network contained 15 cohorts and 1676 patients: 183 in A, 401 in B, and 1092 in C. The C-versus-B edge carried the most direct evidence, with 12 studies for functional independence and 13 for sICH. Two studies informed each A-versus-B edge (Figure 2). Outcome-specific arm counts and direct study estimates are provided in Table S7.

### 3.2. Study Characteristics and Transitivity

Key characteristics and synthesis roles of all 60 included reports are summarized in Table 1. Reports from the same or potentially overlapping cohort share a family ID and were not treated as independent cohorts. The strict network included single-center and multicenter studies from Europe, North America, and Asia. Most comparisons were retrospectively assembled from prospective registries. The evidence base also included prospective registry cohorts, a propensity-matched cohort, a conference abstract, and a preprint. Cervical etiology included atherosclerosis, dissection, or both. Antiplatelet practice ranged from delayed single or dual oral therapy to intravenous aspirin or glycoprotein IIb/IIIa bridging (Table 2).

**Table 1:** Included reports and key study characteristics.

Report [Reference]; ID	Family and Synthesis Role	Setting and Design	Population and Sample	Cervical Strategy and Co-Primary Data
Marko et al. (2022) [43]; L32	F01: ESCAPE-NA1 tandem-lesion subgroup Core non-CERES adjusted evidence (mRS 0–2); four-node sensitivity analysis (mRS 0–2 and sICH)	International multicenter ESCAPE-NA1 trial subgroup; observational comparison of acute cervical stenting versus no stenting within a randomized nerinetide/placebo trial.	Report total *n* = 115	C/A + B mixed; C; A + B mixed. Outcomes: 90-day mRS 0–2; sICH.
Divani et al. (2022) [44]; L33	F02: International Tandem Consortium/PICASSO family Selected family results: Farooqui 2023. No additional independent sample.	Multicenter registry analysis, 15 centers; conference abstract; recruitment dates NR.	PICASSO tandem-lesion population: 686 screened, 589 analyzed; stenting 363 and non-stenting 225 (reported arm total, 588; one-patient discrepancy).	Acute stenting vs. mixed non-stenting. Outcomes: 90-day mRS 0–2, sICH, reperfusion and mortality.
Wahood et al. (2022) [45]; L37	F04: US National Inpatient Sample administrative cohort. Descriptive in-hospital evidence only; 90-day mRS 0–2 and sICH not reported in the abstract.	United States HCUP-NIS; retrospective administrative-database analysis with discharge weights; conference abstract.	Tandem ICA/MCA occlusion treated with thrombectomy and CAS or angioplasty. Reported group estimates: C = 6632 and B = 3357 (sum 9989); national weighted estimates, not a verified unique-patient count.	B vs. C. Inpatient mortality, non-routine discharge and temporal utilization trends; no 90-day mRS 0–2 or sICH counts.
Garg et al. (2021) [46]; L38	F05: HCUP-NRD 2016–2017 family. Descriptive companion evidence; combined B + C revascularization cannot enter the A/B/C/D networks.	United States Nationwide Readmissions Database, 2016–2017; retrospective administrative cohort with propensity matching; conference abstract.	15,137 anterior-circulation LVO thrombectomy hospitalizations; 1214 received CAS/angioplasty during the same hospitalization; matched analysis 1063 pairs. Mean age 70.1 ± 14.6 years; 51.1% female.	Combined CAS/angioplasty vs. no cervical revascularization; timing is same hospitalization, not confirmed same procedure. Outcomes: in-hospital hemorrhagic transformation, mortality/discharge/resource use and 30-day readmission.
Gigliotti et al. (2021) [37]; L39	F06: Salem multicenter cohort Strict network: mRS 0–2 and sICH	United States; 2 centers; retrospective multicenter cohort with prospectively collected data; January 2012–May 2020	Report total n = 92. Cervical etiology: Mixed; dissection 5/92; exact atherosclerosis count unavailable	B: BA + MT; C: CAS + MT; sequence not reported. mRS 0–2: B66/C26; sICH: B66/C26.
Anadani et al. (2021) [47]; L40	F07: TITAN/ETIS registry family Contextual pooled TITAN/ETIS report; not added independently to the strict network. Selected TITAN result: Papanagiotou 2018.	Pooled French ETIS and international TITAN registries; TITAN January 2012–September 2016; ETIS January 2013–July 2019.	Anterior-circulation tandem occlusion treated with EVT; n = 603 (stenting 341; non-stenting 262).	Acute stenting vs. non-stenting. Outcomes: 90-day mRS 0–2 and 0–1, reperfusion, any ICH, sICH and parenchymal hematoma type 2.
Garg et al. (2021) [48]; L41	F05: HCUP-NRD 2016–2017 family Related HCUP-NRD 2016–2017 emergent-versus-delayed CAS report. Administrative inpatient outcomes; not a separate independent co-primary comparison.	United States Nationwide Readmissions Database, 2016–2017; retrospective administrative-database study; conference abstract.	AIS hospitalizations with anterior-circulation EVT and CAS: n = 1601 (emergent 1392; delayed 209).	Same-day CAS vs. CAS on days 2–90. Outcomes: in-hospital mortality, ICH, discharge disposition, ventilation, gastrostomy, length of stay and charges.
Garg et al. (2021) [49]; L42	F05: HCUP-NRD 2016–2017 family. Conference precursor to the HCUP-NRD B-versus-C publication by Garg 2021 (L44); no separate independent contribution. Outcomes were counted under L44.	United States Nationwide Readmissions Database, 2016–2017; retrospective administrative-database study; conference abstract.	AIS hospitalizations with same-day anterior-circulation EVT and CAS or angioplasty: n = 2042 (CAS 1391; angioplasty 651).	C vs. B. Outcomes: in-hospital ICH and mortality, ventilation, gastrostomy, length of stay, charges and 30-day readmission.
Kalmar et al. (2021) [35]; L43	F08: Kalmar multicenter cohort Strict network: mRS 0–2 and sICH	Hungary; 3 centers; retrospective analysis of a prospective registry; November 2017–December 2020	Report total n = 101. Cervical etiology: Atherothrombotic only; dissection excluded	B: BA → MT; C: CAS + BA → MT (generally antegrade). mRS 0–2: B29/C68; sICH: B29/C72.
Garg et al. (2021) [50]; L44	F05: HCUP-NRD 2016–2017 family; preferred full publication over conference precursor L42. Descriptive inpatient/readmission evidence; no 90-day mRS or sICH contribution and no additional independent L42 sample.	United States Nationwide Readmissions Database, 2016–2017; retrospective survey-weighted administrative cohort.	Reported national estimates: 2042 hospitalizations, C = 1391 and B = 651	Admission-day anterior-circulation EVT with carotid stenting (C) vs. angioplasty without same-day stenting (B); detailed occlusion anatomy cannot be verified from codes. Outcomes: index-admission ICH/death composite and components, discharge home, other complications/resource use and 30-day readmission. No 90-day mRS or specifically adjudicated sICH.
Li et al. (2021) [36]; L45	F09: Li 2021 multicenter propensity cohort Strict network: mRS 0–2 and sICH	China; 5 centers; retrospective analysis of prospective registries; January 2016–July 2019; propensity-matched analysis	Report total n = 75; Analysis n = 44. Cervical etiology: Atherosclerotic only	B: BA → MT; C: BA → MT with acute CAS; uniform CAS timing not reported. mRS 0–2: B22/C22; sICH: B22/C22.
Marnat et al. (2020) [51]; L46	F07: TITAN/ETIS registry family Contextual dissection-restricted TITAN/ETIS report; not added independently to the selected TITAN comparison.	24 comprehensive stroke centers; retrospective analysis of two prospective registries; January 2012–January 2019.	TITAN/ETIS cervical-dissection tandem occlusion: n = 136 (stenting 65; non-stenting 71).	Acute stenting vs. non-stenting. Outcomes: 90-day mRS 0–2, reperfusion, sICH and 90-day mortality.
Kim et al. (2020) [34]; L47	F10: Kim multicenter cohort Strict network: mRS 0–2 and sICH	Republic of Korea; 17 centers; retrospective analysis of prospective registries; January 2011–December 2015	Report total n = 75. Cervical etiology: Atherosclerosis 70/75; dissection 5/75	B: BA → MT; C: CAS → MT (50) or BA → MT → CAS (6). mRS 0–2: B19/C56; sICH: B19/C56.
Jadhav et al. (2019) [31]; L48	F11: STRATIS registry tandem-lesion substudy Strict network: mRS 0–2 and sICH	United States; 55 centers; prospective nonrandomized registry; August 2014–June 2016	Report total n = 147; Analysis n = 121. Cervical etiology: Mixed/unspecified; counts not reported	B: angioplasty only; C: acute CAS; sequence counts unavailable. mRS 0–2: B41/C73; sICH: B38/C69.
Dorado et al. (2019) [52]; L49	F12: Dorado single-center family Related later report; no additional independent strict-network sample. Selected family result: Dorado 2017.	Retrospective review of a prospective EVT registry over 10 years; location and calendar dates NR; conference abstract.	641 anterior-circulation LVO cases; 107 tandem occlusions; 95 underwent intracranial thrombectomy (stenting 30; non-stenting 65).	Stenting vs. angioplasty, aspiration or no cervical treatment. Outcomes: early neurological deterioration, 3-month mRS >2, ICA reocclusion and 24 h sICH.
Gomez-Escalonilla Escobar et al. (2019) [53]; L50	F13: TATTOO multicenter cohort Descriptive report only: strategy frequencies are reported, but no A/B/C-specific co-primary outcome counts or cervical-strategy effect estimate is available in the conference abstract.	Spain; multicenter retrospective cohort; January 2014–December 2017; conference abstract AS07-038.	172 tandem-occlusion patients	Cervical stenting 41.7%, angioplasty 29.2%, aspiration 7.7%, no cervical treatment 21.4%. Overall mRS ≤2 at 3 months 49.7%, sICH 7%, mortality at 3 months 8.1%, TICI 2b-3 77.3%.
Paipa Merchan et al. (2018) [54]; L52	F14: Bellvitge cohort. Descriptive secondary-outcome evidence with extractable reperfusion counts; no arm-specific mRS 0–2 or sICH data.	Hospital Universitari de Bellvitge, Barcelona, Spain; single-center cohort review, June 2010–October 2017; conference abstract.	120 tandem occlusions among 822 thrombectomy patients; A = 80, B = 22, C = 18. Critical cervical carotid stenosis/occlusion plus acute intracranial occlusion.	A/B/C. Intracranial TICI 2b-3 counts A = 53/80, B = 18/22, C = 13/18.
Li et al. (2018) [29]; L53	F15: Li 2018 single-center cohort Strict network: mRS 0–2 and sICH	China; 1 center; prospective registry cohort; January 2013–June 2016	Report total n = 37. Cervical etiology: Atherosclerosis 22/37; dissection 15/37	B: BA → MT; C: CAS → MT. mRS 0–2: B18/C19; sICH: B18/C19.
Ramakrishnan et al. (2018) [55]; L54	F16: Ramakrishnan single-center cohort. Descriptive reperfusion and mRS 0–1 evidence; no extractable mRS 0–2 or sICH data.	Riverside Regional Medical Center, United States; retrospective single-center series, July 2012–December 2017; conference abstract.	9 tandem-occlusion patients treated with intracranial mechanical thrombectomy; B = 3, C = 6.	Angioplasty alone vs. angioplasty plus acute CAS. TICI 2b-3: 67% vs. 100%; 90-day mRS ≤1: 0/3 vs. 3/6.
Papanagiotou et al. (2018) [30]; L55	F07: TITAN/ETIS registry family Strict network: mRS 0–2 and sICH	7 countries; 18 centers; retrospective pooled registry; recruitment dates not reported	Report total n = 482. Cervical etiology: Atherosclerosis 329/482; dissection 105/482; cardioembolic 48/482	A: MT only; B: angioplasty; C: acute CAS; sequence not reported. mRS 0–2: A108/B52/C322; sICH: A108/B52/C322.
Weill et al. (2018) [56]; L56	F17: Weill/CHUM single-center family. Companion to L57/L63 in the same 73-patient cohort; no independent contribution. Family outcomes were counted once.	CHUM Notre-Dame Hospital, Montreal, Canada; retrospective single-center study; December 2014–December 2016; conference abstract.	Tandem occlusion treated with EVT; n = 73 (stenting 26; non-stenting 47).	Acute stenting vs. mixed non-stenting. Outcomes: 90-day mRS 0–2, sICH, intracranial and ICA recanalization.
Weill et al. (2018) [57]; L57	F17: Weill/CHUM 73-patient family. Mixed-comparator narrative evidence; same cohort as L56 and L63, counted once and not independently pooled.	CHUM Notre-Dame Hospital, Montreal, Canada; retrospective single-center study, December 2014–December 2016; conference abstract.	73 tandem-occlusion EVT patients: acute stenting 26, no stenting 47; severe cervical ICA stenosis/occlusion with distal ICA and/or MCA occlusion.	C vs. source-defined non-stenting (A/B not separated). Reported C/non-stenting: 90-day mRS 0–2 46%/30%; sICH 8%/4%; TICI 2b-3 77%/79%; ICA recanalization 92%/9%.
Poppe et al. (2018) [58]; L58	F18: EASI randomized pilot family. Interim EASI report; no additional independent sample. The completed pilot was reported by Poppe 2020 (D00540).	CHUM, Montreal, Canada; single-center randomized EASI substudy, 2013–2015; interim conference abstract.	16 tandem-lesion patients among 138 EASI participants; acute stenting 9 and no stenting 7.	Acute stenting vs. no acute stenting; 90-day mRS 0–2 and mRS 5–6. Companion of the completed 24-patient randomized pilot.
Rodriguez-Villatoro et al. (2018) [59]; L59	F19: Vall d’Hebron prospective cohort. Descriptive report; co-primary outcome counts/effect estimates are unavailable.	Vall d’Hebron University Hospital, Barcelona, Spain; prospective consecutive cohort, April 2013–April 2017; conference abstract.	92 non-cardioembolic tandem-occlusion patients treated with EVT within 6 h: B = 40, C = 52; age 64.6 ± 12.6 years; IV rtPA 50/92.	Angioplasty alone vs. acute stenting (self-expanding or balloon-expanding, both C). Outcomes: 24 h ICA restenosis/occlusion and NIHSS change, discharge NIHSS, 3-month mRS and stroke recurrence, HT. No usable group mRS/sICH estimates.
Dorado et al. (2017) [42]; L62	F12: Dorado single-center family Strict network: mRS 0–2 and sICH	Country not reported in abstract; 1 center; prospective registry; March 2008–April 2016; conference abstract	Report total n = 68. Cervical etiology: Cervical ICA plus MCA occlusion; etiology not reported	B: isolated BA + MT; C: CAS ± BA + MT; sequence not reported. mRS 0–2: B32/C25; sICH: B40/C28.
Weill et al. (2017) [60]; L63	F17: Weill/CHUM 73-patient family. Earlier overlapping conference report of L56/L57; narrative companion, not independently pooled.	CHUM, Montreal, Canada; retrospective single-center EVT cohort, 2014–2016; conference abstract; later companion specifies December 2014–December 2016.	73 tandem-occlusion EVT patients: acute stenting 26 and no stenting/deferred treatment 47; same sample and outcomes as L56/L57.	C vs. non-stenting/deferred cervical intervention; A/B not separated. Same reported percentages as L57 for 90-day mRS 0–2, sICH and intracranial/ICA recanalization.
Motta et al. (2016) [61]; L65	F20: Motta single-center cohort. Mixed-comparator narrative evidence; no numerical eligible adjusted effect or arm-specific co-primary data.	Single stroke center, location not specified in the indexed abstract; retrospective cohort, 2012–2015; conference abstract.	60 acute ipsilateral ICA/MCA tandem occlusions; CAS 32, non-CAS 28. Atherothrombotic 51.7%, cardioembolic 30%, dissection 5%, undetermined 13.3%.	C vs. incompletely specified non-CAS cervical management. Three-month mRS analyzed by etiology; TICI and sICH used as regression covariates.
Marnat et al. (2016) [27]; F15	F21: RECOST dissection tandem subgroup Strict network: mRS 0–2 and sICH	France; 1 center; retrospective treatment-subgroup analysis of a prospective database; August 2009–April 2013	Report total n = 20. Cervical etiology: Dissection only (20/20)	A: MT only; C: MT → selective CAS when collaterals were inadequate. mRS 0–2: A15/C5; sICH: A15/C5.
Fahed et al. (2016) [62]; F16	F22: Fahed single-center registry. Descriptive source evidence only.	Fondation Ophtalmologique Adolphe de Rothschild, Paris, France; one treating center.	70 tandem-occlusion patients starting an EVT attempt: C = 37, D = 33	C: acute ICA stenting; D: no acute stent, with A/B composition not separable. Three-month mRS 0–2 and mortality, procedural TICI 2b–3, and HT at 24 h/within 72 h have arm counts in source Table 3.
Poppe et al. (2020) [63]; D00540	F18: EASI randomized pilot family Randomized pilot, reported separately; four-node sensitivity analysis (mRS 0–2 and sICH)	CHUM, Montreal, Canada; single-center randomized EASI pilot; April 2013–December 2018; 24 tandem-lesion patients randomized 1:1 to acute stenting or no acute stenting.	Report total n = 24	C/D; A; C. Outcomes: 90-day mRS 0–2; sICH.
Nolan et al. (2021) [64]; D00445	F23: Massachusetts General Hospital tandem cohort. Source-reported C-versus-D functional, symptomatic-hemorrhage and reperfusion effects are contextual only.	USA; Massachusetts General Hospital, Boston; 1 center; retrospective analysis of a prospectively maintained database; January 2011–September 2019.	62 tandem-lesion patients; 60 in treatment/outcome analyses after exclusion of two Merci-treated patients; 55 had accessible cervical ICAs (C34/A13/B8), with five inaccessible ICAs excluded from treatment-association analyses. Cervical etiology: atherosclerosis 46/62, dissection 16/62.	A: no acute cervical treatment, n = 13; B: angioplasty alone, n = 8; C: acute stenting, n = 34; stenting before or immediately after intracranial EVT. The source report compares C34 with pooled A + B21. Outcome: 90-day mRS 0–2.
Eker et al. (2018) [28]; D00589	F24: Bern + Montpellier atherosclerotic tandem cohort Strict network: mRS 0–2 and sICH	Switzerland/France; 2 centers; retrospective analysis of prospective databases; January 2010–September 2015	Report total n = 121. Cervical etiology: Atherosclerotic only (121/121)	A: MT with cervical stenting deferred 2–5 d; B: acute PTA; C: acute CAS. mRS 0–2: A14/B8/C96; sICH: A14/B9/C98.
Kang et al. (2019) [32]; D00577	F25: Kang single-center cohort Strict network: mRS 0–2 and sICH	Republic of Korea; 1 center; retrospective analysis of a prospective registry; April 2009–March 2017	Report total n = 62. Cervical etiology: Atherosclerotic only (62/62)	B: BA → MT; C: BA → MT → rescue CAS (31) or BA → CAS → MT (9). mRS 0–2: B22/C40; sICH: B22/C40.
Wallocha et al. (2019) [33]; D00580	F26: Wallocha single-center cohort Strict network: sICH	Germany; 1 center; retrospective analysis of a prospective database; April 2009–May 2016	Report total n = 163. Cervical etiology: Atherosclerotic only (163/163)	B: PTA + MT; C: stent-PTA + MT; mixed antegrade/retrograde order. mRS 0–2: Not available; sICH: B14/C149.
Troja et al. (2021) [65]; D01132	F27: Indiana University atherosclerotic tandem cohort Descriptive mixed/unclear nonstent comparator; no effect estimate or arm-specific outcome counts reported.	USA; Indiana University, Indianapolis; 1 center; retrospective consecutive-thrombectomy cohort; 2011–2019; conference abstract 12852.	69 patients with atherosclerotic tandem occlusion and severe cervical stenosis ≥70%; acute stent n = 35; no stent with persistent severe stenosis n = 34.	C35 vs. nonstent 34; prior balloon angioplasty in the nonstent group is not specified. Outcomes: carotid re-occlusion and neurological outcome/mRS at 90 days after discharge; reperfusion and length of stay. Only between-group *p* values are supplied (re-occlusion 0.434.
Yu et al. (2023) [38]; D00305	F28: single-center cohort Strict network: mRS 0–2 and sICH	China; 1 center; retrospective analysis of a prospective registry; January 2018–December 2021	Report total n = 98. Cervical etiology: Atherosclerotic only (98/98)	B: BA → MT; C: BA → MT → CAS after observation. mRS 0–2: B34/C64; sICH: B34/C64.
Kojima et al. (2023) [66]; D00283	F29: Kojima-Takasaki_2014–2022 Four-node sensitivity analysis (sICH only)	Japan; retrospective series at Gunma University and affiliated hospitals; January 2014–June 2022; 50 tandem-lesion EVT patients.	Report total n = 50	C: acute carotid stenting; D: mixed nonstenting management (12 angioplasty-only and 2 untreated cases). Outcomes: sICH within 14 days after the procedure.
Farooqui et al. (2023) [11]; D00285	F02: International Tandem Consortium/PICASSO family Sensitivity only: preferred PSM adjusts for periprocedural antiplatelets (mRS 0–2; sICH)	International Tandem Consortium; 17 centers; retrospective international multicenter cross-sectional cohort; January 2015–December 2020.	Report total n = 623	C/D; A + B mixed; C. Outcomes: 90-day mRS 0–2; sICH.
Sallustio et al. (2023) [67]; D00320	F30: IRETAS national registry. Descriptive source evidence only; TITAN is retained in the pooled analyses because shared centers and recruitment periods indicate possible partial overlap.	Italy; 56-center national registry; retrospective analysis of consecutive EVT cases treated within 6 h; January 2011–December 2017.	466 tandem-occlusion cases screened; 118 excluded for missing acute-CAS status; 348 analyzed (C122; D226). Outcome-specific N: mRS 0–2 and mortality C118/D211; sICH C116/D217.	Acute CAS (C) versus no acute CAS (D). D includes carotid angioplasty in 23/226 and is neither pure A nor pure B.
Madden et al. (2023) [68]; D00989	F31: Northern-Ireland-national-thrombectomy-centre_2014–2021 Contextual adjusted evidence (mRS 0–2)	Royal Victoria Hospital, Belfast, Northern Ireland, United Kingdom; observational analysis of prospectively recorded patients at a national thrombectomy center; 2014–2021; conference abstract 567.	Report total n = 96	C versus D (source-defined nonstenting comparator; A/B not separated). Outcomes: 90-day mRS 0–2.
Essibayi et al. (2023) [69]; D01000	F32: STAR family Descriptive A–C results; outcome-specific denominators unavailable. Companion D00943 provides the B–C report; the potentially shared C arm is not counted twice as independent evidence.	Multicenter Stroke Thrombectomy and Aneurysm Registry; retrospective registry analysis; 2010–2022; number of recruiting centers/countries not stated in the abstract; conference abstract WP164.	688 tandem-carotid cases among 9812 thrombectomies; reported A–C comparison comprises A444/C132 (576 patients). A propensity-matched analysis includes 129 per group.	A: MT alone, n = 444; C: emergent cervical stenting, n = 132. Ninety-day mRS 0–2, mortality, sICH and mTICI 2b–3 reported as percentages without outcome-specific n.
Bajrami et al. (2024) [70]; D00935	F33: Istanbul Aydin University cohort Descriptive three-node evidence; strategy-specific outcome events or effects are not published in the abstract.	Turkey; Istanbul Aydin University; 1 center; retrospective study of a prospectively maintained database; recruitment dates not reported; conference abstract 1414.	202 consecutive patients; 147 men	A: MT with no additional carotid procedure; B: balloon angioplasty; C: balloon-expandable or self-expandable stenting. Outcomes: mRS 0–2 at 3 months, sICH on 24 h imaging and ICA patency. Stent-subtype outcome counts are not supplied.
Essibayi et al. (2024) [71]; D00943	F32: STAR family Contextual B–C adjusted average treatment effects; companion to D01000, with likely shared C132. Published ATEs are not odds ratios and treatment coding is not explicit.	Multicenter Stroke Thrombectomy and Aneurysm Registry; retrospective analysis with inverse-propensity scoring and regression adjustment (IPSWR); recruiting countries/center count and dates not stated in this abstract.	244 tandem-occlusion patients: C132 (CAS plus BA) and B112 (BA alone). This report’s n is 244, not the 688 tandem cases cited in companion D01000.	B: BA alone, n = 112; C: BA plus CAS, n = 132. Ninety-day good functional outcome; mortality; sICH; successful reperfusion.
Abousleiman et al. (2025) [72]; D01439	F34: ETIS-France dissection report Contextual report only: the eligible tandem subgroup is identifiable, but its outcomes are not separable from isolated extracranial ICA disease in the published B-versus-C outcome tables.	France; ETIS registry, 33 centers; retrospective analysis; January 2015–January 2023.	160 dissection-related EVT cases (B26/C134), including 119 tandem occlusions (B19/C100) and 41 isolated extracranial ICA cases (B7/C34). The 119 is a subgroup count, not an analyzed outcome denominator.	Balloon angioplasty without subsequent stenting (B) versus acute stenting (C). Published 3-month mRS, mortality and 24 h sICH compare the full mixed 160-patient population; no tandem-only outcome counts are supplied.
Simon et al. (2026) [73]; D00199	F35: Maastricht Stroke Quality Registry Procedural-order context only: the analyzed comparison is acute stenting before versus after intracranial thrombectomy (C versus C), not different cervical-strategy nodes.	Netherlands; Maastricht tertiary center; retrospective analysis of prospectively registered consecutive patients; September 2020–January 2023.	505 EVT patients screened; 80 had tandem lesions (14 no acute cervical treatment, 16 angioplasty only, 50 acute stenting). Only the 50 stented patients were analyzed: stenting-before 31 versus stenting-after 19; 3-month mRS evaluable in 27 versus 19.	C-versus-C procedural sequence comparison. 3-month mRS 0–2 13/27 versus 9/19; ordinal mRS cOR 0.81 (0.29–2.30).
Kaba et al. (2025) [74]; D00893	F36: Kaba–Beyazal single-center cohort Secondary-outcome evidence only: discharge function, 24 h hemorrhagic transformation and reperfusion. No 90-day mRS or sICH endpoint is reported.	Turkey; Recep Tayyip Erdogan University, Rize; 1 center; retrospective cohort; recruitment dates not reported; conference abstract.	20 tandem ICA/intracranial-occlusion patients; B13/C7	B: emergent BA alone, n = 13; C: emergent carotid stenting, n = 7; intracranial aspiration thrombectomy in both. Outcomes: TICI 2b–3 at procedure end; hemorrhagic transformation at 24 h.
Florez et al. (2025) [75]; D00869	F37: Parc Taulí 2021–2025 cohort Descriptive B–C evidence; abstract reports arm sizes but no numerical clinical/procedural outcomes.	Spain; Parc Taulí Hospital, Sabadell; 1 center; retrospective observational study; 2021–2025; conference abstract A205.	39 patients with tandem intracranial/cervical ICA occlusion; B13/C26.	B: BA alone, n = 13; C: acute ICA stenting plus MT, n = 26. Ninety-day mRS and periprocedural complications were assessed, but the abstract gives no outcome counts, percentages or effect CIs.
Faybushevich et al. (2025) [40]; D01509	F38: Moscow-Yudin cohort Strict network: mRS 0–2 and sICH	Russian Federation; 1 center; comparative study; allocation described as random but methods and recruitment period not reported	Report total n = 94. Cervical etiology: Atherosclerotic only (94/94)	A: cervical crossing ± BA → MT, no acute revascularization; C: MT → CAS. mRS 0–2: A46/C48; sICH: A46/C48.
Özdemir et al. (2025) [39]; D00124	F39: Ege-University_2016–2024 Strict network: mRS 0–2	Turkey; 1 center; retrospective historical-control cohort from a prospective database; January 2016–January 2024	Report total n = 54. Cervical etiology: Atherosclerosis 49/54; dissection 5/54	A: MT without acute CAS; C: MT → CAS + BA (retrograde). mRS 0–2: A19/C35; sICH: Not available.
Sousa et al. (2025) [13]; D00141	F40: STOP-CAD international dissection cohort Sensitivity only: dissection-restricted population (mRS 0–2; sICH)	International STOP-CAD secondary cohort; 63 centers; retrospective multicenter analysis of dissection-related tandem occlusion.	Report total n = 328	C versus D (source-defined nonstenting comparator; A/B not separated). Outcomes: 90-day mRS 0–2; sICH.
Ezcurra-Díaz et al. (2025) [12]; D00087	F41: SECURIS/CICAT Catalonia. Descriptive source evidence only; not independently pooled alongside ASSIST and IRIS because centers and recruitment periods overlap.	Ten centers in Catalonia, Spain; retrospective analysis of the prospective mandatory CICAT registry, supplemented by clinical records; January 2017–March 2023.	578 adults: eCAS, 344; non-eCAS, 234. Anterior-circulation LVO with atheromatous cervical ICA stenosis ≥50%, prestroke mRS 0–3 and time from symptom onset or last seen well ≤24 h.	C versus unresolved non-eCAS D; the source does not establish an A/B split. Source Figure 2 yields strict 90-day mRS 0–2 counts of 154/344 versus 84/234.
Deliktas et al. (2025) [76]; D00185	F42: ETIS-France_2015–2023 Contextual adjusted evidence (sICH)	France; 30-center ETIS prospective registry, retrospective analysis of consecutive patients; January 2015–June 2023; 218 tandem-lesion patients with pretreatment ASPECTS 3–5 and known 90-day mRS.	Report total n = 218	C versus D (source-defined nonstenting comparator; A/B not separated). Outcomes: sICH.
Chen et al. (2026) [77]; D00029	F43: US Nationwide Readmissions Database 2016–2022 Contextual discharge/in-hospital outcomes only; no mRS 0–2 or mortality and no sICH endpoint. Not independently pooled with shorter overlapping NRD reports.	USA; Nationwide Readmissions Database; retrospective administrative cohort; 2016–2022.	Source abstract reports total 4928, but displayed groups A3371 + B541 + C1015 sum to 4927. Both source figures are reported, with the one-patient discrepancy noted.	A:EVT alone 3371; B:EVT + BA541; C:EVT + CAS1015. Outcomes: ordinal discharge functional status, in-hospital mortality, and any ICH during admission. Adjusted C–A effects are available; B–A is described only as nonsignificant.
Beltrán-Guaquetá et al. (2026) [78]; D00030	F44: Colombia single-centre_2016–2025 Sensitivity only: nonstandard follow-up (functional outcome at variable follow-up)	Colombia; single high-complexity referral center; retrospective cohort; October 2016–June 2025.	Report total n = 66	A + B mixed/C/C/D; A; C. Outcomes: Functional independence, mRS 0–2.
Romoli et al. (2026) [15]; D00067	F45: CERES-TANDEM_49-centres_2018–2024 Adjusted CERES evidence, reported separately because of overlap	International CERES-TANDEM cohort; 49 comprehensive stroke centers in Europe, North America and Singapore; retrospective longitudinal cohort; 1 January 2018–31 December 2024; estimand-based IPTW analysis.	Report total n = 4053	C versus D (source-defined nonstenting comparator; A/B not separated). Outcomes: 90-day mRS 0–2; sICH.
Cavalcante et al. (2026) [16]; D00081	F46: IRIS IPD: DEVT, DIRECT-MT, SKIP, MR-CLEAN-NO-IV, SWIFT-DIRECT and DIRECT-SAFE_2017–2021 Core non-CERES adjusted evidence and four-node sensitivity analysis (mRS 0–2 and sICH)	International individual-participant-data meta-analysis of six trials: DEVT, DIRECT-MT, SKIP, MR CLEAN-NO IV, SWIFT DIRECT and DIRECT SAFE; 2017–2021.	Report total n = 329	C/D; A + B mixed; C. Outcomes: 90-day mRS 0–2; sICH.
Ashouri et al. (2026) [14]; D00027	F47: ASSIST_NCT03845491 Four-node sensitivity analysis (mRS 0–2 and sICH)	ASSIST international registry; 71 sites in 11 countries; prospective multicenter registry subgroup analysis.	Report total n = 183	C versus D (source-defined nonstenting comparator; A/B not separated). Outcomes: 90-day mRS 0–2; sICH.
Rosales et al. (2026) [79]; D00808	F48: Rosales-Argentina_2015–2025 Sensitivity only: preprint (mRS 0–2; sICH)	Argentina; single comprehensive stroke center; retrospective observational registry; January 2015–July 2025; preprint, not peer reviewed.	Report total n = 111	A/B/C/C/D; A; B; C. Outcomes: 90-day mRS 0–2; sICH.
Han et al. (2026) [41]; D00806	F49: Han-Jia-Zhao SSRN cohort Strict network: mRS 0–2 and sICH	China; 1 center; retrospective cohort; January 2017–January 2025; preprint	Report total n = 112. Cervical etiology: Atherosclerotic only (112/112)	B: BA → MT; C: BA → MT → CAS. mRS 0–2: B38/C74; sICH: B38/C74.
Wichianrat et al. (2026) [80]; D00803	F50: Siriraj single-center cohort. Descriptive source evidence only; not quantitatively synthesized because seven separately labelled common-carotid cases cannot be separated from the eligible cervical-ICA population by acute strategy and outcome.	Thailand; Siriraj Hospital; retrospective stroke-registry cohort; January 2010–December 2023.	92 tandem cases: acute CAS20, angioplasty-only 24, no acute cervical intervention 48. Published C-versus-nonstenting comparison includes 79 patients (C20/nonstenting 59) after excluding 13 delayed carotid treatments.	Acute C20/B24/A48. Source Figures 4–6 directly report selected 79 C/non-stent patients: mRS 0–2, 13/20 versus 27/59; sICH, 1/20 versus 3/59. Thirteen delayed-treatment patients were excluded from that comparison.

**Notes.** Report IDs are internal identifiers used to link records across the Supplementary Materials; they are not reference numbers. A, no targeted acute cervical revascularization or deferred treatment; B, balloon angioplasty without permanent acute stenting; C, emergent cervical carotid stenting; D, source-defined mixture of no acute treatment and angioplasty; BA, balloon angioplasty; CAS, carotid artery stenting; MT, mechanical thrombectomy; NR, not reported.

**Table 2:** Characteristics of cohorts contributing to the strict three-node exploratory network meta-analysis.

Study (Year)	Setting and Design	Cervical-Lesion Etiology	Nodes and Acute Cervical Strategy	mRS 0–2 at 90 d, n	sICH, n	Periprocedural Antiplatelet/Antithrombotic Approach
Marnat (2016) [27], RECOST	France; 1 center; retrospective treatment-subgroup analysis of a prospective database; August 2009–April 2013	Dissection only (20/20)	A: MT only; C: MT → selective CAS when collaterals were inadequate	A15/ C5	A15/ C5	A: not reported; C: IV aspirin at CAS, then DAPT for 3 mo if 24 h imaging was acceptable
Dorado (2017) [42]	Country not reported in abstract; 1 center; prospective registry; March 2008–April 2016; conference abstract	Cervical ICA plus MCA occlusion; etiology not reported	B: isolated BA + MT; C: CAS ± BA + MT; sequence not reported	B32/ C25	B40/ C28	Not reported
Eker (2018) [28]	Switzerland/France; 2 centers; retrospective analysis of prospective databases; January 2010–September 2015	Atherosclerotic only (121/121)	A: MT with cervical stenting deferred 2–5 d; B: acute PTA; C: acute CAS	A14/ B8/ C96	A14/ B9/ C98	A: DAPT before deferred CAS; B: arm-specific regimen not reported; C: IV aspirin, clopidogrel after 24 h, then DAPT for 3–6 mo
Li (2018) [29]	China; 1 center; prospective registry cohort; January 2013–June 2016	Atherosclerosis 22/37; dissection 15/37	B: BA → MT; C: CAS → MT	B18/ C19	B18/ C19	IVT-dependent immediate or 24 h delayed aspirin + clopidogrel loading/maintenance; adherence by arm not reported
Papanagiotou (2018) [30], TITAN	7 countries; 18 centers; retrospective pooled registry; recruitment dates not reported	Atherosclerosis 329/482; dissection 105/482; cardioembolic 48/482	A: MT only; B: angioplasty; C: acute CAS; sequence not reported	A108/ B52/ C322	A108/ B52/ C322	Variable acute antithrombotics: C 256/322 received ≥1 agent; B 12/52; A 23/108
Jadhav (2019) [31], STRATIS	United States; 55 centers; prospective nonrandomized registry; August 2014–June 2016	Mixed/unspecified; counts not reported	B: angioplasty only; C: acute CAS; sequence counts unavailable	B41/ C73	B38/ C69	Exact regimen not reported; adjunctive pharmacotherapy was recorded in 15/80 stented patients
Kang (2019) [32]	Republic of Korea; 1 center; retrospective analysis of a prospective registry; April 2009–March 2017	Atherosclerotic only (62/62)	B: BA → MT; C: BA → MT → rescue CAS (31) or BA → CAS → MT (9)	B22/ C40	B22/ C40	Immediate or delayed SAPT/DAPT; arm-level counts reported
Wallocha (2019) [33]	Germany; 1 center; retrospective analysis of a prospective database; April 2009–May 2016	Atherosclerotic only (163/163)	B: PTA + MT; C: stent-PTA + MT; mixed antegrade/retrograde order	Not available	B14/ C149	B: no acute agent, aspirin after 24 h CT; C: IV aspirin, then aspirin ± clopidogrel after 24 h CT
Kim (2020) [34]	Republic of Korea; 17 centers; retrospective analysis of prospective registries; January 2011–December 2015	Atherosclerosis 70/75; dissection 5/75	B: BA → MT; C: CAS → MT (50) or BA → MT → CAS (6)	B19/ C56	B19/ C56	Center-specific heterogeneous protocols; GPI reserved for flow-limiting in-stent thrombosis
Gigliotti (2021) [37]	United States; 2 centers; retrospective multicenter cohort with prospectively collected data; January 2012–May 2020	Mixed; dissection 5/92; exact atherosclerosis count unavailable	B: BA + MT; C: CAS + MT; sequence not reported	B66/ C26	B66/ C26	Not standardized or reported by arm
Kalmar (2021) [35], STAY-ALIVE	Hungary; 3 centers; retrospective analysis of a prospective registry; November 2017–December 2020	Atherothrombotic only; dissection excluded	B: BA → MT; C: CAS + BA → MT (generally antegrade)	B29/ C68	B29/ C72	B: no periprocedural agent, aspirin after 24 h; C: IV aspirin before CAS, then DAPT for 3 mo after 24 h imaging
Li (2021) [36]	China; 5 centers; retrospective analysis of prospective registries; January 2016–July 2019; propensity-matched analysis	Atherosclerotic only	B: BA → MT; C: BA → MT with acute CAS; uniform CAS timing not reported	B22/ C22	B22/ C22	B: SAPT; C: IVT-dependent DAPT loading/delay, then DAPT for ≥3 mo
Yu (2023) [38]	China; 1 center; retrospective analysis of a prospective registry; January 2018–December 2021	Atherosclerotic only (98/98)	B: BA → MT; C: BA → MT → CAS after observation	B34/ C64	B34/ C64	Risk-adapted; C: tirofiban bridge to DAPT or delayed DAPT after stable CT
Faybushevich (2025) [40]	Russian Federation; 1 center; comparative study; allocation described as random but methods and recruitment period not reported	Atherosclerotic only (94/94)	A: cervical crossing ± BA → MT, no acute revascularization; C: MT → CAS	A46/ C48	A46/ C48	A: aspirin or aspirin + clopidogrel; C: pre-CAS aspirin + clopidogrel/ticagrelor
Özdemir (2025) [39]	Turkey; 1 center; retrospective historical-control cohort from a prospective database; January 2016–January 2024	Atherosclerosis 49/54; dissection 5/54	A: MT without acute CAS; C: MT → CAS + BA (retrograde)	A19/ C35	Not available	A: not reported; C: no immediate agent or loading dose, DAPT after 24 h if no hemorrhage
Han (2026) [41]	China; 1 center; retrospective cohort; January 2017–January 2025; preprint	Atherosclerotic only (112/112)	B: BA → MT; C: BA → MT → CAS	B38/ C74	B38/ C74	Not reported

All cohorts included patients with acute anterior-circulation tandem occlusion treated with intracranial thrombectomy. Outcome columns show analyzed participants by node and may differ from enrolment. A, no targeted acute cervical revascularization or deferred treatment; B, balloon angioplasty without acute stenting; C, acute carotid stenting. Li 2021 [36] uses the propensity-matched cohort (22 per arm); STRATIS uses the separable angioplasty-only subgroup; the two TITAN stenting strata were combined. BA indicates balloon angioplasty; CAS, carotid artery stenting; DAPT, dual antiplatelet therapy; GPI, glycoprotein IIb/IIIa inhibitor; ICA, internal carotid artery; IVT, intravenous thrombolysis; mRS, modified Rankin Scale; MT, mechanical thrombectomy; SAPT, single antiplatelet therapy; and sICH, symptomatic intracranial hemorrhage.

**Table 3:** Relative effects, treatment ranking, and CINeMA confidence in the strict three-node exploratory network.

Outcome	Network (Studies; Participants)	Comparison	Network OR (95% CI)	95% Prediction Interval	*p* Value	*p*-Score A/B/C	CINeMA Confidence
Functional independence (mRS 0–2 at approximately 90 days)	15; 1556	B vs. A	0.78 (0.43–1.42)	0.28–2.20	0.414	0.463/ 0.104/ 0.933	Low
Functional independence (mRS 0–2 at approximately 90 days)	15; 1556	C vs. A	1.35 (0.80–2.28)	0.50–3.63	0.265	0.463/ 0.104/ 0.933	Low
Functional independence (mRS 0–2 at approximately 90 days)	15; 1556	C vs. B	1.73 (1.22–2.45)	0.71–4.22	0.002	0.463/ 0.104/ 0.933	Low
Symptomatic intracranial hemorrhage	15; 1676	B vs. A	1.39 (0.57–3.43)	0.52–3.71	0.470	0.788/ 0.371/ 0.341	Low
Symptomatic intracranial hemorrhage	15; 1676	C vs. A	1.40 (0.66–2.97)	0.62–3.17	0.379	0.788/ 0.371/ 0.341	Very low
Symptomatic intracranial hemorrhage	15; 1676	C vs. B	1.01 (0.60–1.67)	0.58–1.75	0.985	0.788/ 0.371/ 0.341	Low

Odds ratios greater than 1 favor the treatment named first for functional independence and indicate greater sICH risk for safety. A denotes no acute cervical intervention or deferred treatment; B, balloon angioplasty alone; C, emergent carotid stenting. For functional independence, tau-squared = 0.144, I-squared = 33.9%, and global incoherence *p* = 0.782. For sICH, tau-squared approximated 0, I-squared = 0%, and global incoherence *p* = 0.259. For safety *p*-scores, higher values indicate lower sICH risk. Confidence was assessed with CINeMA.

Direct C-versus-B evidence contributed 93.4% of the network estimate for functional independence and 99.0% for sICH. These estimates depended little on the indirect path through A. Comparisons involving A were more exposed to cross-study differences in cervical etiology, treatment order, and antiplatelet regimen, particularly B versus A for sICH.

### 3.3. Risk of Bias

For the EASI randomized pilot, all five RoB 2 domains and the overall judgment were low risk for 90-day mRS 0–2. For sICH, the selective-reporting domain raised some concerns. The remaining domains were low risk, producing an overall judgment of some concerns (Table S3). Among 30 nonrandomized study-outcome assessments in the strict network, 26 were rated serious and four critical, with confounding driving the overall judgments (Table S6a). Judgments and supporting source details for the separate adjusted and expanded-network results are provided in Table S6b,c. Result-specific assessments for additional source reports are provided in Table S6d.

### 3.4. CINeMA Confidence Assessment

Confidence was low for three comparisons of functional independence. For sICH, confidence was low for B versus A and C versus B, and very low for C versus A (Table 3; Table S2; Figure S3).

### 3.5. Functional Independence at Approximately 90 Days

**Figure 3:**
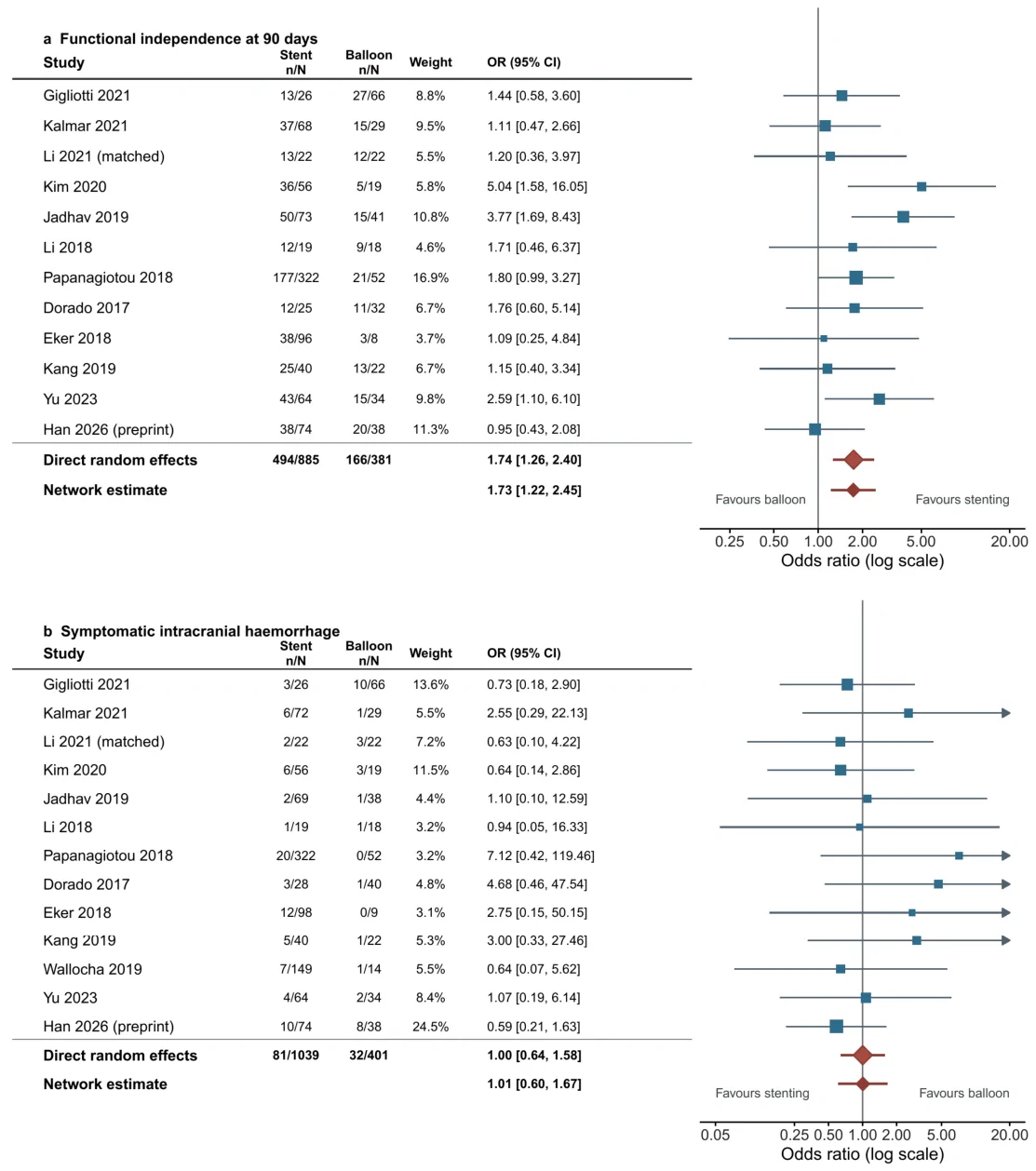
**Emergent Carotid Stenting Versus Balloon Angioplasty Alone for the Co-primary Outcomes.** Panel (**a**) shows functional independence at approximately 90 days and panel (**b**) shows symptomatic intracranial hemorrhage. Squares and horizontal lines are study odds ratios and 95% confidence intervals [28,29,30,31,32,33,34,35,36,37,38,41,42]. Square area reflects random-effects weight, and arrowheads denote confidence limits outside the plotted range. Red diamonds represent the direct and network summary odds ratios, with horizontal lines indicating their 95% confidence intervals. Bold rows present the corresponding summary results.The direct random-effects summary uses the separable C-versus-B studies, and the network estimate incorporates all strict A/B/C evidence. Odds ratios greater than 1 favor stenting for functional independence and favor balloon angioplasty for symptomatic intracranial hemorrhage.

In the strict three-node network, emergent carotid stenting was associated with higher odds of functional independence than balloon angioplasty alone (C versus B: OR, 1.73; 95% CI, 1.22–2.45; *p* = 0.002). Network heterogeneity was tau squared = 0.144, and I squared = 33.9%; the global design-by-treatment test gave *p* = 0.782. The direct random-effects estimate was similar across 12 studies and 1266 patients (OR, 1.74; Hartung–Knapp 95% CI, 1.26–2.40; I squared = 11.4%) (Figure 3; Table 3). The network 95% prediction interval was 0.71–4.22.

Comparisons involving A were less precise. The OR was 1.35 (95% CI, 0.80–2.28) for C versus A and 0.78 (95% CI, 0.43–1.42) for B versus A. Functional-outcome *p*-scores were 0.933 for C, 0.463 for A, and 0.104 for B (Figure S2). Local direct-indirect comparisons are reported in Table S9.

### 3.6. Symptomatic Intracranial Hemorrhage

No difference in sICH was detected between emergent stenting and balloon angioplasty alone (C versus B: OR, 1.01; 95% CI, 0.60–1.67; *p* = 0.985). The OR was 1.40 (95% CI, 0.66–2.97) for C versus A and 1.39 (95% CI, 0.57–3.43) for B versus A. Network tau squared approximated 0, I squared was 0%, and the global incoherence test gave *p* = 0.259. The direct estimate across 13 studies and 1440 patients was OR 1.00 (Hartung–Knapp 95% CI, 0.64–1.58) (Figure 3; Table 3). Although the point estimate was close to the null, the interval included clinically important lower and higher odds of sICH.

### 3.7. Robustness and Adjusted Evidence

**Figure 4:**
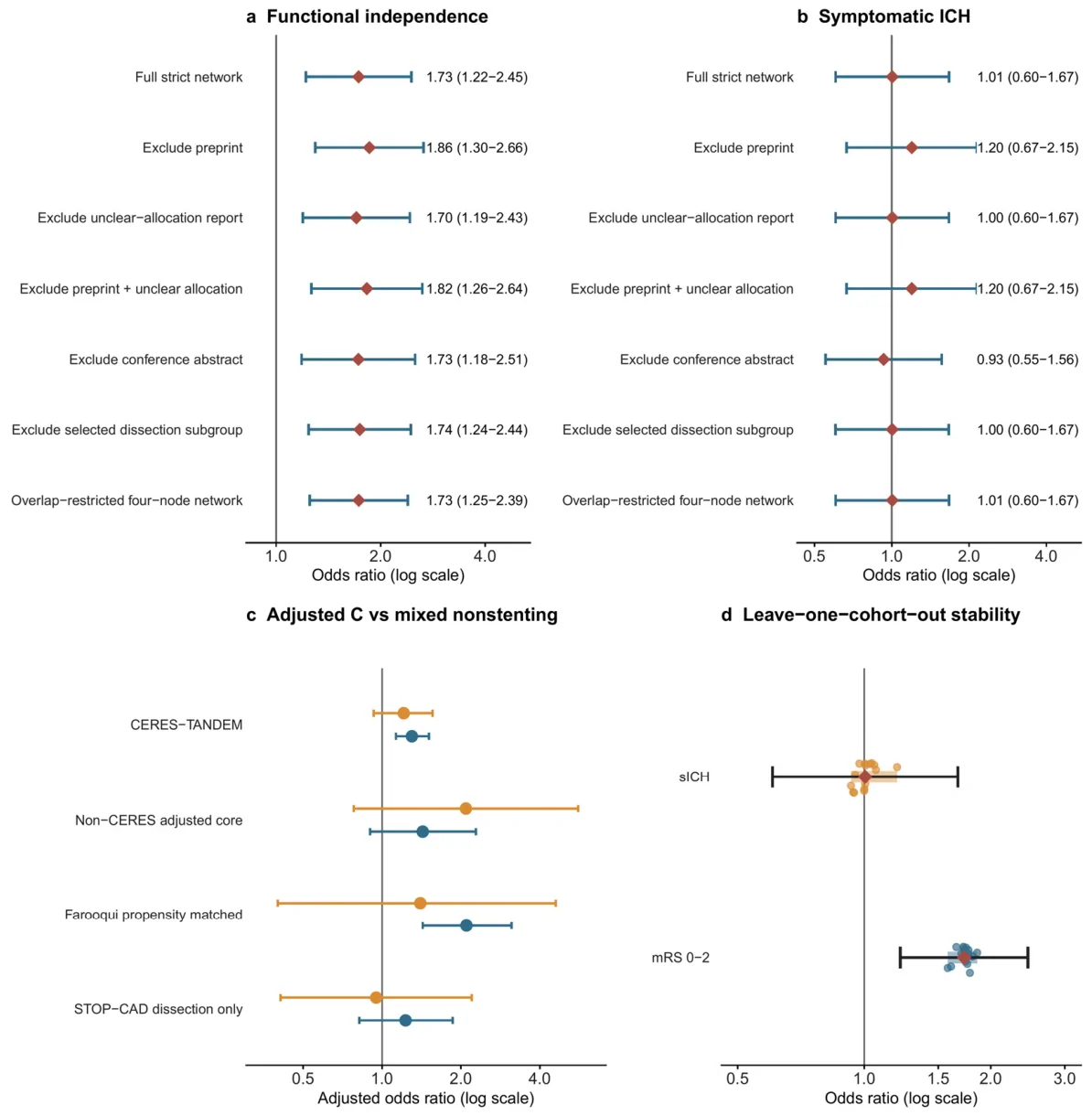
**Robustness and triangulation of the principal comparison.** Panels (**a**,**b**) show C-versus-B network estimates for functional independence and symptomatic intracranial hemorrhage (sICH) across prespecified and data-driven sensitivity analyses. Panel (**c**) shows adjusted C-versus-mixed-nonstenting evidence, with CERES-TANDEM separated from the non-CERES core to limit cohort overlap; blue denotes functional independence and orange denotes sICH. Panel (**d**) shows leave-one-cohort-out stability: points represent estimates after omitting individual cohorts, thick bars span their range, and diamonds with thin lines show the full-network estimates and 95% confidence intervals. mRS 0–2 denotes functional independence, and mRS denotes the modified Rankin Scale. Odds ratios greater than 1 favor stenting for functional independence and indicate greater sICH odds for safety.

For functional independence, every leave-one-cohort-out C-versus-B estimate remained above 1. The ORs ranged from 1.58 to 1.86, and all 95% CIs excluded 1. The corresponding sICH estimates ranged from 0.93 to 1.20, and all CIs included one (Figure 4; Figure S1). The functional estimate was 1.86 (95% CI, 1.30–2.66) after exclusion of the preprint, 1.70 (95% CI, 1.19–2.43) after exclusion of the report with unclear allocation, 1.82 (95% CI, 1.26–2.64) after both exclusions, 1.73 (95% CI, 1.18–2.51) after exclusion of the conference abstract, and 1.74 (95% CI, 1.24–2.44) after exclusion of the RECOST dissection subgroup. Harbord’s tests did not suggest funnel-plot asymmetry for functional independence (*p* = 0.644) or sICH (*p* = 0.433) (Figure S2).

The overlap-restricted four-node analysis incorporated intact mixed-nonstenting groups. For functional independence, 19 cohorts and 2194 patients yielded an OR of 1.71 (95% CI, 1.05–2.76) for C versus D and 1.73 (95% CI, 1.25–2.39) for C versus B. For sICH, 20 cohorts and 2375 patients yielded ORs of 1.18 (95% CI, 0.55–2.53) for C versus D and 1.01 (95% CI, 0.60–1.67) for C versus B. Added sources included EASI, the ESCAPE-NA1 tandem subgroup, the ASSIST registry, the IRIS individual-participant-data analysis, and the Kojima cohort [14,16,43,63,66].

Adjusted evidence was separated by overlap scenario. For functional independence, the non-CERES core combined IRIS and ESCAPE-NA1 (OR, 1.43; 95% CI, 0.90–2.28), whereas CERES-TANDEM yielded OR 1.30 (95% CI, 1.13–1.51). The CERES functional endpoint allowed mRS 0–2 or no worsening from the prestroke mRS and was broader than the strict-network endpoint. For sICH, the non-CERES core consisted of IRIS alone (OR, 2.09; 95% CI, 0.78–5.59), and CERES-TANDEM yielded OR 1.21 (95% CI, 0.93–1.56). The Farooqui propensity-matched sensitivity estimate was OR 2.10 (95% CI, 1.43–3.12) for functional independence and OR 1.40 (95% CI, 0.40–4.60) for sICH [11,15,16,43]. The 24-patient EASI randomized pilot yielded OR 0.91 (95% CI, 0.17–4.81) for functional independence and a continuity-corrected OR of 0.26 (95% CI, 0.01–7.03) for sICH [63]. Individual adjusted estimates and supporting outcome data are provided in Table S8.

### 3.8. Secondary Outcomes

Definitions and node allocation varied substantially for ordinal mRS, reperfusion, mortality, cervical patency, and procedural complications. These outcomes did not form stable networks and were not quantitatively pooled.

## 4. Discussion

Emergent carotid stenting was associated with greater odds of functional independence at approximately 90 days than balloon angioplasty alone. Network and direct estimates agreed, and all leave-one-cohort-out analyses retained this association with 95% CIs excluding 1. No difference in sICH was detected. The findings support a functional association without establishing equivalent safety.

The distinction between cervical strategies is central to interpreting this result. Balloon angioplasty is an active treatment, not an interchangeable alternative to leaving the cervical lesion untreated. Pooling these approaches can obscure the comparison between stenting and angioplasty because their selection mechanisms and procedural consequences differ [6,9,10]. Keeping them separate located the functional association within the comparison of two active strategies. A permanent stent may stabilize cervical inflow, limit elastic recoil and early reocclusion, and sustain the benefit of intracranial reperfusion [4,30]. Balloon angioplasty can provide rapid access but lacks the same structural support. The adjusted CERES-TANDEM result and the non-CERES core estimate also favored stenting [15,16,43]. However, both concerned source-defined mixed nonstenting strategies, and the non-CERES confidence interval included the null. These findings complement the separable C-versus-B evidence, and they do not estimate the same contrast.

The bleeding results require a different reading. Network and direct sICH ORs were 1.01 and 1.00, respectively, and estimates remained near the null when individual cohorts were omitted. Yet the network 95% CI of 0.60–1.67 encompassed clinically meaningful lower and higher bleeding odds. Stent selection and antiplatelet management thus remain a joint decision informed by infarct core, intravenous thrombolysis, baseline hemorrhagic risk, and the need to preserve cervical patency. Differences in comparator definitions may help explain the differing safety conclusions of previous meta-analyses [6,8,9,10]. A near-null pooled estimate should not remove these considerations from individual treatment decisions.

Functional outcomes also varied across settings: despite the favorable average association, the 95% prediction interval was 0.71–4.22. Cervical etiology, lesion anatomy, treatment order, infarct burden, and antiplatelet regimen are plausible sources of this variation. The findings support considering emergent stenting when it is technically feasible and an appropriate antiplatelet plan can be delivered.

Treatment-selection confounding remains the main limit on interpretation because nonrandomized comparisons dominated the evidence base. The outcome-specific overlap audit reduced repeated use of patients from TITAN, ETIS, STOP-CAD, CERES-TANDEM, and related multicenter registries [11,13,14,15,16], but could not address confounding within those cohorts. Unmeasured or inadequately controlled differences in collateral circulation, anticipated distal embolization risk, and vascular tortuosity may influence treatment selection and outcomes [11]. If patients with better prognostic or anatomical profiles were preferentially selected for stenting over angioplasty, selection could partly explain the observed functional association. Strict node definitions and separate presentation of event-count, adjusted, and randomized evidence preserved distinct estimands. The C-versus-B functional association also persisted after separate exclusions of the preprint, conference abstract, report with unclear allocation, and dissection-only cohort. These checks support the stability of the association, not a causal treatment effect. Direct evidence involving node A was sparse, and comparisons with angioplasty or stenting were imprecise, with low or very low CINeMA confidence. Confidence intervals crossed the null for functional independence and sICH. This uncertainty limits conclusions about the relative benefits and risks of deferred or medical management. sICH definitions and antiplatelet regimens were not consistent across cohorts.

Randomized trials that prespecify standardized antiplatelet regimens for each treatment arm are needed to limit confounding related to antiplatelet management. Patients should be stratified by atherosclerosis versus dissection, infarct burden, and thrombolysis status. CASES and EASI-TOC are positioned to test this treatment pathway [81,82]. The present analysis identifies emergent stenting versus balloon angioplasty alone as the most informative contrast and provides quantitative benchmarks for those trials.

## 5. Conclusions

In adults undergoing thrombectomy for anterior-circulation tandem occlusion, emergent carotid stenting was associated with greater 90-day functional independence than balloon angioplasty alone, without a detected difference in sICH. The functional association persisted across sensitivity analyses, although all studies in the strict network had serious or critical risk of bias. Randomized trials with prespecified antiplatelet protocols are needed to establish comparative functional outcomes and hemorrhagic risk.

## Data Availability

Report-level screening decisions, study characteristics, study-level risk-of-bias assessments, and tabulated individual-study outcome data are provided in the Supplementary Materials, together with the complete database search strategies. The review protocol and amendment summary are provided in Supplementary File S1. The full aggregate extraction records, data dictionary, cohort-overlap audit, and R analysis code are available from the corresponding author on reasonable request. No individual-participant data were obtained for this review.

## Supplementary Materials

The following supporting information can be downloaded at: https://www.mdpi.com/article/10.3390/brainsci16101128/s1. The Supplementary Appendix contains the following items. Table S1. Robustness analyses and adjusted evidence for acute cervical management strategies. Table S2. Final CINeMA domain judgements for the strict three-node exploratory network [23]. Table S3. Final RoB 2 assessment of the EASI randomized pilot study [21,63]. Table S4. Reports excluded after full-text assessment and reasons for exclusion [83,84,85,86,87,88,89,90,91,92,93,94,95,96,97,98,99,100,101,102,103,104,105,106,107,108,109,110,111,112,113,114,115,116,117,118,119,120,121,122]. Table S5. Catalogue and characteristics of included and companion reports [11,12,13,14,15,16,27,28,29,30,31,32,33,34,35,36,37,38,39,40,41,42,43,44,45,46,47,48,49,50,51,52,53,54,55,56,57,58,59,60,61,62,63,64,65,66,67,68,69,70,71,72,73,74,75,76,77,78,79,80]. Table S6a. Outcome-specific risk-of-bias judgements for the nonrandomized strict-network studies. Table S6b. Additional nonrandomized result-level risk-of-bias assessments. Table S6c. Source-based grounds for the additional risk-of-bias assessments [22,123]. Table S6d. Source evidence and assessment coverage for additional report families. Table S7. Individual-study event counts and effects in the strict A/B/C network. Table S8a. Source-reported adjusted effects by analysis role. Table S8b. Additional co-primary event-count evidence and the randomized pilot. Table S8c. Individual-study secondary and contextual binary outcomes. Table S8d. Source-reported secondary effects and matched sensitivity estimates. Table S8e. Available ordinal mRS distributions and summaries. Table S8f. Available continuous outcome and follow-up summaries. Table S8g. Source-reported single-arm cervical-device outcomes. Table S8h. Additional source-level results and synthesis roles. Registry records support the assessment of potential cohort overlap [124,125,126]. Table S9. Local direct–indirect evidence separation in the strict network. Figure S1. Leave-one-cohort-out analyses for emergent carotid stenting versus balloon angioplasty alone. Each row omits one cohort from the strict three-node network. The dashed vertical line is the full-network estimate and the solid line is the null value. Figure S2. Treatment ranking and comparison-adjusted funnel plots. Panels A and B show P-scores for functional outcome and safety. Panels C and D show comparison-adjusted funnel plots. Figure S3. Contribution structure and final CINeMA confidence. Panel A shows the percentage contribution of each direct comparison to the six network estimates. Panel B shows the final ratings for six CINeMA domains and overall confidence. Domain cells use separate colours for no, some, and major concerns. Overall-confidence cells show low or very-low confidence as labelled. Supplementary Search Strategies. Complete search strategies for PubMed, Ovid Embase, Cochrane CENTRAL, Web of Science Core Collection, and Scopus. Supplementary File S1 is provided separately and contains the review protocol and amendment summary [1,8,9,12,15,16,17,18,19,22,23,81,82,127,128].
