# Supplementary material for: Acute Cervical Carotid Management During Thrombectomy for Tandem Occlusion: A Systematic Review and Network Meta-Analysis
Source: Brain Sci. 2026 Oct 19;16(10):1128. doi: 10.3390/brainsci16101128 (PMC13702421; doi:10.3390/brainsci16101128)
Supplement: Supplementary file 1 [file brainsci-16-01128-s001.zip › brainsci-4579424-supplementary.pdf]

SUPPLEMENTARY APPENDIX

Acute Cervical Carotid Management During Thrombectomy for Tandem Occlusion: A Systematic Review and Network Meta-analysis

Supplementary Methods

Node A comprised no targeted acute cervical revascularization or deferred treatment; node B comprised balloon angioplasty without a permanent acute carotid stent; node C comprised emergent cervical carotid stenting. Source-defined mixtures of no acute treatment and angioplasty were retained as node D and were not split. Registry overlap was audited separately for each outcome and time point using recruitment period, centres, eligibility criteria, authorship, sample size, and treatment distribution. CERES-TANDEM was reported separately from potentially overlapping registry analyses.

The primary CINeMA clinical-importance range was OR 0.80-1.25; OR 0.90-1.11 and 0.75-1.33 were used as narrow and wide sensitivity ranges. Two reviewers independently completed 36 domain judgements and agreed on all ratings; MuKe Zhou confirmed the final results. Concerns arising from the same evidence limitation were grouped before downgrading, while independent concern clusters were retained.

Table S1. Robustness analyses and adjusted evidence for acute cervical management strategies

Section	Analysis	Comparison	Functional independence result	sICH result	Evidence structure and overlap control
Central estimates	Strict three-node exploratory NMA	C vs B	15 cohorts; n=1,556; OR 1.73 (95% CI 1.22-2.45); P=0.0022; network PI 0.71-4.22	15 cohorts; n=1,676; OR 1.01 (95% CI 0.60-1.67); P=0.985; network PI 0.58-1.75	Unadjusted A/B/C event-count network; no adjusted estimates connect all three strict nodes
Central estimates	Direct random-effects meta-analysis (Hartung-Knapp)	C vs B	12 studies; n=1,266; OR 1.74 (95% CI 1.26-2.40); P=0.0031; tau-squared=0.042; I-squared=11.4%; PI 1.00-3.02 (unrounded lower bound 0.998)	13 studies; n=1,440; OR 1.00 (95% CI 0.64-1.58); P=0.986; tau-squared=0; I-squared=0%; PI 0.64-1.58	Uses only studies with direct, separable B and C arms
Influence	Leave-one-cohort-out NMA	C vs B	15 iterations; OR range 1.58-1.86; every 95% CI excluded 1.00	15 iterations; OR range 0.93-1.20; every 95% CI included 1.00	Each cohort was omitted once from the strict network; iteration-specific sample sizes vary
Report-status sensitivity	Exclude preprint	C vs B	14 cohorts; n=1,444; OR 1.86 (95% CI 1.30-2.66)	14 cohorts; n=1,564; OR 1.20 (95% CI 0.67-2.15)	Excludes Han 2026 preprint
Allocation sensitivity	Exclude unclear-allocation report	C vs B	14 cohorts; n=1,462; OR 1.70 (95% CI 1.19-2.43)	14 cohorts; n=1,582; OR 1.00 (95% CI 0.60-1.67)	Excludes the Moscow report with unclear allocation
Combined report-quality sensitivity	Exclude preprint and unclear-allocation report	C vs B	13 cohorts; n=1,350; OR 1.82 (95% CI 1.26-2.64)	13 cohorts; n=1,470; OR 1.20 (95% CI 0.67-2.15)	Excludes both the preprint and unclear-allocation report; the Dorado conference abstract remains included
Publication-status sensitivity	Exclude conference abstract	C vs B	14 cohorts; n=1,499; OR 1.73 (95% CI 1.18-2.51)	14 cohorts; n=1,608; OR 0.93 (95% CI 0.55-1.56)	Excludes Dorado 2017; all remaining strict-network reports have full text
Small-study effects	Harbord regression test of funnel-plot asymmetry	Direct C vs B	12 studies; P=0.644	13 studies; P=0.433	No asymmetry was detected; the test is descriptive at these study counts
Adjusted evidence	CERES-TANDEM dominant cohort, reported alone	C vs mixed D	1 cohort; adjusted OR 1.30 (95% CI 1.13-1.51); P=0.001	1 cohort; adjusted OR 1.21 (95% CI 0.93-1.56); P=0.150	Kept separate to avoid probable overlap; the functional endpoint was mRS 0-2 or unchanged from prestroke status
Adjusted evidence	Non-CERES adjusted core	C vs mixed D	2 independent reports; OR 1.43 (95% CI 0.90-2.28); P=0.130; tau-squared=0; I-squared=0%	1 independent report; OR 2.09 (95% CI 0.78-5.59); P=0.142	Functional analysis pools IRIS and ESCAPE-NA1; sICH is IRIS alone; not combined with CERES-TANDEM
Nonstandard-adjustment sensitivity	International Tandem Consortium propensity-matched estimate	C vs mixed D	OR 2.10 (95% CI 1.43-3.12); P<0.001	OR 1.40 (95% CI 0.40-4.60); P=0.621	Sensitivity only: matching included periprocedural antiplatelet treatment, which may follow the cervical strategy; not pooled into the adjusted core
Population sensitivity	STOP-CAD dissection-only cohort	C vs mixed D	n=270; adjusted OR 1.23 (95% CI 0.82-1.86); P=0.315	n=324; adjusted OR 0.95 (95% CI 0.41-2.20); P=0.913	Sensitivity only: restricted to tandem occlusion caused by cervical dissection; not pooled into the adjusted core
Expanded raw sensitivity	Overlap-restricted four-node network	C vs mixed D	19 cohorts; n=2,194; OR 1.71 (95% CI 1.05-2.76); P=0.030	20 cohorts; n=2,375; OR 1.18 (95% CI 0.55-2.53); P=0.665	Adds only EASI, IRIS, ASSIST and ESCAPE-NA1 for functional independence, plus Kojima for sICH; excludes CERES-TANDEM, Farooqui and STOP-CAD; D remains intact
Randomized evidence	EASI randomized pilot, reported alone	C vs mixed D	8/13 vs 7/11; OR 0.91 (95% CI 0.17-4.81)	0/13 vs 1/11; continuity-corrected OR 0.26 (95% CI 0.01-7.03)	Single 24-participant pilot; D comprised 2 balloon-angioplasty and 9 no-targeted-treatment participants; also contributes to the expanded raw sensitivity but is shown here separately for design-specific context

The strict A/B/C network uses published arm-level events and is exploratory. Adjusted C-versus-D analyses retain the source-defined mixed nonstenting comparator. CERES-TANDEM is reported separately to reduce cohort overlap. Farooqui propensity-score-matched estimates are sensitivity evidence because the matching set included periprocedural antiplatelet treatment. CI indicates confidence interval; HK, Hartung-Knapp; OR, odds ratio; and PI, prediction interval.

Table S2. Final CINeMA domain judgements for the strict three-node exploratory network

Outcome	Comparison	Within-study bias	Reporting bias	Indirectness	Imprecision	Heterogeneity	Incoherence	Overall confidence	Basis for overall confidence
mRS 0-2	B vs A	Some concerns	No concerns	No concerns	Some concerns	No concerns	No concerns	Low	Two independent concern clusters: within-study bias and imprecision
mRS 0-2	C vs A	Some concerns	No concerns	No concerns	Some concerns	No concerns	No concerns	Low	Two independent concern clusters: within-study bias and imprecision
mRS 0-2	C vs B	No concerns	No concerns	No concerns	No concerns	Major concerns	No concerns	Low	Major concerns for heterogeneity
sICH	B vs A	Some concerns	No concerns	No concerns	Some concerns	No concerns	No concerns	Low	Two independent concern clusters: within-study bias and imprecision
sICH	C vs A	Major concerns	No concerns	No concerns	Some concerns	No concerns	No concerns	Very low	Major within-study bias plus an independent imprecision concern
sICH	C vs B	No concerns	No concerns	No concerns	Major concerns	No concerns	No concerns	Low	Major concerns for imprecision

Two reviewers independently judged all 36 outcome-comparison domains and reached complete agreement; MuKe Zhou confirmed the final ratings. Overall confidence was derived after grouping concerns that arose from the same evidence limitation, avoiding duplicate downgrading while retaining independent concern clusters.

For C versus B, serious- and critical-risk contributions were 92.8% and 7.2% for functional independence, and 94.7% and 5.3% for sICH (Table S6a).

Table S3. Final RoB 2 assessment of the EASI randomized pilot study

Study	Outcome	Domain	Reviewer 1	Reviewer 2	MuKe Zhou final	Final rationale
EASI randomized pilot	90-day mRS 0-2	D1 Randomization process	Low risk	Low risk	Low risk	The cervical strategy was explicitly randomized 1:1, and no evidence indicated failure of the allocation process.
EASI randomized pilot	90-day mRS 0-2	D2 Deviations from intended interventions	Low risk	Low risk	Low risk	All 24 participants remained in the intention-to-treat analysis; procedural nonreceipt of stenting did not remove participants from their assigned groups.
EASI randomized pilot	90-day mRS 0-2	D3 Missing outcome data	Low risk	Low risk	Low risk	The 90-day outcome was available for all randomized participants.
EASI randomized pilot	90-day mRS 0-2	D4 Measurement of the outcome	Low risk	Low risk	Low risk	The same prespecified mRS definition and assessment schedule were applied to both groups, with no evidence of differential ascertainment.
EASI randomized pilot	90-day mRS 0-2	D5 Selection of the reported result	Low risk	Low risk	Low risk	The reported functional endpoint, time point, and denominators were internally consistent across the report.
EASI randomized pilot	90-day mRS 0-2	Overall	Low risk	Low risk	Low risk	All five domains were judged at low risk.
EASI randomized pilot	24-hour sICH	D1 Randomization process	Low risk	Low risk	Low risk	The cervical strategy was explicitly randomized 1:1, with no evidence that allocation was compromised.
EASI randomized pilot	24-hour sICH	D2 Deviations from intended interventions	Low risk	Low risk	Low risk	All randomized participants were analysed in their assigned groups.
EASI randomized pilot	24-hour sICH	D3 Missing outcome data	Low risk	Low risk	Low risk	sICH was reported for all 24 randomized participants.
EASI randomized pilot	24-hour sICH	D4 Measurement of the outcome	Low risk	Low risk	Low risk	ECASS-2 criteria and 24-hour imaging were applied consistently across groups.
EASI randomized pilot	24-hour sICH	D5 Selection of the reported result	Some concerns	Some concerns	Some concerns	No analysis plan specific to the second randomization was available to verify the prespecified safety analysis.
EASI randomized pilot	24-hour sICH	Overall	Some concerns	Some concerns	Some concerns	Some concerns in selection of the reported result determined the overall judgement.

Judgements are outcome-specific and refer to the effect of assignment to the acute cervical strategy. Two reviewers independently completed the assessment, and MuKe Zhou approved the final judgements.

Figure S1. Leave-one-cohort-out analyses for emergent carotid stenting versus balloon angioplasty alone. Each row omits one cohort from the strict three-node network. The dashed vertical line is the full-network estimate and the solid line is the null value.

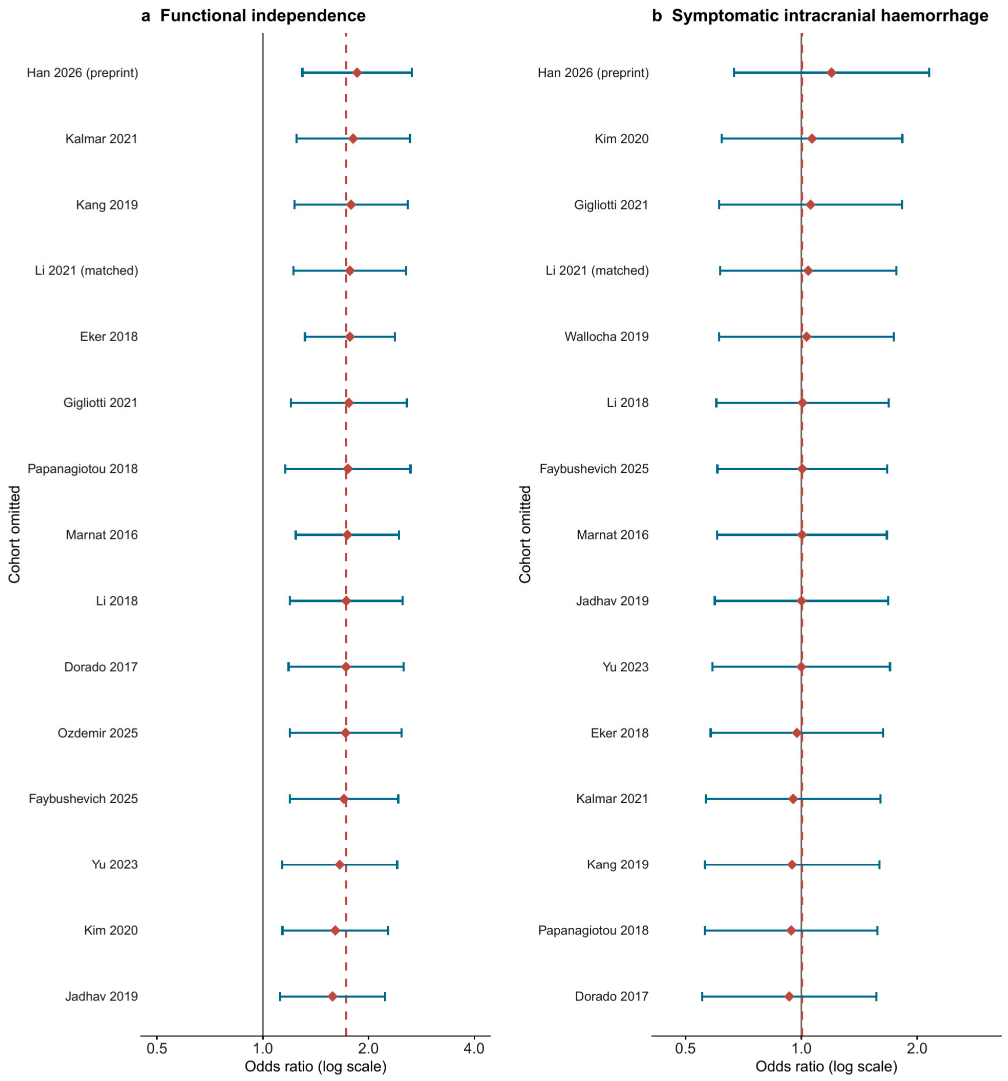

Figure S2. Treatment ranking and comparison-adjusted funnel plots. Panels A and B show P-scores for functional outcome and safety; panels C and D show comparison-adjusted funnel plots. Rankings are descriptive and should be interpreted with relative effects and uncertainty intervals.

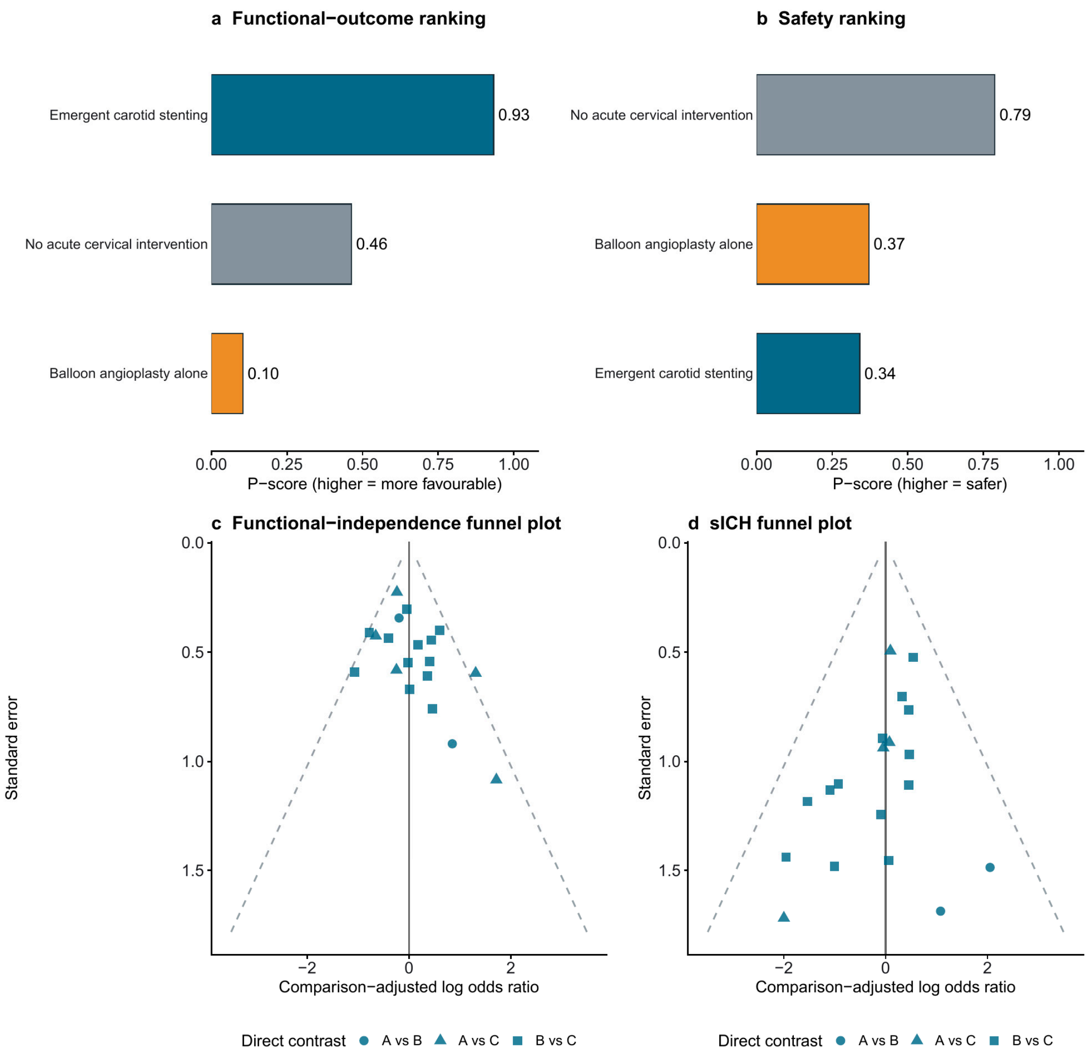

Figure S3. Contribution structure and final CINeMA confidence. Panel A shows the percentage contribution of each direct comparison to the six network estimates. Panel B shows the final ratings for six CINeMA domains and overall confidence. Domain cells use separate colours for no, some, and major concerns; overall-confidence cells show low or very-low confidence as labelled.

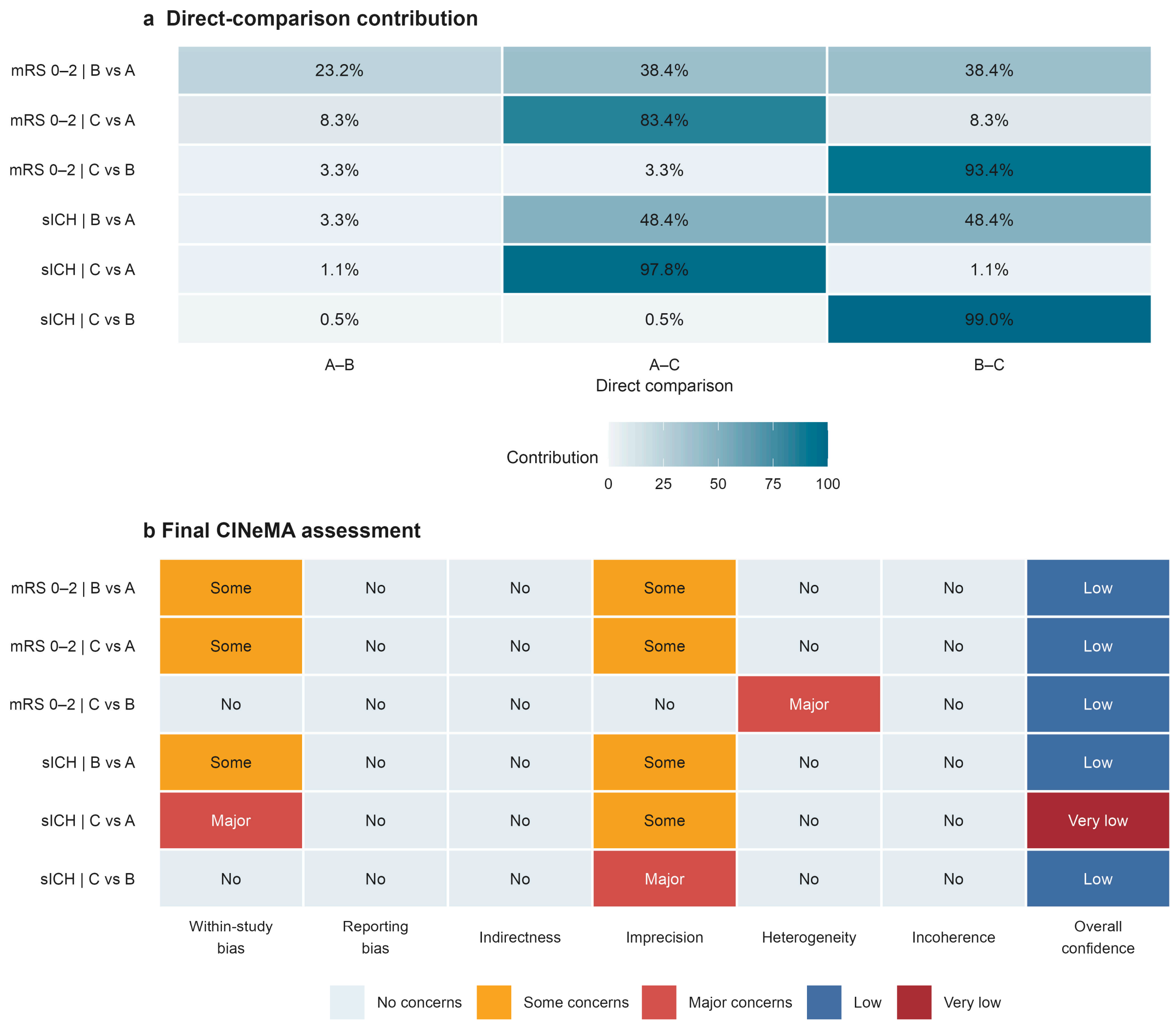

Supplementary Search Strategies

Qin Yang ran all five database searches on 25 August 2026 using one self-contained strategy per platform. The searches covered 1 January 2015 through 25 August 2026 and used no language or document-type restriction. Reference lists of included reports and relevant reviews were checked, and forward-citation searches were conducted in Web of Science and Scopus.

Database search yields before deduplication (all searches run on 25 August 2026 by Qin Yang)

Database	Records
PubMed	770
Ovid Embase	1,176
Cochrane CENTRAL	91
Web of Science Core Collection	630
Scopus	656
Total	3,323

Search coverage was 1 January 2015 through 25 August 2026. No database language filter was used. Reports published in Chinese were excluded during study selection.

Search 1. PubMed

(("Stroke"[Mesh] OR "Brain Ischemia"[Mesh] OR "Cerebral Infarction"[Mesh] OR stroke*[tiab] OR "acute ischemic stroke"[tiab] OR "acute ischaemic stroke"[tiab] OR "cerebral infarct*"[tiab] OR "brain infarct*"[tiab]) AND ("tandem occlusion*"[tiab] OR "tandem lesion*"[tiab] OR "tandem stenosis"[tiab] OR (tandem[tiab] AND (occlusion*[tiab] OR lesion*[tiab] OR stenosis[tiab])) OR (((cervical[tiab] OR extracranial[tiab]) AND ("internal carotid"[tiab] OR carotid[tiab]) AND (occlusion*[tiab] OR stenosis[tiab])) AND ((intracranial[tiab] OR "large vessel"[tiab]) AND (occlusion*[tiab] OR thrombus[tiab])))) AND (("Thrombectomy"[Mesh] OR "Endovascular Procedures"[Mesh] OR thrombectom*[tiab] OR "mechanical thrombectomy"[tiab] OR "endovascular treatment"[tiab] OR "endovascular therapy"[tiab] OR "endovascular intervention*"[tiab] OR EVT[tiab] OR "stent retriever*"[tiab] OR "aspiration thrombectomy"[tiab]) OR ("Angioplasty"[Mesh] OR "Stents"[Mesh] OR "carotid stent*"[tiab] OR "emergent stent*"[tiab] OR "emergency stent*"[tiab] OR eCAS[tiab] OR angioplast*[tiab] OR "carotid revasculari*"[tiab] OR "deferred treatment"[tiab] OR "no stent*"[tiab] OR nonstent*[tiab] OR "non-stent*"[tiab]))) AND ("2015/01/01"[dp] : "2026/08/25"[dp])

Search 2. Ovid Embase

((exp ischemic stroke/ OR exp cerebral ischemia/ OR exp brain infarction/ OR (stroke* OR acute ischemic stroke OR acute ischaemic stroke OR cerebral infarct* OR brain infarct*).ti,ab,kw.) AND ((tandem occlusion* OR tandem lesion* OR tandem stenosis OR (tandem adj3 (occlusion* OR lesion* OR stenosis*))).ti,ab,kw. OR ((((cervical OR extracranial) adj3 (internal carotid OR carotid) adj3 (occlusion* OR stenosis*)) AND ((intracranial OR large vessel) adj3 (occlusion* OR thrombus)))).ti,ab,kw.) AND (exp thrombectomy/ OR exp catheter thrombectomy/ OR exp endovascular surgery/ OR exp carotid artery stenting/ OR exp angioplasty/ OR exp stent/ OR (thrombectom* OR mechanical thrombectomy OR endovascular treatment OR endovascular therapy OR endovascular intervention* OR EVT OR stent retriever* OR aspiration thrombectomy OR carotid stent* OR emergent stent* OR emergency stent* OR eCAS OR angioplast* OR carotid revasculari* OR deferred treatment OR no stent* OR nonstent* OR non-stent*).ti,ab,kw.)) AND (2015* OR 2016* OR 2017* OR 2018* OR 2019* OR 2020* OR 2021* OR 2022* OR 2023* OR 2024* OR 2025* OR 2026*).yr.

Search 3. Cochrane CENTRAL

((stroke* OR "acute ischemic stroke" OR "acute ischaemic stroke" OR "cerebral infarct*" OR "brain infarct*"):ti,ab,kw) AND (("tandem occlusion*" OR "tandem lesion*" OR "tandem stenosis" OR (tandem NEAR/3 (occlusion* OR lesion* OR stenosis*))):ti,ab,kw OR (((cervical OR extracranial) NEAR/3 ("internal carotid" OR carotid) NEAR/3 (occlusion* OR stenosis*)) AND ((intracranial OR "large vessel") NEAR/3 (occlusion* OR thrombus))):ti,ab,kw) AND ((thrombectom* OR "mechanical thrombectomy" OR "endovascular treatment" OR "endovascular therapy" OR "endovascular intervention*" OR EVT OR "stent retriever*" OR "aspiration thrombectomy" OR "carotid stent*" OR "emergent stent*" OR "emergency stent*" OR eCAS OR angioplast* OR "carotid revasculari*" OR "deferred treatment" OR "no stent*" OR nonstent* OR "non-stent*"):ti,ab,kw)

Search 4. Web of Science Core Collection

TS=((stroke* OR "acute ischemic stroke" OR "acute ischaemic stroke" OR "cerebral infarct*" OR "brain infarct*") AND ("tandem occlusion*" OR "tandem lesion*" OR "tandem stenosis" OR (tandem NEAR/3 (occlusion* OR lesion* OR stenosis*)) OR (((cervical OR extracranial) NEAR/3 ("internal carotid" OR carotid) NEAR/3 (occlusion* OR stenosis*)) AND ((intracranial OR "large vessel") NEAR/3 (occlusion* OR thrombus)))) AND (thrombectom* OR "mechanical thrombectomy" OR "endovascular treatment" OR "endovascular therapy" OR "endovascular intervention*" OR EVT OR "stent retriever*" OR "aspiration thrombectomy" OR "carotid stent*" OR "emergent stent*" OR "emergency stent*" OR eCAS OR angioplast* OR "carotid revasculari*" OR "deferred treatment" OR "no stent*" OR nonstent* OR "non-stent*")) AND PY=(2015-2026)

Search 5. Scopus

TITLE-ABS-KEY((stroke* OR "acute ischemic stroke" OR "acute ischaemic stroke" OR "cerebral infarct*" OR "brain infarct*") AND ("tandem occlusion*" OR "tandem lesion*" OR "tandem stenosis" OR (tandem W/3 (occlusion* OR lesion* OR stenosis*)) OR (((cervical OR extracranial) W/3 ("internal carotid" OR carotid) W/3 (occlusion* OR stenosis*)) AND ((intracranial OR "large vessel") W/3 (occlusion* OR thrombus)))) AND (thrombectom* OR "mechanical thrombectomy" OR "endovascular treatment" OR "endovascular therapy" OR "endovascular intervention*" OR EVT OR "stent retriever*" OR "aspiration thrombectomy" OR "carotid stent*" OR "emergent stent*" OR "emergency stent*" OR eCAS OR angioplast* OR "carotid revasculari*" OR "deferred treatment" OR "no stent*" OR nonstent* OR "non-stent*")) AND PUBYEAR > 2014 AND PUBYEAR < 2027

Table S4. Reports excluded after full-text assessment and reasons for exclusion.

Report citation	Reason for exclusion
L31. Veunac L, Saliou G, Knebel JF, Bartolini B, Puccinelli F, Michel P, et al. Revascularization of carotid artery occlusion using stenting versus non stenting in endovascular management of tandem occlusion stroke. J Clin Neurosci. 2022;98:15-20. doi: 10.1016/j.jocn.2022.01.036.	Comparator population does not meet the protocol tandem cervical-lesion definition.
L35. Wu, Y.-M, Lin, D.-L, Yi, T.-Y, Lin, X.-H, Chen, R.-C, Pan, Z.-N, et al. Carotid artery stenting during procedure of emergency endovascular treatment in patients with carotid artery tandem lesions: a clinical efficacy analysis. Academic Journal of Naval Medical University. 2022;43(1):72-79. doi: 10.16781/j.0258-879x.2022.01.0072.	Chinese-language publication; excluded under the language eligibility criterion.
L36. Zhang, D.-H, Liu, Y.-F, Kang, M.-J, Wang, N, Sun, J, Wen, C.-M. Analysis of safety and efficacy of emergent carotid artery stenting in patients with acute anterior circulation stroke with tandem lesion treated with mechanical thrombectomy. Chinese Journal of General Practice. 2022;20(7):1143-1146. doi: 10.16766/j.cnki.issn.1674-4152.002543.	Chinese-language publication; excluded under the language eligibility criterion.
L51. Takezawa H, Katsumori T, Tamura A, Fujii A, Ogata A, Goto Y, et al. Which is better endovascular therapy for extracranial internal or common carotid artery atherosclerotic occlusions: Stenting or angioplasty alone?. International Journal of Stroke. 2018;13(2 Supplement 1):125.	Only five of seven patients had protocol-defined tandem lesions, and outcomes are not separated for tandem versus non-tandem cases; the eligible subgroup cannot be extracted.
L60. Janjua N, Tse-Chang K, Jones K, Woolfolk K, Panichpisal K, Keaslier D, et al. Extracranial carotid revascularization may have higher mortality rate in acute stroke intervention. Stroke. 2017;48(Supplement 1):no pagination.	Only 24/34 patients had tandem lesions and node-specific outcomes within the tandem subgroup are not extractable; the reported comparison also combines several cervical revascularization techniques.
L61. Behme D, Molina CA, Selim MH, Ribo M. Emergent Carotid Stenting After Thrombectomy in Patients With Tandem Lesions. Stroke. 2017;48(4):1126-1128. doi: 10.1161/strokeaha.117.016182.	Controversies-in-Stroke opinion/case-vignette article, not an original comparative cohort.
F6. Blassiau A, Gawlitza M, Manceau PF, Bakchine S, Serre I, Soize S, et al. Mechanical Thrombectomy for Tandem Occlusions of the Internal Carotid Artery-Results of a Conservative Approach for the Extracranial Lesion. Front Neurol. 2018;9(NOV):928. doi: 10.3389/fneur.2018.00928.	Cervical-strategy outcomes are not separable by A, B and C.
F7. Akpınar S, Gelener P. Endovascular treatment of acute tandem occlusion strokes and stenting first experience. J Clin Neurosci. 2018;47:328-331. doi: 10.1016/j.jocn.2017.09.010.	Small case series; six stented and two non-stented patients are not analyzed as a comparative A/B/C cohort.
F8. Santos R, Granja M.F, Almeida R.A, Aguilar-Salinas P, Lima J, Sauvageau E, et al. Single-center experience in the treatment of acute ischemic stroke due to tandem occlusions. Interventional Neurology. 2017;6(Supplement 1):27.	Outcomes are organized by extracranial-first versus intracranial-first procedural sequence and include heterogeneous cervical treatments; no node-specific comparison is available.
F9. Diaz Urrea C, Rayon-Aledo J.C, Lopez Hernandez N, Gallego Leon J.I, Beltran I, Llorente Ayuso L, et al. Endovascular treatment of tandem occlusion acute stroke patients: A cohort from hospital general de alicante. European Stroke Journal. 2017;2(1 Supplement 1):245-246.	Single cohort description with no extractable outcomes by A/B/C; delayed and acute procedures are reported only descriptively.
F10. Eker OF, Panni P, Dargazanli C, Marnat G, Arquizan C, Machi P, et al. Anterior Circulation Acute Ischemic Stroke Associated with Atherosclerotic Lesions of the Cervical ICA: A Nosologic Entity Apart. AJNR Am J Neuroradiol. 2017;38(11):2138-2145. doi: 10.3174/ajnr.a5404.	Compares atherosclerotic tandem lesions with dissection or isolated intracranial occlusion rather than comparing cervical treatment nodes.

Report citation	Reason for exclusion
F11. Shao Q, Zhu L, Li T, Wang Z, Li L, Bai W, et al. Management of tandem internal carotid and middle cerebral arterial occlusions with endovascular multimodal reperfusion therapy. Int J Neurosci. 2016;126(12):1077-83. doi: 10.3109/00207454.2015.1121387.	Six-patient multimodal case series without a comparative A/B/C analysis.
F12. Gelener P, Akpinar S. Endovascular treatment of acute tandem occlusion strokes. International Journal of Stroke. 2016;11(Supplement 3):173.	Seven-patient conference case series without node-specific comparative outcomes; precursor/duplicate family of the 2018 Akpinar report.
F13. Kurre W, Bansemir K, Aguilar Pérez M, Martinez Moreno R, Schmid E, Bäzner H, et al. Endovascular treatment of acute internal carotid artery dissections: technical considerations, clinical and angiographic outcome. Neuroradiology. 2016;58(12):1167-1179. doi: 10.1007/s00234-016-1757-z.	Acute-dissection series compares tandem with non-tandem anatomy and treatment sequence, not A/B/C cervical strategies.
F14. Grigoryan M, Haussen DC, Hassan AE, Lima A, Grossberg J, Rebello LC, et al. Endovascular Treatment of Acute Ischemic Stroke Due to Tandem Occlusions: Large Multicenter Series and Systematic Review. Cerebrovasc Dis. 2016;41(5-6):306-12. doi: 10.1159/000444069.	Multicentre case series/systematic review without a mutually exclusive cervical-treatment comparison suitable for the network.
D00660. Labeyrie MA, Ducroux C, Civelli V, Reiner P, Cognat E, Aymard A, et al. Endovascular management of extracranial occlusions at the hyperacute phase of stroke with tandem occlusions. J Neuroradiol. 2018;45(3):196-201. doi: 10.1016/j.neurad.2017.10.003.	The unit of analysis is 64 approaches rather than 49 mutually exclusive patients; approaches could recur in the same patient during the same procedure or admission, so independent A/B/C arm data cannot be formed.
D00511. Vu-Dang L, Nguyen QA, Nguyen-Thi-Thu T, Tran AT, Le-Chi C, Le-Hoang K, et al. Endovascular Treatment for Acute Tandem Occlusion Stroke: Results from Case Series of 17 Patients. Ann Indian Acad Neurol. 2020;23(1):78-83. doi: 10.4103/aian.aian_464_18.	The non-stent sequence combines balloon treatment with rescue/possible stenting; protocol nodes are not separable.
D00763. Maurer CJ, Joachimski F, Berlis A. Two in One: Endovascular Treatment of Acute Tandem Occlusions in the Anterior Circulation. Clin Neuroradiol. 2015;25(4):397-402. doi: 10.1007/s00062-014-0318-2.	Arm-specific outcomes for the small non-stent group are not reported.
D00845. Li, W, Feng, X, Wang, K, Huang, K, Sun, G, Chen, R, et al. The efficacy of balloon dilation angioplasty and stent implantation in treating patients with acute anterior tandem occlusion caused by internal carotid artery atherosclerosis. Journal of Interventional Radiology (China). 2024;33(6):593-598. doi: 10.3969/j.issn.1008-794x.2024.06.002.	Chinese-language publication; excluded under the language eligibility criterion.
D00176. Rodriguez Calienes A, Galecio-Castillo M, Petersen NH, Ribo M, Farooqui M, Hassan AE, et al. Mediation Analysis of Acute Carotid Stenting in Tandem Lesions: Effect on Functional Outcome in a Multicenter Registry. Neurology. 2024;103(3):e209617. doi: 10.1212/wnl.0000000000209617.	Companion mediation analysis of the same 2015-2020 multicentre cohort reported in JAMA Network Open 2023. It is not counted as an independent study; its mediator-adjusted estimate is not the selected total-effect estimate.
D00946. Olvany J, Ashouri Y, Miralbes S, Naravetla B, Spiotta A, Loehr C, et al. CLINICAL AND ANGIOGRAPHIC OUTCOME IN PATIENTS WITH TANDEM OCCLUSION THROMBECTOMY: ANALYSIS FROM THE ASSIST INTERNATIONAL REGISTRY. Journal of NeuroInterventional Surgery. 2024;16(Supplement 1):A26. https://jnis.bmj.com/content/16/Suppl_1/A26	Conference predecessor of the 2026 peer-reviewed ASSIST report, with the same 183 tandem-lesion patients and arm sizes. The full article was selected instead.

Report citation	Reason for exclusion
D00947. Ezcurra-Diaz G, Cardona P, Rodriguez-Villatoro N, Doncel-Moriano A, Flores-Pina B, Laia Figueras-Aguirre G, et al. EMERGENT CAROTID ARTERY STENTING DURING ENDOVASCULAR TREATMENT IN PATIENTS WITH ACUTE ISCHAEMIC STROKE. European Stroke Journal. 2024;9(1 Supplement):108. https://academic.oup.com/esj/article/9/1_suppl/3/8269153	Earlier CICAT/SECURIS conference report with a broader and partly ineligible population that also included isolated acute ICA occlusion. It is superseded by the 2025 SECURIS full article restricted to tandem lesions.
D01510. Faybushevich, A.G, Galustyan, Kh.V, Chernyaev, M.V, Gitelzon, E.A, Sysoev, V.M. Endovascular treatment of patients with acute ischemic stroke caused by tandem lesion of the internal carotid artery. Grudnaya i Serdechno-Sosudistaya Khirurgiya. 2025;67(2):150-158. doi: 10.24022/0236-2791-2025-67-2-150-158.	Same 94-patient Moscow cohort, same group sizes and principal outcome values as DOI 10.23934/2223-9022-2025-14-1-89-95. Duplicate report; one article was selected for each outcome.
D00880. Galustyan H, Faybushevich A, Sysoev V. TCT-1018 Endovascular Treatment of Tandem Occlusions in Anterior Circulation Stroke. Journal of the American College of Cardiology. 2025;86(17 Supplement):B435-B436. https://www.jacc.org/toc/jacc/86/17_Supplement	Updated conference report of the same Moscow/Yudin treatment programme, with 99 rather than 94 patients. It overlaps the two peer-reviewed 94-patient reports; no independent non-overlapping comparison is established.
D00177. Mendes GN, Jacquin G, Katsanos AH, Singh N, Stotts G, Ferguson DB, et al. Safety of acute internal carotid artery stenting during endovascular thrombectomy in patients with acute ischemic stroke: a retrospective analysis of the OPTIMISE registry. J Neurointerv Surg. 2025;17(e2):e281-e287. doi: 10.1136/jnis-2024-021915.	The comparator is the entire EVT-only anterior-circulation registry rather than a tandem-lesion cohort; most of the 3,875 controls did not have an eligible cervical tandem lesion, so the population and comparison are not separable.
D00271. Yahia MI, Marnat G, Finitsis S, Sibon I, Olivot JM, Pop R, et al. Acute carotid stenting plus aspirin during thrombectomy of tandem occlusions: A matched case-control study. Interv Neuroradiol. 2025;31(4):510-516. doi: 10.1177/15910199231175375.	All eligible tandem-lesion patients received acute stenting; controls had isolated intracranial occlusions rather than tandem lesions. This cannot estimate a cervical-treatment-node contrast within the target population.
D00214. Havlíček R, Šaňák D, Černík D, Neradová J, Leško N, Gdovinová Z, Köcher M, Cihlář F, Malik J, Fedorko J, et al. Predictors of Good Clinical Outcome After Endovascular Treatment for Acute Ischemic Stroke due to Tandem Lesion in Anterior Circulation: Results from the ASCENT Study. Cardiovasc Intervent Radiol. 2024;47(2):218–224. https://doi.org/10.1007/s00270-023-03649-x.	ASCENT predictor analysis rather than a comparison of cervical management nodes. Published outcomes are not stratified as A, B and C, and the reported predictors focus on post-stent medication and patency.
D00779. Romoli M, Molina C.A, de Sousa D.A, Arriaza E.Z, Jaikumar V, Jesser J, et al. EMERGENT CAROTID STENTING IN TANDEM OCCLUSIONS WITH EXRACRANIAL DISSECTION: THE MULTICENTER CERES-TANDEM STUDY. European Stroke Journal. 2026;11(Supplement 1):i1041. doi: 10.1093/esj/aakag023.1955.	Prespecified dissection subgroup entirely nested in the 4,053-patient CERES-TANDEM cohort. It does not separate balloon angioplasty from no acute cervical treatment and is not counted as an independent study alongside the main CERES paper; it provides subgroup context only.
D00019. Kaufmann R, van Dijk LC, Stomp W, de Laat KF, de Bruijn SFTM, Piet JR, et al. Emergency carotid artery stenting during endovascular treatment of tandem lesions in acute ischemic stroke: a single-center retrospective study. CVIR Endovasc. 2026;9(1):67. doi: 10.1186/s42155-026-00712-9.	Explicit single-arm eCAS cohort with no eligible A or B comparator.
D00020. Le TM, Tran CC, Nguyen GL, Nguyen NH, Nguyen TT, Tran LBM. Tandem occlusions after emergent stenting in acute ischemic stroke: A retrospective cohort study from a comprehensive stroke center's experience (TOESIS study). J Int Med Res. 2026;54(6):3000605261454076. doi: 10.1177/03000605261454076.	All 145 participants underwent emergent extracranial stenting; the comparison is between vascular territories rather than cervical management strategies.

Report citation	Reason for exclusion
D00060. Jabłoński B, Wyszomirski A, Pracoń A, Stańczak M, Gąsecki D, Gorycki T, et al. The Safety and Efficacy of Mechanical Thrombectomy with Acute Carotid Artery Stenting in an Extended Time Window: A Single-Center Study. Med Sci (Basel). 2026;14(1):47. doi: 10.3390/medsci14010047.	The eCAS arm is only 80% tandem lesions and the nonstenting comparator is a broad LVO thrombectomy cohort. Eligible tandem data are not separately reported for both arms.
D00023. Yi T, Lin D, Wu M, He Y, Gan S, Huang M, et al. Endovascular Management of Embolic Cervical Tandem Occlusion: 2-Center Technical Series. World Neurosurg. 2026;212:125020. doi: 10.1016/j.wneu.2026.125020.	Technical series of four cervical thrombus-management workflows, including cervical thrombectomy strategies not represented by protocol nodes A, B or C. It is not a clean comparison of the prespecified cervical-lesion treatments.
D00213. van Elk T, Maes L, van der Meij A, Lemmens R, Uyttenboogaart M, de Borst GJ, et al. Immediate Carotid Artery Stenting or Deferred Treatment in Patients With Tandem Carotid Lesions Treated Endovascularly for Acute Ischaemic Stroke. EJVES Vasc Forum. 2024;61:31-35. doi: 10.1016/j.ejvsvf.2023.12.002.	Commentary/editorial discussing the clinical dilemma and ongoing trials; it contains no original comparative patient cohort.
D00312. Yamashita S, Imahori T, Koyama J, Tanaka K, Okamura Y, Arai A, et al. Endovascular Treatment of Tandem Atherosclerotic Cervical Internal Carotid Artery Occlusion in the Setting of Acute Ischemic Stroke. Vasc Endovascular Surg. 2023;57(2):137-148. doi: 10.1177/15385744221130865.	Ten-patient technical case series with only nine successfully crossed lesions and no formal comparative effect estimate. Although PTA and stenting are described, the report is not an eligible comparative cohort.
D00331. Da Ros V, Pusceddu F, Lattanzi S, Scaggiante J, Sallustio F, Marrama F, et al. Endovascular treatment of patients with acute ischemic stroke and tandem occlusion due to internal carotid artery dissection: A multicenter experience. Neuroradiol J. 2023;36(1):86-93. doi: 10.1177/19714009221108673.	Management options (angioplasty, aspiration and stenting) are mentioned, but outcomes are analysed mainly by antegrade versus retrograde order and are not reported by the three cervical-treatment nodes.
D00286. Enriquez BAB, Nome T, Nome CG, Tennøe B, Lund CG, Beyer MK, et al. Predictors of outcome after endovascular treatment for tandem occlusions: a single center retrospective analysis. BMC Neurol. 2023;23(1):82. doi: 10.1186/s12883-023-03127-4.	The comparison is tandem versus single occlusion and predictor modelling. Eighty-one percent of tandem patients were stented, but outcomes are not stratified against a clearly defined A or B group.
D00170. Scarcia L, Colò F, Alexandre AM, Brunetti V, Pedicelli A, Arba F, et al. Effects of Emergent Carotid Stenting Performed before or after Mechanical Thrombectomy in the Endovascular Management of Patients with Tandem Lesions: A Multicenter Retrospective Matched Analysis. AJNR Am J Neuroradiol. 2025;46(1):96-101. doi: 10.3174/ajnr.a8421.	All eligible participants received node C; the study compares treatment order (stent-first versus thrombectomy-first), which is not a primary network node.
D00868. Mehta N, McDougall C. MINIMIZING RISK, MAXIMIZING PATENCY: SCORING BALLOON ANGIOPLASTY IN THE ACUTE MANAGEMENT OF TANDEM LESIONS. Journal of NeuroInterventional Surgery. 2025;17(Supplement 1):A159. https://jnis.bmj.com/content/17/Suppl_1/A159	Explicit case series in which all patients started with scoring-balloon angioplasty and six received rescue stents only after failure. Rescue-selected outcomes do not provide an unbiased comparative B-C cohort.
D00920. Maes, L, Van Elk, T, Van Der Meij, A, Nederkoorn, P, Lemmens, R, Uyttenboogaart, M. CAROTID ARTERY STENTING DURING ENDOVASCULAR TREATMENT OF ACUTE ISCHEMIC STROKE (CASES) - A MULTICENTER RANDOMIZED CLINICAL TRIAL. European stroke journal. 2024;9(1):568. doi: 10.1177/23969873241245672.	Ongoing randomized trial description with recruitment status only and no comparative outcome results.

Report citation	Reason for exclusion
D00822. Anonymous. Stent Implantation Versus Balloon Dilation for Acute Anterior Circulation Tandem Occlusion: A Multicenter, Prospective, Randomized, Open-label, Blinded End-point Trial. clinicaltrials. 2023. https://clinicaltrials.gov/search?term=%22Stent%20Implantation%20Versus%20Balloon%20Dilation%20for%20Acute%20Anterior%20Circulation%20Tandem%20Occlusion%22	Trial registry record describing an ongoing B-C randomized study; no results are reported. The record was not included in the present synthesis.

The table reproduces the 40 full-text exclusions in the finalized screening log. One recorded primary reason is shown per report.

L35 represents the Wu et al. article (DOI 10.16781/j.0258-879x.2022.01.0072). L34 was a duplicate database record and was removed from the included catalogue, without an additional full-text exclusion.

Table S5. Catalogue and characteristics of included and companion reports.

Report citation	Report family and analysis role	Setting, design and recruitment	Population and sample	Cervical strategy and outcomes
L32. Marko M, Cimflova P, Poppe AY, Kashani N, Singh N, Ospel J, et al. Management and outcome of patients with acute ischemic stroke and tandem carotid occlusion in the ESCAPE-NA1 trial. J Neurointerv Surg. 2022;14(5):429-433. doi: 10.1136/neurintsurg-2021-017474.	F01: ESCAPE-NA1 tandem-lesion subgroup Core non-CERES adjusted evidence (90-day function); four-node sensitivity analysis (90-day function and sICH)	International multicentre ESCAPE-NA1 trial subgroup; observational comparison of acute cervical stenting versus no stenting within a randomized nerinetide/placebo trial. The tandem substudy does not specify recruitment calendar dates in its patient-population methods.	Report total n=115	C/A+B mixed; C; A+B mixed Outcomes: 90-day mRS 0-2; sICH; 24-hour follow-up imaging; Mortality; 90 days; TICI 2b-3 reperfusion; end of procedure; Unlabelled adjusted estimate (endpoint not established; not pooled)
L33. Divani A, Farooqui M, Quispe-Orozco D, Ikram A, Anwar H, Castonguay A, et al. MANAGEMENT OF ACUTE ISCHEMIC STROKE PATIENTS WITH TANDEM LESIONS: ANALYSIS FROM THE PROXIMAL INTERNAL CAROTID ARTERY ACUTE STROKE SECONDARY TO TANDEM OCCLUSION (PICASSO) REGISTRY. European Stroke Journal. 2022;7(1 SUPPL):195.	F02: International Tandem Consortium/PICASSO family Selected family results: Farooqui 2023 [D00285], Tables S6b–S6c and S8a. No additional independent sample.	Multicentre registry analysis, 15 centres; conference abstract; recruitment dates NR.	PICASSO tandem-lesion population: 686 screened, 589 analysed; stenting 363 and non-stenting 225 (reported arm total, 588; one-patient discrepancy).	Acute stenting vs mixed non-stenting. Outcomes: 90-day mRS 0-2, sICH, reperfusion and mortality.

Report citation	Report family and analysis role	Setting, design and recruitment	Population and sample	Cervical strategy and outcomes
L37. Wahood W, Gholami M, Akhras A, Baghestani A, Brinjikji W. Abstract No. 233 Nationwide trends in utilization of carotid artery stenting and angioplasty in tandem occlusion stroke. J Vasc Interv Radiol. 2022; 33(6 Suppl):S107. doi:10.1016/j.jvir.2022.03.314.	F04: US National Inpatient Sample administrative cohort. Descriptive in-hospital evidence only; 90-day mRS 0-2 and sICH not reported in the abstract.	United States HCUP-NIS; retrospective administrative-database analysis with discharge weights; conference abstract. Recruitment is inconsistent in the source: Methods 2009-2018, Results/trend analysis 2006-2018.	Tandem ICA/MCA occlusion treated with thrombectomy and CAS or angioplasty. Reported group estimates: C=6,632 and B=3,357 (sum 9,989); national weighted estimates, not a verified unique-patient count.	B vs C. Inpatient mortality, non-routine discharge and temporal utilization trends; no 90-day mRS 0-2 or sICH counts.
L38. Garg A, Quispe Orozco D, Limaye K, Zevallos C, Mendez Ruiz A, Dajles A, et al. Abstract P553: Outcomes of Acute Endovascular Cervical Carotid Revascularization in Anterior Circulation Tandem Occlusions During Mechanical Thrombectomy. Stroke. 2021; 52(Suppl 1). doi:10.1161/str.52.suppl_1.P553.	F05: HCUP-NRD 2016-2017 family. Descriptive companion evidence; combined B+C revascularization cannot enter the A/B/C/D networks.	United States Nationwide Readmissions Database, 2016-2017; retrospective administrative cohort with propensity matching; conference abstract.	15,137 anterior-circulation LVO thrombectomy hospitalizations; 1,214 received CAS/angioplasty during the same hospitalization; matched analysis 1,063 pairs. Mean age 70.1±14.6 years; 51.1% female.	Combined CAS/angioplasty vs no cervical revascularization; timing is same hospitalization, not confirmed same procedure. Outcomes: in-hospital hemorrhagic transformation, mortality/discharge/resource use and 30-day readmission.
L39. Gigliotti MJ, Sweid A, El Naamani K, Patel N, Cockroft KM, Park C, et al. Management of Internal Carotid Artery and Intracranial Anterior Circulation Tandem Occlusion with Stenting versus No Stenting: A Multicenter Study. World Neurosurg. 2021;153:e237-e243. doi: 10.1016/j.wneu.2021.06.081.	F06: Salem multicentre cohort Strict network: 90-day function and sICH	United States; 2 centres; retrospective multicentre cohort with prospectively collected data; Jan 2012–May 2020	Report total n=92. Cervical aetiology: Mixed; dissection 5/92; exact atherosclerosis count unavailable	B: BA+MT; C: CAS+MT; sequence not reported Outcome sample sizes: mRS 0–2: B66/C26; sICH: B66/C26. Outcomes: 90-day mRS 0-2; sICH; 24-72 hours or index admission; Mortality; 90 days; TICI 2b-3 reperfusion; End of procedure
L40. Anadani M, Marnat G, Consoli A, Papanagiotou P, Nogueira RG, Siddiqui A, et al. Endovascular Therapy of Anterior Circulation Tandem Occlusions: Pooled Analysis From the TITAN and ETIS Registries. Stroke. 2021;52(10):3097-3105. doi: 10.1161/strokeaha.120.033032.	F07: TITAN/ETIS registry family Contextual pooled TITAN/ETIS report; not added independently to the strict network. Selected TITAN result: Papanagiotou 2018 [L55], Tables S6a and S7. Populations are not wholly identical.	Pooled French ETIS and international TITAN registries; TITAN January 2012-September 2016; ETIS January 2013-July 2019.	Anterior-circulation tandem occlusion treated with EVT; n=603 (stenting 341; non-stenting 262).	Acute stenting vs non-stenting. Outcomes: 90-day mRS 0-2 and 0-1, reperfusion, any ICH, sICH and parenchymal haematoma type 2.

Report citation	Report family and analysis role	Setting, design and recruitment	Population and sample	Cervical strategy and outcomes
L41. Garg A, Zevallos C, Quispe Orozco D, Mendez Ruiz A, Farooqui M, Zaidat O, et al. Emergent versus delayed carotid artery stenting following intracranial thrombectomy for acute stroke with tandem occlusion. Journal of NeuroInterventional Surgery. 2021;13(SUPPL 1):A18-A19. https://doi.org/10.1136/neurintsurg-2021-SNIS.27	F05: HCUP-NRD 2016–2017 family Related HCUP-NRD 2016–2017 emergent-versus-delayed CAS report. Administrative inpatient outcomes; not a separate independent co-primary comparison. No 90-day mRS 0–2 or clearly defined sICH result.	United States Nationwide Readmissions Database, 2016-2017; retrospective administrative-database study; conference abstract.	AIS hospitalizations with anterior-circulation EVT and CAS: n=1,601 (emergent 1,392; delayed 209).	Same-day CAS vs CAS on days 2-90. Outcomes: in-hospital mortality, ICH, discharge disposition, ventilation, gastrostomy, length of stay and charges.
L42. Garg, A, Farooqui, M, Limaye, K, Dajles, A, Ruiz, AM, Zevallos, C, et al. Emergent Carotid Artery Stenting and Intracranial Thrombectomy for Acute Stroke With Tandem Occlusion. STROKE. 2021;52(SUPPL 1):no pagination. doi: 10.1161/str.52.suppl_1.p546.	F05: HCUP-NRD 2016–2017 family Conference precursor to the HCUP-NRD B-versus-C publication by Garg 2021 [L44]; no separate independent contribution. Source outcomes are displayed under L44 in Table S8h.	United States Nationwide Readmissions Database, 2016-2017; retrospective administrative-database study; conference abstract.	AIS hospitalizations with same-day anterior-circulation EVT and CAS or angioplasty: n=2,042 (CAS 1,391; angioplasty 651).	C vs B. Outcomes: in-hospital ICH and mortality, ventilation, gastrostomy, length of stay, charges and 30-day readmission.
L43. Kalmar PJ, Tarkanyi G, Nagy CB, Csecsei P, Lenzser G, Bosnyak E, et al. Comparing Endovascular Treatment Methods in Acute Ischemic Stroke Due to Tandem Occlusion Focusing on Clinical Aspects. Life (Basel). 2021;11(5):458. doi: 10.3390/life11050458.	F08: Kalmar multicentre cohort Strict network: 90-day function and sICH	Hungary; 3 centres; retrospective analysis of a prospective registry; Nov 2017–Dec 2020	Report total n=101. Cervical aetiology: Atherothrombotic only; dissection excluded	B: BA→MT; C: CAS+BA→MT (generally antegrade) Outcome sample sizes: mRS 0–2: B29/C68; sICH: B29/C72. Outcomes: 90-day mRS 0-2; sICH; 24-72 hours or index admission; Mortality; 90 days; TICI 2b-3 reperfusion; End of procedure; Puncture to revascularization time; Procedure

Report citation	Report family and analysis role	Setting, design and recruitment	Population and sample	Cervical strategy and outcomes
L44. Garg A, Farooqui M, Zevallos CB, Quispe-Orozco D, Mendez-Ruiz A, Zaidat O, Ortega-Gutierrez S. Carotid artery angioplasty versus stenting for management of acute tandem occlusions. J Neurol Sci. 2021; 428:117588. doi:10.1016/j.jns.2021.117588. PMID:34403954.	F05: HCUP-NRD 2016–2017 family; preferred full publication over conference precursor L42. Descriptive inpatient/readmission evidence; no 90-day mRS or sICH contribution and no additional independent L42 sample.	United States Nationwide Readmissions Database, 2016–2017; retrospective survey-weighted administrative cohort. Logistic models for inpatient outcomes and Cox analysis for post-discharge readmission.	Reported national estimates: 2,042 hospitalizations, C=1,391 and B=651; median age 66 years (IQR 58–75), 31.3% female. Readmission analysis: 1,363 discharge survivors with known disposition (C=912, B=451), after specified exclusions. Unweighted/model-specific sample sizes are not reported.	Admission-day anterior-circulation EVT with carotid stenting (C) vs angioplasty without same-day stenting (B); detailed occlusion anatomy cannot be verified from codes. Outcomes: index-admission ICH/death composite and components, discharge home, other complications/resource use and 30-day readmission. No 90-day mRS or specifically adjudicated sICH. Source: full report pp2–5, Table 1/Figure 1 and supplement Tables S1–S3.
L45. Li W, Zhao C, Zhang M, Yang S, Zhao Z, Wang T, et al. Angioplasty alone versus acute stenting for acute tandem occlusions due to internal carotid artery atherosclerotic. Clin Neurol Neurosurg. 2021;208:106818. doi: 10.1016/j.clineuro.2021.106818.	F09: Li 2021 multicentre propensity cohort Strict network: 90-day function and sICH	China; 5 centres; retrospective analysis of prospective registries; Jan 2016–Jul 2019; propensity-matched analysis	Report total n=75; Analysis n=44. Cervical aetiology: Atherosclerotic only	B: BA→MT; C: BA→MT with acute CAS; uniform CAS timing not reported Outcome sample sizes: mRS 0–2: B22/C22; sICH: B22/C22. Outcomes: 90-day mRS 0-2; sICH; 24-72 hours or index admission; Mortality; 90 days; TICI 2b-3 reperfusion; End of procedure
L46. Marnat G, Lapergue B, Sibon I, Gariel F, Bourcier R, Kyheng M, et al. Safety and Outcome of Carotid Dissection Stenting During the Treatment of Tandem Occlusions: A Pooled Analysis of TITAN and ETIS. Stroke. 2020;51(12):3713-3718. doi: 10.1161/strokeaha.120.030038.	F07: TITAN/ETIS registry family Contextual dissection-restricted TITAN/ETIS report; not added independently to the selected TITAN comparison [L55], Tables S6a and S7.	24 comprehensive stroke centres; retrospective analysis of two prospective registries; January 2012-January 2019.	TITAN/ETIS cervical-dissection tandem occlusion: n=136 (stenting 65; non-stenting 71).	Acute stenting vs non-stenting. Outcomes: 90-day mRS 0-2, reperfusion, sICH and 90-day mortality.

Report citation	Report family and analysis role	Setting, design and recruitment	Population and sample	Cervical strategy and outcomes
L47. Kim B, Kim BM, Bang OY, Baek JH, Heo JH, Nam HS, et al. Carotid Artery Stenting and Intracranial Thrombectomy for Tandem Cervical and Intracranial Artery Occlusions. Neurosurgery. 2020;86(2):213-220. doi: 10.1093/neuros/nyz026.	F10: Kim multicentre cohort Strict network: 90-day function and sICH	Republic of Korea; 17 centres; retrospective analysis of prospective registries; Jan 2011–Dec 2015	Report total n=75. Cervical aetiology: Atherosclerosis 70/75; dissection 5/75	B: BA→MT; C: CAS→MT (50) or BA→MT→CAS (6) Outcome sample sizes: mRS 0–2: B19/C56; sICH: B19/C56. Outcomes: 90-day mRS 0-2; sICH; 24-72 hours or index admission; Mortality; 90 days; TICI 2b-3 reperfusion; End of procedure
L48. Jadhav AP, Zaidat OO, Liebeskind DS, Yavagal DR, Haussen DC, Hellinger FR Jr, et al. Emergent Management of Tandem Lesions in Acute Ischemic Stroke. Stroke. 2019;50(2):428-433. doi: 10.1161/strokeaha.118.021893.	F11: STRATIS registry tandem-lesion substudy Strict network: 90-day function and sICH	United States; 55 centres; prospective nonrandomized registry; Aug 2014–Jun 2016	Report total n=147; Analysis n=121. Cervical aetiology: Mixed/unspecified; counts not reported	B: angioplasty only; C: acute CAS; sequence counts unavailable Outcome sample sizes: mRS 0–2: B41/C73; sICH: B38/C69. Outcomes: 90-day mRS 0-2; sICH; 24-72 hours or index admission
L49. Dorado L, Lopez-Cancio E, Hernendez-Perez M, Gomis M, Ramos-Pachon A, Palomeras E, et al. Clinical outcome of acute ischemic stroke patients with arterial tandem occlusions by emergent cervical carotid approach of revascularization. European Stroke Journal. 2019;4(Supplement 1):122.	F12: Dorado single-centre family Related later report; no additional independent strict-network sample. Selected family result: Dorado 2017 [L62], Tables S6a and S7.	Retrospective review of a prospective EVT registry over 10 years; location and calendar dates NR; conference abstract.	641 anterior-circulation LVO cases; 107 tandem occlusions; 95 underwent intracranial thrombectomy (stenting 30; non-stenting 65).	Stenting vs angioplasty, aspiration or no cervical treatment. Outcomes: early neurological deterioration, 3-month mRS >2, ICA reocclusion and 24-hour sICH.
L50. Gomez-Escalonilla Escobar C.I, Rosati S, Calleja Castano P, Trillo Senin S, Ostoz Moliz F, Aguirre Hernandez C, et al. Carotid stenting or angioplasty in acute ischemic stroke due to tandem occlusion: Tattoo study. European Stroke Journal. 2019;4(Supplement 1):481. https://doi.org/10.1177/2396987319845581	F13: TATTOO multicentre cohort Descriptive report only: strategy frequencies are reported, but no A/B/C-specific co-primary outcome counts or cervical-strategy effect estimate is available in the conference abstract.	Spain; multicentre retrospective cohort; January 2014-December 2017; conference abstract AS07-038.	172 tandem-occlusion patients; median age 65 years, median NIHSS 17.	Cervical stenting 41.7%, angioplasty 29.2%, aspiration 7.7%, no cervical treatment 21.4%. Overall mRS ≤2 at 3 months 49.7%, sICH 7%, mortality at 3 months 8.1%, TICI 2b-3 77.3%. Outcomes are not cross-tabulated by cervical strategy. Reported adjusted effects concern procedural order and general anaesthesia, not the A/B/C cervical-strategy comparison.

Report citation	Report family and analysis role	Setting, design and recruitment	Population and sample	Cervical strategy and outcomes
L52. Paipa Merchan AJ, Lara Rodriguez B, Quesada Garcia H, Aja Rodriguez L, Mora Montoya P, Chirife Chaparro O, et al. Tandem occlusions and endovascular treatment. Eur Stroke J. 2018; 3(1 Suppl):195. Abstract AS07-089; supplement doi:10.1177/2396987318770127.	F14: Bellvitge cohort. Descriptive secondary-outcome evidence with extractable reperfusion counts; no arm-specific 90-day function or sICH data.	Hospital Universitari de Bellvitge, Barcelona, Spain; single-centre cohort review, June 2010-October 2017; conference abstract.	120 tandem occlusions among 822 thrombectomy patients; A=80, B=22, C=18. Critical cervical carotid stenosis/occlusion plus acute intracranial occlusion.	A/B/C. Intracranial TICI 2b-3 counts A=53/80, B=18/22, C=13/18. Three-month mRS 0-2 and sICH are discussed without arm-specific counts.
L53. Li W, Chen Z, Dai Z, Liu R, Yin Q, Wang H, et al. Management of acute tandem occlusions: Stent-retriever thrombectomy with emergency stenting or angioplasty. J Int Med Res. 2018;46(7):2578-2586. doi: 10.1177/0300060518765310.	F15: Li 2018 single-centre cohort Strict network: 90-day function and sICH	China; 1 centre; prospective registry cohort; Jan 2013–Jun 2016	Report total n=37. Cervical aetiology: Atherosclerosis 22/37; dissection 15/37	B: BA→MT; C: CAS→MT Outcome sample sizes: mRS 0–2: B18/C19; sICH: B18/C19. Outcomes: 90-day mRS 0-2; sICH; 24-72 hours or index admission
L54. Ramakrishnan P, Holston K, Leesch W, Sanderson J, Gossage M, Kostov D. E-160 Is acute angioplasty and carotid artery stenting in tandem occlusion patients feasible, safe, and effective? A single center experience. J Neurointerv Surg. 2018; 10(Suppl 2):A128. doi:10.1136/neurintsurg-2018-SNIS.236.	F16: Ramakrishnan single-centre cohort. Descriptive reperfusion and mRS 0-1 evidence; no extractable mRS 0-2 or sICH data.	Riverside Regional Medical Center, United States; retrospective single-centre series, July 2012-December 2017; conference abstract.	9 tandem-occlusion patients treated with intracranial mechanical thrombectomy; B=3, C=6.	Angioplasty alone vs angioplasty plus acute CAS. TICI 2b-3: 67% vs 100%; 90-day mRS ≤1: 0/3 vs 3/6. Discharge NIHSS/destination also collected; sICH counts not reported.
L55. Papanagiotou, P, Haussen, D.C, Turjman, F, Labreuche, J, Piotin, M, Kastrup, A, et al. Carotid Stenting With Antithrombotic Agents and Intracranial Thrombectomy Leads to the Highest Recanalization Rate in Patients With Acute Stroke With Tandem Lesions. JACC Cardiovasc Interv. 2018;11(13):1290-1299. doi: 10.1016/j.jcin.2018.05.036.	F07: TITAN/ETIS registry family Strict network: 90-day function and sICH	7 countries; 18 centres; retrospective pooled registry; recruitment dates not reported	Report total n=482. Cervical aetiology: Atherosclerosis 329/482; dissection 105/482; cardioembolic 48/482	A: MT only; B: angioplasty; C: acute CAS; sequence not reported Outcome sample sizes: mRS 0–2: A108/B52/C322; sICH: A108/B52/C322. Outcomes: 90-day mRS 0-2; sICH; 24-72 hours or index admission
L56. Weill A, Poppe A.Y, Chen B.Y, Keezer M.R, Ziegler D, Odier C, et al. Acute cervical carotid stenting in patients with tandem occlusions: a retrospective single-centre study. European stroke journal. 2018;3(1S):172. doi: 10.1177/2396987318770127.	F17: Weill/CHUM single-centre family Companion to L57/L63 in the same CHUM 73-patient cohort; no independent contribution. Available family outcome percentages are displayed once in Table S8h.	CHUM Notre-Dame Hospital, Montreal, Canada; retrospective single-centre study; December 2014-December 2016; conference abstract.	Tandem occlusion treated with EVT; n=73 (stenting 26; non-stenting 47).	Acute stenting vs mixed non-stenting. Outcomes: 90-day mRS 0-2, sICH, intracranial and ICA recanalization.

Report citation	Report family and analysis role	Setting, design and recruitment	Population and sample	Cervical strategy and outcomes
L57. Weill A, Poppe A, Chen BY, Keezer MR, Ziegler D, Odier C, et al. Acute cervical carotid stenting in patients with tandem occlusions: a retrospective single-centre study. Int J Stroke. 2018; 13(2 Suppl):124. Supplement doi:10.1177/1747493018789543.	F17: Weill/CHUM 73-patient family. Mixed-comparator narrative evidence; same cohort as L56 and L63, counted once and not independently pooled.	CHUM Notre-Dame Hospital, Montreal, Canada; retrospective single-centre study, December 2014-December 2016; conference abstract.	73 tandem-occlusion EVT patients: acute stenting 26, no stenting 47; severe cervical ICA stenosis/occlusion with distal ICA and/or MCA occlusion.	C vs source-defined non-stenting (A/B not separated). Reported C/non-stenting: 90-day mRS 0-2 46%/30%; sICH 8%/4%; TICI 2b-3 77%/79%; ICA recanalization 92%/9%.
L58. Poppe A, Daneault N, Deschaintre Y, Gioia L, Jacquin G, Nico L, et al. A pilot randomized sub-study of patients with tandem carotid occlusion undergoing endovascular thrombectomy for anterior circulation stroke in the endovascular acute stroke intervention (EASI) trial. European stroke journal. 2018;3(1S):157. doi: 10.1177/2396987318770127.	F18: EASI randomized pilot family Interim EASI report; no additional independent sample. Completed pilot: Poppe 2020 [D00540], RoB 2 in Table S3 and results in Table S8b.	CHUM, Montreal, Canada; single-centre randomized EASI substudy, 2013-2015; interim conference abstract.	16 tandem-lesion patients among 138 EASI participants; acute stenting 9 and no stenting 7.	Acute stenting vs no acute stenting; 90-day mRS 0-2 and mRS 5-6. Companion of the completed 24-patient randomized pilot.
L59. Rodriguez-Villatoro N, Rodriguez-Luna D, Ribo M, Muchada M, Requena M, Pagola J, et al. Abstract WMP40: A Comparison of Three Different Endovascular Approaches for Extracranial Internal Carotid Artery Lesions in the Setting of Tandem Occlusions. Stroke. 2018; 49(Suppl 1). doi:10.1161/str.49.suppl_1.WMP40.	F19: Vall d'Hebron prospective cohort. Descriptive report; co-primary outcome counts/effect estimates are unavailable.	Vall d'Hebron University Hospital, Barcelona, Spain; prospective consecutive cohort, April 2013-April 2017; conference abstract.	92 non-cardioembolic tandem-occlusion patients treated with EVT within 6 hours: B=40, C=52; age 64.6±12.6 years; IV rtPA 50/92.	Angioplasty alone vs acute stenting (self-expanding or balloon-expanding, both C). Outcomes: 24-hour ICA restenosis/occlusion and NIHSS change, discharge NIHSS, 3-month mRS and stroke recurrence, HT. No usable group mRS/sICH estimates.
L62. Dorado L, Lopez-Cancio E, Hernandez-Perez M, Gomis M, Perez De La Ossa N, Palomeras E, et al. Clinical outcome of acute ischemic stroke patients with emergent arterial tandem occlusions by cervical carotid type of revascularization. European Stroke Journal. 2017;2(1 Supplement 1):85.	F12: Dorado single-centre family Strict network: 90-day function and sICH	Country not reported in abstract; 1 centre; prospective registry; Mar 2008–Apr 2016; conference abstract	Report total n=68. Cervical aetiology: Cervical ICA plus MCA occlusion; aetiology not reported	B: isolated BA+MT; C: CAS±BA+MT; sequence not reported Outcome sample sizes: mRS 0–2: B32/C25; sICH: B40/C28. Outcomes: 90-day mRS 0-2; sICH; 24-72 hours or index admission
L63. Weill A, Poppe AY, Chen BY, Deschaintre Y, Jacquin G, Stapf C. Acute cervical carotid stenting versus delayed intervention in patients with tandem occlusions undergoing endovascular therapy for anterior circulation stroke: a retrospective single-centre case-series. Int J Stroke. 2017; 12(Suppl):39.	F17: Weill/CHUM 73-patient family. Earlier overlapping conference report of L56/L57; narrative companion, not independently pooled.	CHUM, Montreal, Canada; retrospective single-centre EVT cohort, 2014-2016; conference abstract; later companion specifies December 2014-December 2016.	73 tandem-occlusion EVT patients: acute stenting 26 and no stenting/deferred treatment 47; same sample and outcomes as L56/L57.	C vs non-stenting/deferred cervical intervention; A/B not separated. Same reported percentages as L57 for 90-day mRS 0-2, sICH and intracranial/ICA recanalization.

Report citation	Report family and analysis role	Setting, design and recruitment	Population and sample	Cervical strategy and outcomes
L65. Motta C, Pizzuto S, Merolla S, Gandini R, Stanzione P, Koch G, Sallustio F. Acute extracranial stenting is associated with favorable outcome in atherosclerotic patients with tandem occlusion of anterior circulation treated with mechanical thrombectomy. Cerebrovasc Dis. 2016; 41(Suppl 1):8.	F20: Motta single-centre cohort. Mixed-comparator narrative evidence; no numerical eligible adjusted effect or arm-specific co-primary data.	Single stroke centre, location not specified in the indexed abstract; retrospective cohort, 2012-2015; conference abstract.	60 acute ipsilateral ICA/MCA tandem occlusions; CAS 32, non-CAS 28. Atherothrombotic 51.7%, cardioembolic 30%, dissection 5%, undetermined 13.3%.	C vs incompletely specified non-CAS cervical management. Three-month mRS analyzed by aetiology; TICI and sICH used as regression covariates. No arm mRS 0-2/sICH counts or adjusted OR/CI reported.
F15. Marnat G, Mourand I, Eker O, Machi P, Arquizan C, Riquelme C, et al. Endovascular Management of Tandem Occlusion Stroke Related to Internal Carotid Artery Dissection Using a Distal to Proximal Approach: Insight from the RECOST Study. AJNR Am J Neuroradiol. 2016;37(7):1281-8. doi: 10.3174/ajnr.a4752.	F21: RECOST dissection tandem subgroup Strict network: 90-day function and sICH	France; 1 centre; retrospective treatment-subgroup analysis of a prospective database; Aug 2009–Apr 2013	Report total n=20. Cervical aetiology: Dissection only (20/20)	A: MT only; C: MT→selective CAS when collaterals were inadequate Outcome sample sizes: mRS 0–2: A15/C5; sICH: A15/C5. Outcomes: 90-day mRS 0-2; sICH; 24-72 hours or index admission
F16. Fahed R, Redjem H, Blanc R, Labreuche J, Robert T, Ciccio G, et al. Endovascular Management of Acute Ischemic Strokes with Tandem Occlusions. Cerebrovasc Dis. 2016;41(5-6):298-305. doi: 10.1159/000444068.	F22: Fahed single-centre registry. Descriptive source evidence only. Centre, authors and recruitment periods overlap TITAN, which is retained in the pooled analyses. The EVT-start cohort includes aborted procedures; no completed-thrombectomy-only outcomes, A/B-separated comparison or sICH result are available.	Fondation Ophtalmologique Adolphe de Rothschild, Paris, France; one treating centre. Retrospective analysis of a prospectively gathered consecutive registry, November 2011–August 2014.	70 tandem-occlusion patients starting an EVT attempt: C=37, D=33; mean age 62.6±11.3 years, 49/70 male; atheroma 38/70, dissection 13/70, cardioembolism 13/70. Ten procedures were stopped during/after initial angiography and remain in the cohort. Three-month follow-up: C=36, D=31.	C: acute ICA stenting; D: no acute stent, with A/B composition not separable. Three-month mRS 0–2 and mortality, procedural TICI 2b–3, and HT at 24 h/within 72 h have arm counts (Table 3). HT is not sICH. No stent-adjusted OR. Source: full report pp299–304, especially Table 3 (p302).
D00540. Poppe AY, Jacquin G, Stapf C, Daneault N, Deschaintre Y, Gioia LC, et al. A randomized pilot study of patients with tandem carotid lesions undergoing thrombectomy. J Neuroradiol. 2020;47(6):416-420. doi: 10.1016/j.neurad.2019.08.003.	F18: EASI randomized pilot family Randomized pilot, reported separately; four-node sensitivity analysis (90-day function and sICH)	CHUM, Montreal, Canada; single-centre randomized EASI pilot; April 2013-December 2018; 24 tandem-lesion patients randomized 1:1 to acute stenting or no acute stenting.	Report total n=24	C/D; A; C Outcomes: 90-day mRS 0-2; sICH; 24-hour brain imaging (ECASS-2)

Report citation	Report family and analysis role	Setting, design and recruitment	Population and sample	Cervical strategy and outcomes
D00445. Nolan NM, Regenhardt RW, Koch MJ, Raymond SB, Stapleton CJ, Rabinov JD, et al. Treatment Approaches and Outcomes for Acute Anterior Circulation Stroke Patients with Tandem Lesions. J Stroke Cerebrovasc Dis. 2021;30(2):105478. doi: 10.1016/j.jstrokecerebrovasdis.2020.105478.	F23: Massachusetts General Hospital tandem cohort. Source-reported C-versus-D functional, symptomatic-haemorrhage and reperfusion effects are contextual only. Arm counts and model covariates are not reported; the effects cannot enter the raw-count network or be classified as documented baseline-adjusted evidence.	USA; Massachusetts General Hospital, Boston; 1 centre; retrospective analysis of a prospectively maintained database; January 2011–September 2019.	62 tandem-lesion patients; 60 in treatment/outcome analyses after exclusion of two Merci-treated patients; 55 had accessible cervical ICAs (C34/A13/B8), with five inaccessible ICAs excluded from treatment-association analyses. Cervical aetiology: atherosclerosis 46/62, dissection 16/62. Follow-up mRS available for 55/62 overall; outcome-specific n for Table 3 is not given.	A: no acute cervical treatment, n=13; B: angioplasty alone, n=8; C: acute stenting, n=34; stenting before or immediately after intracranial EVT. Table 3 compares C34 with pooled A+B21. Outcomes: 90-day mRS 0–2; symptomatic PH1/PH2 during admission (ECASS); TICI 2b–3; overall 90-day mortality; stent patency at median 7 days.
D00589. Eker OF, Bühlmann M, Dargazanli C, Kaesmacher J, Mourand I, Gralla J, et al. Endovascular Treatment of Atherosclerotic Tandem Occlusions in Anterior Circulation Stroke: Technical Aspects and Complications Compared to Isolated Intracranial Occlusions. Front Neurol. 2018;9:1046. doi: 10.3389/fneur.2018.01046.	F24: Bern+Montpellier atherosclerotic tandem cohort Strict network: 90-day function and sICH	Switzerland/France; 2 centres; retrospective analysis of prospective databases; Jan 2010–Sep 2015	Report total n=121. Cervical aetiology: Atherosclerotic only (121/121)	A: MT with cervical stenting deferred 2–5 d; B: acute PTA; C: acute CAS Outcome sample sizes: mRS 0–2: A14/B8/C96; sICH: A14/B9/C98. Outcomes: 90-day mRS 0-2; sICH; 24-72 hours or index admission
D00577. Kang DH, Kim YW, Hwang YH, Kim YS. Endovascular Recanalization of Acute Tandem Cervical Carotid and Intracranial Occlusions: Efficacy of Cervical Balloon Angioplasty Alone Then Intracranial Target Recanalization Strategy. World Neurosurg. 2019;126:e1268-e1275. doi: 10.1016/j.wneu.2019.02.240.	F25: Kang single-centre cohort Strict network: 90-day function and sICH	Republic of Korea; 1 centre; retrospective analysis of a prospective registry; Apr 2009–Mar 2017	Report total n=62. Cervical aetiology: Atherosclerotic only (62/62)	B: BA→MT; C: BA→MT→rescue CAS (31) or BA→CAS→MT (9) Outcome sample sizes: mRS 0–2: B22/C40; sICH: B22/C40. Outcomes: 90-day mRS 0-2; sICH; 24-72 hours or index admission
D00580. Wallocha M, Chapot R, Nordmeyer H, Fiehler J, Weber R, Stracke CP. Treatment Methods and Early Neurologic Improvement After Endovascular Treatment of Tandem Occlusions in Acute Ischemic Stroke. Front Neurol. 2019;10(FEB):127. doi: 10.3389/fneur.2019.00127.	F26: Wallocha single-centre cohort Strict network: sICH	Germany; 1 centre; retrospective analysis of a prospective database; Apr 2009–May 2016	Report total n=163. Cervical aetiology: Atherosclerotic only (163/163)	B: PTA+MT; C: stent-PTA+MT; mixed antegrade/retrograde order Outcome sample sizes: mRS 0–2: Not available; sICH: B14/C149. Outcomes: sICH; 24-72 hours or index admission

Report citation	Report family and analysis role	Setting, design and recruitment	Population and sample	Cervical strategy and outcomes
D01132. Troja W, Weyhenmeyer J, Villelli N.W, Bohnstedt B. Acute stenting of persistently stenotic cervical lesions in atherosclerotic tandem occlusions does not improve neurologic outcomes. Circulation. 2021; 144(SUPPL 1):no pagination. https://doi.org/10.1161/circ.144.suppl_1.12852	F27: Indiana University atherosclerotic tandem cohort Descriptive mixed/unclear nonstent comparator; no effect estimate or arm-specific outcome counts reported.	USA; Indiana University, Indianapolis; 1 centre; retrospective consecutive-thrombectomy cohort; 2011–2019; conference abstract 12852.	69 patients with atherosclerotic tandem occlusion and severe cervical stenosis ≥70%; acute stent n=35; no stent with persistent severe stenosis n=34.	C35 vs nonstent 34; prior balloon angioplasty in the nonstent group is not specified. Outcomes: carotid re-occlusion and neurological outcome/mRS at 90 days after discharge; reperfusion and length of stay. Only between-group p values are supplied (re-occlusion 0.434; neurological outcome 0.82); no events or CIs.
D00305. Yu M, Ma Y, Ren H, Huang Y, Yin L, Ma L, et al. Emergent Management of Tandem Occlusions in Anterior Circulation Stroke: A Single-Centre Experience. World Neurosurg. 2023;170:e491-e499. doi: 10.1016/j.wneu.2022.11.040.	F28: single-centre cohort Strict network: 90-day function and sICH	China; 1 centre; retrospective analysis of a prospective registry; Jan 2018–Dec 2021	Report total n=98. Cervical aetiology: Atherosclerotic only (98/98)	B: BA→MT; C: BA→MT→CAS after observation Outcome sample sizes: mRS 0–2: B34/C64; sICH: B34/C64. Outcomes: 90-day mRS 0-2; sICH; 24-72 hours or index admission; Mortality; 90 days; TICI 2b-3 reperfusion; End of procedure
D00283. Kojima T, Aihara M, Naito I, Shimizu T, Miyamoto N, Yamaguchi R, et al. Management of Cervical Internal Carotid Artery Stenosis or Occlusion in Tandem Lesion with Acute Ischemic Stroke: Which Is More Appropriate Percutaneous Transluminal Angioplasty or Stent?. World Neurosurg. 2023;175:e208-e217. doi: 10.1016/j.wneu.2023.03.054.	F29: Kojima-Takasaki_2014-2022 Four-node sensitivity analysis (sICH only)	Japan; retrospective series at Gunma University and affiliated hospitals; January 2014-June 2022; 50 tandem-lesion EVT patients. Dissection cases were excluded.	Report total n=50	C: acute carotid stenting; D: mixed nonstenting management (12 angioplasty-only and 2 untreated cases). Outcomes: sICH within 14 days after the procedure.
D00285. Farooqui M, Zaidat OO, Hassan AE, Quispe-Orozco D, Petersen N, Divani AA, et al. Functional and Safety Outcomes of Carotid Artery Stenting and Mechanical Thrombectomy for Large Vessel Occlusion Ischemic Stroke With Tandem Lesions. JAMA Netw Open. 2023;6(3):e230736. doi: 10.1001/jamanetworkopen.2023.0736.	F02: International Tandem Consortium/PICASSO family Sensitivity only: preferred PSM adjusts for periprocedural antiplatelets (90-day function; sICH)	International Tandem Consortium; 17 centres; retrospective international multicentre cross-sectional cohort; January 2015-December 2020.	Report total n=623	C/D; A+B mixed; C Outcomes: 90-day mRS 0-2; sICH; 24-72 hours or index admission; Mortality; 90 days; TICI 2b-3 reperfusion; end of procedure; sICH; Post-treatment safety window (study-defined); TICI 2b-3 reperfusion; End of procedure; discharge mRS 0–2; as labelled

Report citation	Report family and analysis role	Setting, design and recruitment	Population and sample	Cervical strategy and outcomes
D00320. Sallustio, F, Pracucci, G, Cappellari, M, Saia, V, Mascolo, A.P, Marrama, F, et al. Carotid artery stenting during endovascular thrombectomy for acute ischemic stroke with tandem occlusion: the Italian Registry of Endovascular Treatment in Acute Stroke. Acta Neurol Belg. 2023;123(2):475-485. doi: 10.1007/s13760-022-02067-z.	F30: IRETAS national registry. Descriptive source evidence only; TITAN is retained in the pooled analyses because shared centres and recruitment periods indicate possible partial overlap. Outcome-specific missingness and source conflicts are retained in Table S8h. Procedure-time-adjusted functional models are contextual, not primary total-effect estimates.	Italy; 56-centre national registry; retrospective analysis of consecutive EVT cases treated within 6 hours; January 2011-December 2017.	466 tandem-occlusion cases screened; 118 excluded for missing acute-CAS status; 348 analysed (C122; D226). Outcome-specific N: mRS 0-2 and mortality C118/D211; sICH C116/D217.	Acute CAS (C) versus no acute CAS (D). D includes carotid angioplasty in 23/226 and is neither pure A nor pure B. At 3 months: mRS 0-2 C54/118 versus D77/211; mortality C14/118 versus D50/211. sICH within 24 hours: C6/116 versus D5/217. Denominators use explicitly reported missing counts. Source Table 1 prints C54 (36.5%) for favourable outcome, inconsistent with C118 evaluable patients; this discrepancy is retained.
D00989. Madden S, Ford M, Atkinson T, Mcguire K, Wiggam I, Flynn P, et al. TANDEM OCCLUSIONS - TO STENT OR NOT TO STENT? A NORTHERN IRISH OBSERVATIONAL STUDY. European Stroke Journal. 2023;8(2 Supplement):227. https://academic.oup.com/esj/article/8/2_suppl/3/8269093	F31: Northern-Ireland-national-thrombectomy-centre_2014-2021 Contextual adjusted evidence (90-day function)	Royal Victoria Hospital, Belfast, Northern Ireland, United Kingdom; observational analysis of prospectively recorded patients at a national thrombectomy centre; 2014-2021; conference abstract 567.	Report total n=96	C versus D (source-defined nonstenting comparator; A/B not separated) Outcomes: 90-day mRS 0-2
D01000. Essibayi M.A, Anadani M, Almallouhi E, Yaghi S, Maier I, Jabbour P.M, et al. Acute Carotid Stenting Versus Conservative Management For Tandem Carotid Occlusions: Insights From STAR. Stroke. 2023;54(Supplement 1):no pagination. doi: 10.1161/str.54.suppl_1.WP164.	F32: STAR family Descriptive A–C results; outcome-specific denominators unavailable. Companion D00943 provides the B–C report; the potentially shared C arm is not counted twice as independent evidence.	Multicentre Stroke Thrombectomy and Aneurysm Registry; retrospective registry analysis; 2010–2022; number of recruiting centres/countries not stated in the abstract; conference abstract WP164.	688 tandem-carotid cases among 9812 thrombectomies; reported A–C comparison comprises A444/C132 (576 patients). A propensity-matched analysis includes 129 per group. D00943 separately reports B112/C132 (244 patients). Shared-arm membership is strongly suggested by identical registry and C132, but patient-level identity is not independently verified.	A: MT alone, n=444; C: emergent cervical stenting, n=132. Ninety-day mRS 0–2, mortality, sICH and mTICI 2b–3 reported as percentages without outcome-specific n. No cervical-treatment adjusted OR/CI is supplied in this abstract.

Report citation	Report family and analysis role	Setting, design and recruitment	Population and sample	Cervical strategy and outcomes
D00935. Bajrami A, Senadim S, Bas D, Geyik S. Comparison of PTA vs Bridging Theraphy with Balloon Expandable Stenting vs Self Expandable Carotid Stents for Management of Tandem Occlusions for acute Ischemic stroke. Interventional Neuroradiology. 2024;30(1 Supplement 1):181. https://doi.org/10.1177/15910199241285197	F33: Istanbul Aydin University cohort Descriptive three-node evidence; strategy-specific outcome events or effects are not published in the abstract.	Turkey; Istanbul Aydin University; 1 centre; retrospective study of a prospectively maintained database; recruitment dates not reported; conference abstract 1414.	202 consecutive patients; 147 men; mean age 66.07 (SD 11.21) years. A13; B47; C142, comprising balloon-expandable stents 43 and self-expandable stents 99. A13 includes cardioembolic/dissection cases.	A: MT with no additional carotid procedure; B: balloon angioplasty; C: balloon-expandable or self-expandable stenting. Outcomes: mRS 0–2 at 3 months, sICH on 24-h imaging and ICA patency. Stent-subtype outcome counts are not supplied; C outcome totals cannot be calculated.
D00943. Essibayi M, Almallouhi E, Anadani M, Medeiros R, Yaghi S, Maier I, et al. CAROTID ARTERY STENTING VERSUS BALLOON ANGIOPLASTY FOR MANAGEMENT OF ANTERIOR CIRCULATION TANDEM LARGE VESSEL OCCLUSIONS: A MULTICENTER STUDY. Journal of NeuroInterventional Surgery. 2024;16(Supplement 1):A51. doi: 10.1136/jnis-2024-snis.68.	F32: STAR family Contextual B–C adjusted average treatment effects; companion to D01000, with likely shared C132. Published ATEs are not odds ratios and treatment coding is not explicit.	Multicentre Stroke Thrombectomy and Aneurysm Registry; retrospective analysis with inverse-propensity scoring and regression adjustment (IPSWR); recruiting countries/centre count and dates not stated in this abstract. D01000 reports STAR 2010–2022.	244 tandem-occlusion patients: C132 (CAS plus BA) and B112 (BA alone). This report's n is 244, not the 688 tandem cases cited in companion D01000.	B: BA alone, n=112; C: BA plus CAS, n=132. Ninety-day good functional outcome; mortality; sICH; successful reperfusion. IPSWR ATEs and CIs reported for function, mortality and sICH; binary arm events/denominators absent.
D01439. Abousleiman, JM, Cognard, C, Bonneville, F, Marnat, G, Januel, AC, Tall, P, et al. Balloon angioplasty alone in carotid artery dissections: an overlooked therapeutic choice in acute ischemic stroke. JOURNAL OF NEURORADIOLOGY. 2025;52(5). doi: 10.1016/j.neurad.2025.101367.	F34: ETIS-France dissection report Contextual report only: the eligible tandem subgroup is identifiable, but its outcomes are not separable from isolated extracranial ICA disease in the published B-versus-C outcome tables.	France; ETIS registry, 33 centres; retrospective analysis; January 2015-January 2023.	160 dissection-related EVT cases (B26/C134), including 119 tandem occlusions (B19/C100) and 41 isolated extracranial ICA cases (B7/C34). The 119 is a subgroup count, not an analysed outcome denominator.	Balloon angioplasty without subsequent stenting (B) versus acute stenting (C). Published 3-month mRS, mortality and 24-hour sICH compare the full mixed 160-patient population; no tandem-only outcome counts are supplied. Full-population results are not used as outcomes for the 119-patient tandem subgroup.

Report citation	Report family and analysis role	Setting, design and recruitment	Population and sample	Cervical strategy and outcomes
D00199. Simon SR, Knapen RRMM, Truijman MTB, van Oostenbrugge RJ, Wagemans BAJM, van Zwam WH, et al. Timing of acute carotid artery stenting for tandem lesions in patients with acute ischemic stroke: A Maastricht Stroke Quality Registry (MaSQ-Registry) study. Interv Neuroradiol. 2024;32(4):1365-1372. doi: 10.1177/15910199241245166.	F35: Maastricht Stroke Quality Registry Procedural-order context only: the analysed comparison is acute stenting before versus after intracranial thrombectomy (C versus C), not different cervical-strategy nodes.	Netherlands; Maastricht tertiary centre; retrospective analysis of prospectively registered consecutive patients; September 2020-January 2023.	505 EVT patients screened; 80 had tandem lesions (14 no acute cervical treatment, 16 angioplasty only, 50 acute stenting). Only the 50 stented patients were analysed: stenting-before 31 versus stenting-after 19; 3-month mRS evaluable in 27 versus 19.	C-versus-C procedural sequence comparison. 3-month mRS 0-2 13/27 versus 9/19; ordinal mRS cOR 0.81 (0.29-2.30). No comparative A/B/C outcomes are reported for the 30 non-stented patients. Post-EVT intracranial haemorrhage is reported, not an eligible A/B/C sICH comparison.
D00893. Kaba E, Beyazal M. CAROTID STENTING VS BALLOON ANGIOPLASTY IN ACUTE ISCHEMIC STROKE WITH TANDEM OCCLUSION: A RETROSPECTIVE ANALYSIS OF SINGLE-CENTER EXPERIENCE. Neuroradiology. 2025;67(Supplement_1):S90-S91.	F36: Kaba–Beyazal single-centre cohort Secondary-outcome evidence only: discharge function, 24-h haemorrhagic transformation and reperfusion. No 90-day mRS or sICH endpoint is reported.	Turkey; Recep Tayyip Erdogan University, Rize; 1 centre; retrospective cohort; recruitment dates not reported; conference abstract.	20 tandem ICA/intracranial-occlusion patients; B13/C7; 15 men/5 women; mean age 69.15 years; IV tPA 17/20; reported admission NIHSS 16.9 and mean ASPECTS 8.5.	B: emergent BA alone, n=13; C: emergent carotid stenting, n=7; intracranial aspiration thrombectomy in both. Outcomes: TICI 2b–3 at procedure end; haemorrhagic transformation at 24h; mRS at discharge. Reperfusion succeeded in all 20. Haemorrhage43% in C vs 46% in B; discharge outcomes reported for the whole cohort only.
D00869. Florez N.R, Martinez A.S, Sierra M.R, Soto R.E.C, Diaz N.L, Perendreu J. OPTIMIZING STROKE MANAGEMENT ANGIOPLASTY WITH OR WITHOUT ICA STENTING IN TANDEM STROKE: A SINGLE-CENTER EXPERIENCE. Journal of NeuroInterventional Surgery. 2025;17(Supplement 2):A113. doi: 10.1136/jnis-2025-esmint.171.	F37: Parc Taulí2021–2025 cohort Descriptive B–C evidence; abstract reports arm sizes but no numerical clinical/procedural outcomes.	Spain; Parc Taulí Hospital, Sabadell; 1 centre; retrospective observational study; 2021–2025; conference abstract A205.	39 patients with tandem intracranial/cervical ICA occlusion; B13/C26.	B: BA alone, n=13; C: acute ICA stenting plus MT, n=26. Ninety-day mRS and periprocedural complications were assessed, but the abstract gives no outcome counts, percentages or effect CIs.
D01509. Faybushevich, A.G, Galustyan, H.V, Sysoev, V.M, Gitelzon, E.A, Chernyaev, M.V. Endovascular Treatment of Patients with Acute Ischemic Stroke Caused by Tandem Lesions of the internal Carotid Artery. Russian Sklifosovsky Journal of Emergency Medical Care. 2025;14(1):89-95. doi: 10.23934/2223-9022-2025-14-1-89-95.	F38: Moscow-Yudin cohort Strict network: 90-day function and sICH	Russian Federation; 1 centre; comparative study; allocation described as random but methods and recruitment period not reported	Report total n=94. Cervical aetiology: Atherosclerotic only (94/94)	A: cervical crossing±BA→MT, no acute revascularization; C: MT→CAS Outcome sample sizes: mRS 0–2: A46/C48; sICH: A46/C48. Outcomes: 90-day mRS 0-2; sICH; 24-72 hours or index admission; Mortality; 90 days; TICI 2b-3 reperfusion; End of procedure

Report citation	Report family and analysis role	Setting, design and recruitment	Population and sample	Cervical strategy and outcomes
D00124. Özdemir HN, Dere B, Güler A, Çınar C, Şirin H, Karaman B, et al. Stenting Versus Non-stenting Strategy in Endovascular Treatment of Acute Anterior Ischemic Stroke Patients with Tandem Occlusion. Ann Indian Acad Neurol. 2025;28(2):234-240. doi: 10.4103/aian.aian_828_24.	F39: Ege-University_2016-2024 Strict network: 90-day function	Turkey; 1 centre; retrospective historical-control cohort from a prospective database; Jan 2016–Jan 2024	Report total n=54. Cervical aetiology: Atherosclerosis 49/54; dissection 5/54	A: MT without acute CAS; C: MT→CAS+BA (retrograde) Outcome sample sizes: mRS 0–2: A19/C35; sICH: Not available. Outcomes: 90-day mRS 0-2; Mortality; 90 days; TICI 2b-3 reperfusion; End of procedure
D00141. Sousa JA, Rodrigo-Gisbert M, Shu L, Luo A, Xiao H, Mahmoud NA, et al. Emergent Carotid Stenting During Thrombectomy in Tandem Occlusions Secondary to Dissection: A STOP-CAD Secondary Study. Stroke. 2025;56(4):808-817. doi: 10.1161/STROKEAHA.124.048295.	F40: STOP-CAD international dissection cohort Sensitivity only: dissection-restricted population (90-day function; sICH)	International STOP-CAD secondary cohort; 63 centres; retrospective multicentre analysis of dissection-related tandem occlusion. Exact recruitment dates are not specified in this secondary report, which refers to the parent STOP-CAD methods (reference 11).	Report total n=328	C versus D (source-defined nonstenting comparator; A/B not separated) Outcomes: 90-day mRS 0-2; sICH; within 24 hours; Mortality; within 90 days; TICI 2b-3 reperfusion; end of procedure; sICH; 24 hours; Mortality; 90 days; 90-day recurrent ischaemic stroke; 90 days; 90-day mRS 0–1; 90 days; TICI 2b-3 reperfusion; End of procedure; Any ICH; 24 hours; Time-to-event symptomatic intracranial haemorrhage through 90 days; 90 days
D00087. Ezcurra-Díaz G, Cardona P, Rodriguez-Villatoro N, Doncel-Moriano Cubero A, Flores-Pina B, Figueras-Aguirre GL, et al. Emergent Carotid Artery Stenting in Patients With Acute Ischemic Stroke With Tandem Lesions: One-Year Follow-Up Results From the SECURIS Study. Neurology. 2025;105(7):e214067. doi: 10.1212/WNL.0000000000214067.	F41: SECURIS/CICAT Catalonia. Descriptive source evidence only; not independently pooled alongside ASSIST and IRIS because centres and recruitment periods overlap. Non-eCAS cannot be split into A and B, and no arm-specific sICH comparison is available.	Ten centres in Catalonia, Spain; retrospective analysis of the prospective mandatory CICAT registry, supplemented by clinical records; January 2017–March 2023.	578 adults: eCAS, 344; non-eCAS, 234. Anterior-circulation LVO with atheromatous cervical ICA stenosis ≥50%, prestroke mRS 0–3 and time from symptom onset or last seen well ≤24 h. CCA-only lesions and dissection did not meet the reported criteria.	C versus unresolved non-eCAS D; the source does not establish an A/B split. Figure 2 yields strict 90-day mRS 0–2 counts of 154/344 versus 84/234. The source-defined favourable endpoint also accepts prestroke mRS 3 patients remaining at 3. Overall sICH: 51; relevant HT: 58 versus 29. Source-specific outcomes appear in Table S8h.

Report citation	Report family and analysis role	Setting, design and recruitment	Population and sample	Cervical strategy and outcomes
D00185. Deliktas, Y, Derraz, I, Finitsis, S, Caroff, J, Bourcier, R, Soize, S, et al. Clinical and safety outcomes of acute stenting plus thrombectomy for carotid tandem lesions with large ischemic core. J Neurointerv Surg. 2025;17(e2):e216-e221. doi: 10.1136/jnis-2024-021517.	F42: ETIS-France_2015-2023 Contextual adjusted evidence (sICH)	France; 30-centre ETIS prospective registry, retrospective analysis of consecutive patients; January 2015-June 2023; 218 tandem-lesion patients with pretreatment ASPECTS 3-5 and known 90-day mRS.	Report total n=218	C versus D (source-defined nonstenting comparator; A/B not separated) Outcomes: sICH; imaging within 24 hours; Mortality; 90 days
D00029. Chen H, McIntyre MK, Lakhani DA, Malhotra A, Colasurdo M, Gandhi D. Emergent Carotid Angioplasty or Stenting for Internal Carotid-Middle Cerebral Artery Tandem Occlusion Strokes: Real-World Outcomes in a Nationwide Cohort. AJNR Am J Neuroradiol. 2026;47(5):1244-1249. doi: 10.3174/ajnr.a9169.	F43: US Nationwide Readmissions Database 2016–2022 Contextual discharge/in-hospital outcomes only; no 90-day function or mortality and no sICH endpoint. Not independently pooled with shorter overlapping NRD reports.	USA; Nationwide Readmissions Database; retrospective administrative cohort; 2016–2022. Number of hospitals not reported in the abstract.	Source abstract reports total 4928, but displayed groups A3371+B541+C1015 sum to 4927. Both source figures are reported, with the one-patient discrepancy noted. Ipsilateral ICA–MCA tandem occlusion treated with EVT.	A:EVT alone 3371; B:EVT+BA541; C:EVT+CAS1015. Outcomes: ordinal discharge functional status, in-hospital mortality, and any ICH during admission. Adjusted C–A effects are available; B–A is described only as nonsignificant.
D00030. Beltrán-Guaquetá JC, Luzardo-Neira CA, Alvarado-Bedoya C, Estevez MF, Serrano S, Reyes A, et al. Comparison of Functional Outcomes and Safety of Acute Carotid Stent Placement versus Thrombectomy Alone in the Treatment of Patients with Tandem Occlusions in Acute Ischemic Stroke. J Vasc Interv Radiol. 2026;37(8):108822. doi: 10.1016/j.jvir.2026.108822.	F44: Colombia single-centre_2016-2025 Sensitivity only: nonstandard follow-up (functional outcome at variable follow-up)	Colombia; single high-complexity referral centre; retrospective cohort; October 2016-June 2025.	Report total n=66	A+B mixed/C / C/D; A; C Outcomes: Functional independence, mRS 0-2; variable follow-up; Mortality; variable follow-up; TICI 2b-3 reperfusion; end of procedure; TICI 2c-3 reperfusion; End of procedure; Variable follow-up beyond 120 days; Follow-up duration; Follow-up

Report citation	Report family and analysis role	Setting, design and recruitment	Population and sample	Cervical strategy and outcomes
D00067. Romoli, M, Molina, C.A, Zapata-Arriaza, E, Jaikumar, V, Jesser, J, Heldner, M.R, et al. Emergent Carotid Stenting for Acute Anterior Circulation Ischemic Stroke With Tandem Lesions: The Multicenter CERES-TANDEM Study. Neurology. 2026;106(2):e214528. doi: 10.1212/WNL.0000000000214528.	F45: CERES-TANDEM_49-centres_2018-2024 Adjusted CERES evidence, reported separately because of overlap	International CERES-TANDEM cohort; 49 comprehensive stroke centres in Europe, North America and Singapore; retrospective longitudinal cohort; January 1,2018-December 31,2024; estimand-based IPTW analysis.	Report total n=4053	C versus D (source-defined nonstenting comparator; A/B not separated) Outcomes: 90-day mRS 0-2; sICH; post-treatment; TICI 2b-3 reperfusion; end of procedure; 90-day ordinal mRS; 90-day mRS 0-1; sICH; Post-treatment safety window (study-defined); 90-day mRS 0–1; 90 days; mRS 0-2 or unchanged from prestroke at 90 days; 90 days; TICI 2b-3 reperfusion; End of procedure; Early neurological deterioration (NIHSS increase ≥4 within 48 h of EVT)
D00081. Cavalcante F, Treurniet KM, Kaesmacher J, Kappelhof M, Rohner R, Yang P, et al. Acute Carotid Stenting for Tandem Lesions in Patients Randomized to Endovascular Treatment With or Without Thrombolysis: Results From the IRIS Individual Participant Data Meta-Analysis. Stroke. 2026;57(1):27-37. doi: 10.1161/STROKEAHA.124.050117.	F46: IRIS IPD: DEVT, DIRECT-MT, SKIP, MR-CLEAN-NO-IV, SWIFT-DIRECT and DIRECT-SAFE_2017-2021 Core non-CERES adjusted evidence and four-node sensitivity analysis (90-day function and sICH)	International individual-participant-data meta-analysis of six trials: DEVT, DIRECT-MT, SKIP, MR CLEAN-NO IV, SWIFT DIRECT and DIRECT SAFE; 2017-2021. Parent trials randomized IV thrombolysis plus EVT versus EVT alone; acute stenting itself was observational.	Report total n=329	C/D; A+B mixed; C Outcomes: 90-day mRS 0-2; sICH; post-treatment safety follow-up; TICI 2b-3 reperfusion; end of procedure; 90-day ordinal mRS; sICH; Post-treatment safety window (study-defined); 90-day mRS 0–1; 90 days; 90-day mRS 5–6; 90 days; NIHSS at 5–7 days; as labelled; Any ICH; Post-treatment safety window (study-defined); Procedure duration; as labelled; Procedure duration; Procedure

Report citation	Report family and analysis role	Setting, design and recruitment	Population and sample	Cervical strategy and outcomes
D00027. Ashouri Y, AlMajali M, Olvany JM, Chaudhari A, Miralbés S, Naravetla B, et al. Clinical and Angiographic Outcomes in Patients With Tandem Occlusion Thrombectomy: Analysis From the ASSIST International Registry. Stroke Vasc Interv Neurol. 2026;6(3):e002157. doi: 10.1161/SVIN.125.002157.	F47: ASSIST_NCT03845491 Four-node sensitivity analysis (90-day function and sICH)	ASSIST international registry; 71 sites in 11 countries; prospective multicentre registry subgroup analysis. Parent-registry recruitment January 2019-January 2022 (Gupta et al., cited as reference 19 in the tandem report); tandem-subgroup-specific first and last recruitment dates are not separately reported.	Report total n=183	C versus D (source-defined nonstenting comparator; A/B not separated) Outcomes: 90-day mRS 0-2; sICH; 24 hours; Mortality; 90 days; TICI 2b-3 reperfusion; end of procedure
D00808. Rosales J, González-Aquines A, Pérez N, Chasco M, Lopez M, Bleise C, Lylyk I, Russo MJ, Lylyk P. Tandem Lesion Strokes: to stent or not to stent, Insights from a Decade of Experience at a Comprehensive Stroke Center in Argentina. medRxiv [Preprint]. 2026 Jan 15. doi:10.64898/2026.01.12.26343969.	F48: Rosales-Argentina_2015-2025 Sensitivity only: preprint (90-day function; sICH)	Argentina; single comprehensive stroke centre; retrospective observational registry; January 2015-July 2025; preprint, not peer reviewed.	Report total n=111	A/B/C / C/D; A; B; C Outcomes: 90-day mRS 0-2; sICH; within 48 hours; Mortality; 90 days; TICI 2b-3 reperfusion; end of procedure; sICH; 48 hours; 90-day ordinal mRS; 90-day mRS 0–1; 90 days; TICI 2b-3 reperfusion; End of procedure; in-hospital mortality; as labelled; early neurological deterioration within 24 hours; 24 hours
D00806. Han N, Jia Y, Zhao Y, Feng G, Xu G. Endovascular Treatment of Tandem Lesions: Prognostic Factors and the Efficacy of Emergent Carotid Stenting. SSRN. 2026. doi: 10.2139/ssrn.6828230.	F49: Han-Jia-Zhao SSRN cohort Strict network: 90-day function and sICH	China; 1 centre; retrospective cohort; Jan 2017–Jan 2025; preprint	Report total n=112. Cervical aetiology: Atherosclerotic only (112/112)	B: BA→MT; C: BA→MT→CAS Outcome sample sizes: mRS 0–2: B38/C74; sICH: B38/C74. Outcomes: 90-day mRS 0-2; sICH; 24-72 hours or index admission; Mortality; 90 days; TICI 2b-3 reperfusion; End of procedure; poor functional prognosis in age at least 65 subgroup

Report citation	Report family and analysis role	Setting, design and recruitment	Population and sample	Cervical strategy and outcomes
D00803. Wichianrat, R, Sangpetngam, B, Aurboonyawat, T, Chankaew, E, Churojana, A, Withayasuk, P. Clinical Outcomes and Predictive Factors in Acute Tandem Occlusion: A Single-Center Experience. Siriraj Medical Journal. 2026;78(5):332-349. doi: 10.33192/smj.v78i5.278546.	F50: Siriraj single-centre cohort. Descriptive source evidence only; not quantitatively synthesized because seven separately labelled common-carotid cases cannot be separated from the eligible cervical-ICA population by acute strategy and outcome. The recoverable 92-person C/D counts do not resolve this anatomical restriction.	Thailand; Siriraj Hospital; retrospective stroke-registry cohort; January 2010-December 2023. Treatment comparisons are explicitly unadjusted.	92 tandem cases: acute CAS20, angioplasty-only 24, no acute cervical intervention 48. Published C-versus-nonstenting comparison includes 79 patients (C20/nonstenting 59) after excluding 13 delayed carotid treatments. Table 1 also includes 7 common-carotid rather than cervical-ICA lesion cases.	Acute C20/B24/A48. Figures 4–6 directly report selected 79 C/non-stent mRS 0–2 13/20 versus 27/59 and sICH1/20 versus 3/59; 13 delayed-treatment patients were excluded from that comparison. Combining the delayed group reconstructs baseline C/D mRS 0–2 13/20 versus 38/72 and sICH1/20 versus 3/72. This full 92 reconstruction does not resolve anatomical eligibility.

Report IDs are stable links to the screening records. Family IDs group related or potentially overlapping reports and do not identify independent patient cohorts.

The catalogue contains 60 reports grouped into 49 report or cohort families.

The strict three-node network contains 16 cohorts: 15 contributed to 90-day functional independence and 15 to sICH. Wallocha 2019 contributed sICH only; Özdemir 2025 contributed functional independence only.

Table S5 distinguishes fitted-analysis membership, companion reports and descriptive source evidence. Table S8h provides additional source results and final synthesis roles. Fahed, IRETAS and SECURIS are retained as descriptive evidence, with overlap and source qualifications stated. Nolan remains contextual because raw arm counts and adjustment covariates are unavailable; Wichianrat remains descriptive because eligible ICA-only outcomes cannot be isolated. EASI and CERES-TANDEM are reported separately.

A, no acute cervical treatment; B, angioplasty alone; C, emergent stenting; D, mixed non-stenting management where A and B are not separable. AIS, acute ischaemic stroke; CAS, carotid artery stenting; EVT, endovascular therapy; ICA, internal carotid artery; ICH, intracranial haemorrhage; LVO, large-vessel occlusion; mRS, modified Rankin Scale; sICH, symptomatic ICH; TICI, Thrombolysis in Cerebral Infarction.

Setting and outcome fields were checked against the identified sources, including the full Fahed, Garg and SECURIS reports and Garg supplement. Remaining unavailable fields mean not reported in the inspected source, an unverifiable outcome denominator or a source conflict, as specified in the relevant cell; they do not denote zero events. The SECURIS supplement was not available, but its main report supplies the explicitly qualified functional reconstruction below.

L56, L57 and L63 describe the same 73-patient CHUM cohort and are represented once for outcomes and assessment coverage. L58 is an interim EASI report. Conference-supplement DOIs may be shared across abstracts; author, title and page identify each report.

The overlap-restricted four-node analysis contained 19 cohorts for functional independence and 20 for sICH. Its outcome-specific inputs are reported in Tables S7 and S8b.

Table S6a. Outcome-specific risk-of-bias judgements for the nonrandomized strict-network studies

Study	Outcome	Nodes	D1	D2	D3	D4	D5	D6	D7	Overall
Marnat/RECOST 2016	mRS 0–2	A/C	Critical	Low	Low	Low	Low	Moderate	Moderate	Critical
Marnat/RECOST 2016	sICH	A/C	Critical	Low	Low	Low	Low	Moderate	Moderate	Critical
Dorado 2017	mRS 0–2	B/C	Serious	Moderate	Low	No information	Serious	No information	Moderate	Serious

Study	Outcome	Nodes	D1	D2	D3	D4	D5	D6	D7	Overall
Dorado 2017	sICH	B/C	Serious	Moderate	Low	No information	Low	No information	Moderate	Serious
Eker 2018	mRS 0–2	A/B/C	Serious	Low	Low	Low	Low	Moderate	Moderate	Serious
Eker 2018	sICH	A/B/C	Serious	Low	Low	Low	Low	Moderate	Moderate	Serious
Li W 2018	mRS 0–2	B/C	Serious	Low	Low	Low	Low	Low	Moderate	Serious
Li W 2018	sICH	B/C	Serious	Low	Low	Low	Low	Moderate	Moderate	Serious
Papanagiotou/TITAN 2018	mRS 0–2	A/B/C	Serious	Low	Low	Low	Low	Moderate	Moderate	Serious
Papanagiotou/TITAN 2018	sICH	A/B/C	Serious	Low	Low	Low	Low	Moderate	Moderate	Serious
Jadhav/STRATIS 2019	mRS 0–2	B vs C	Serious	Serious	Low	Low	Moderate	Moderate	Moderate	Serious
Jadhav/STRATIS 2019	sICH	B vs C	Serious	Serious	Low	Low	Moderate	Moderate	Moderate	Serious
Kang 2019	mRS 0–2	B/C	Critical	Low	Low	Low	Low	Moderate	Moderate	Critical
Kang 2019	sICH	B/C	Critical	Low	Low	Low	Low	Moderate	Moderate	Critical
Wallocha 2019	sICH	B vs C	Serious	Low	Low	Low	Low	Moderate	Moderate	Serious
Kim B 2020	mRS 0–2	B vs C	Serious	Serious	Moderate	Low	Low	Moderate	Moderate	Serious
Kim B 2020	sICH	B vs C	Serious	Moderate	Moderate	Low	Low	Moderate	Moderate	Serious
Gigliotti (Salem cohort) 2021	mRS 0–2	B vs C	Serious	Serious	Low	Low	Moderate	Moderate	Moderate	Serious
Gigliotti (Salem cohort) 2021	sICH	B vs C	Serious	Serious	Low	Low	Low	Moderate	Moderate	Serious
Kalmar 2021	mRS 0–2	B vs C	Serious	Moderate	Low	Low	Moderate	Moderate	Moderate	Serious
Kalmar 2021	sICH	B vs C	Serious	Moderate	Low	Low	Low	Moderate	Moderate	Serious
Li W 2021	mRS 0–2	C vs B	Serious	Low	Low	Low	Low	Moderate	Moderate	Serious
Li W 2021	sICH	C vs B	Serious	Low	Low	Low	Low	Moderate	Moderate	Serious
Yu M 2023	mRS 0–2	C vs B	Serious	Low	Low	Low	Moderate	Moderate	Moderate	Serious
Yu M 2023	sICH	C vs B	Serious	Low	Low	Low	Moderate	Moderate	Moderate	Serious

Study	Outcome	Nodes	D1	D2	D3	D4	D5	D6	D7	Overall
Faybushevich/Galustyan 2025	mRS 0–2	C vs A	Serious	Moderate	Low	Low	Low	Moderate	Moderate	Serious
Faybushevich/Galustyan 2025	sICH	C vs A	Serious	Moderate	Low	Low	Low	Moderate	Moderate	Serious
Özdemir 2025	mRS 0–2	C vs A	Serious	Low	Moderate	Low	Moderate	Moderate	Moderate	Serious
Han N 2026 (SSRN preprint)	mRS 0–2	C vs B	Serious	Low	Low	Low	Moderate	Moderate	Moderate	Serious
Han N 2026 (SSRN preprint)	sICH	C vs B	Serious	Low	Low	Low	Moderate	No information	Moderate	Serious

D1, confounding; D2, participant selection; D3, intervention classification; D4, deviations from intended interventions; D5, missing data; D6, outcome measurement; D7, selection of the reported result. Ratings use the seven ROBINS-I-aligned domains described in the Methods.

The table covers 30 study-outcome results from 16 strict-network cohorts: 15 functional-independence and 15 sICH results. Wallocha contributes sICH only and Özdemir contributes functional independence only. Overall ratings are serious for 26 results and critical for four results; confounding drives the overall judgements. The EASI randomized assessment is reported separately in Table S3.

Table S6b. Additional nonrandomized result-level risk-of-bias assessments

Study/result	Outcome	Nodes	D1	D2	D3	D4	D5	D6	D7	Overall
Madden 2023 / Northern Ireland D00989 / adj	mRS 0–2, 90 d	C vs D	S	NI	L	NI	NI	NI	NI	S
Sousa 2025 / STOP-CAD D00141 / adj	mRS 0–2, 90 d	C vs D	S	L	L	L	M	M	NI	S
Sousa 2025 / STOP-CAD D00141 / adj	sICH	C vs D	S	L	L	L	L	M	NI	S
Deliktas 2025 / ETIS large core D00185 / adj	sICH	C vs D	S	M	L	L	M	M	NI	S
Romoli 2026 / CERES-TANDEM D00067 / IPTW-1	mRS 0–2 or unchanged	C vs D	S	L	L	L	M	M	NI	S
Romoli 2026 / CERES-TANDEM D00067 / IPTW-1	sICH	C vs D	S	L	L	L	M	M	NI	S
Cavalcante 2026 / IRIS D00081 / adj	mRS 0–2, 90 d	C vs D	S	L	L	L	M	M	NI	S
Cavalcante 2026 / IRIS D00081 / adj	sICH	C vs D	S	L	L	L	M	M	NI	S

Study/result	Outcome	Nodes	D1	D2	D3	D4	D5	D6	D7	Overall
Rosales 2026 / Argentina<br>D00808 / adj	mRS 0–2, 90 d	C vs D	S	L	L	L	S	M	NI	S
Rosales 2026 / Argentina<br>D00808 / adj	sICH	C vs D	S	L	L	L	L	M	NI	S
Marko 2022 / ESCAPE-NA1<br>D00403 / adj	mRS 0–2, 90 d	C vs D	S	L	L	L	M	M	NI	S
Beltrán-Guaquetá 2026 / Colombia<br>D00030 / adj	mRS 0–2 (variable follow-up)	C vs D	S	L	L	L	L	S	NI	S
Farooqui 2023 / PSM<br>D00285 / PSM	mRS 0–2, 90 d	C vs D	S	L	L	L	NI	M	NI	S
Farooqui 2023 / PSM<br>D00285 / PSM	sICH	C vs D	S	L	L	L	NI	M	NI	S
Cavalcante 2026 / IRIS<br>D00081 / raw	mRS 0–2, 90 d	C vs D	S	L	L	L	M	M	NI	S
Cavalcante 2026 / IRIS<br>D00081 / raw	sICH	C vs D	S	L	L	L	M	M	NI	S
Marko 2022 / ESCAPE-NA1<br>D00403 / raw	mRS 0–2, 90 d	C vs D	S	L	L	L	L	M	NI	S
Marko 2022 / ESCAPE-NA1<br>D00403 / raw	sICH	C vs D	S	L	L	L	L	M	NI	S
Ashouri 2026 / ASSIST<br>D00027 / raw	mRS 0–2, 90 d	C vs D	S	S	L	L	M	M	NI	S
Ashouri 2026 / ASSIST<br>D00027 / raw	sICH	C vs D	S	S	L	L	L	M	NI	S
Kojima 2023<br>D00283 / raw	sICH	C vs D	S	L	L	L	L	M	NI	S

The 21 rows cover 14 selected adjusted results and 7 additional unadjusted four-node results; repeated cohorts represent different outcomes or models, not independent studies.

L, low; M, moderate; S, serious; C, critical; NI, no information. D1, confounding; D2, selection of participants; D3, intervention classification; D4, deviations from intended interventions; D5, missing data; D6, outcome measurement; D7, selection of the reported result. Overall serious reflects at least one serious domain; NI is not evidence of low risk.

D is the intact mixed nonstenting comparator and is not a pure angioplasty-only or no-treatment node. Ratings concern the specified result and assignment-policy target; routine antiplatelet variation or later treatment is not automatically a D4 deviation. Farooqui PSM is not a clean baseline-adjusted total-effect estimate.

Table S6c provides source pages, model and estimand distinctions, and domain-specific grounds. NI indicates insufficient information for a domain judgement.

Table S6c. Source-based grounds for the additional risk-of-bias assessments

Study/source	Result, model and estimand	Domain-specific grounds and source pages
Madden 2023 / Northern Ireland / D00989	NIHSS-adjusted logistic model (EFF0007).<br>Association of acute stenting versus intact nonstenting group with 90-day mRS 0–2; assignment-policy target, not an adherence effect.<br>Results: EFF0007	D1: Only baseline NIHSS was adjusted; age, prestroke disability, infarct extent and treatment-selection anatomy were not controlled (abstract 567, journal p. 227 / PDF p. 225).<br>D2: Of 115 tandem patients, 96 met unspecified inclusion criteria; the timing and reasons for exclusion of 19 are not described (p. 227).<br>D3: Acute extracranial ICA stenting is explicitly recorded in 30 patients; the nonstenting group remains an intact mixed comparator (p. 227).<br>D4: Operator-discretion treatment and antiplatelets are stated, but the abstract gives insufficient information on deviations and subsequent management (p. 227).<br>D5: The denominator and handling of missing 90-day mRS or model covariates are not reported; stent-imaging follow-up is not a substitute for functional follow-up (p. 227).<br>D6: mRS 0–2 at 3 months is defined, but assessor training, blinding and ascertainment methods are not given (p. 227).<br>D7: No protocol or analysis plan is provided in the available conference abstract, so selection among eligible analyses cannot be assessed (p. 227).<br>Source qualification: Assessment is restricted to the original conference abstract, not a full-length article. Journal p. 227 corresponds to PDF p. 225.
Sousa 2025 / STOP-CAD / D00141	Primary mixed-effects logistic models adjusted for age and NIHSS, with site effects (EFF0011/EFF0012); not the ASPECTS or IPTW sensitivity models.<br>Assignment-policy association in occlusive carotid dissection; 90-day mRS 0–2 and 24-hour sICH.<br>Results: EFF0011, EFF0012	D1: The selected model adjusts only age and NIHSS. Baseline ASPECTS differs; collateral anatomy, residual lesion and reperfusion immediately before stent selection were unavailable (PDF pp. 2–4, 8 / journal pp. 809–811, 815).<br>D2: The cohort is defined by occlusive dissection, acute stroke and EVT within 1 day of diagnosis, rather than by achieved outcome (pp. 2–3).<br>D3: Index-procedure stent placement is explicit; lack of angioplasty detail does not invalidate the intact stent/nonstent contrast (pp. 2, 8).<br>D4: The assignment-policy contrast includes routine periprocedural care. Different antiplatelet strategies alone are not classified as deviations from this policy (pp. 2–3, 5).<br>D5: Functional data are available for 274/328 and the selected functional model for 270; exclusions are 20/150 versus 34/178 before covariate deletion. Available-case analysis is used without an outcome-missingness correction. sICH is observed in 328/328 and modelled in 324 (pp. 3, 5).<br>D6: Local investigators explicitly reported outcomes unblinded to intervention; the clinical component of mRS and sICH can be influenced despite defined endpoints (pp. 2–3).<br>D7: Several prespecified-looking models are described, but an analysis plan predating this secondary question was not available in the inspected report; selective reporting cannot be verified (pp. 3, 8).

Study/source	Result, model and estimand	Domain-specific grounds and source pages
Deliktas 2025 / ETIS large core / D00185	Logistic model adjusted for age, admission NIHSS, IV thrombolysis and onset-to-puncture time; Table 1 (EFF0015).<br>Assignment-policy association of acute stenting versus no stenting in tandem lesions with ASPECTS 3–5; sICH within 24 hours.<br>Results: EFF0015	D1: Operator-discretion stenting and baseline group differences remain; the four-variable adjustment does not cover all lesion, collateral, prestroke and centre-level treatment-selection factors (PDF pp. 2–3).<br>D2: Eligibility additionally requires known 90-day mRS even for this early safety comparison. Numbers excluded for unavailable mRS are not given, so selection related to later prognosis remains possible (p. 2 and Fig. 1, p. 3).<br>D3: For patients with known exposure, acute stenting versus no stenting is explicitly defined and recorded; the 22 unknown exposures are considered under missing data (pp. 2–3).<br>D4: Usual-care antiplatelets and subsequent management are part of the assignment-policy contrast; variation itself is not an adherence/deviation failure (pp. 2–3).<br>D5: Stent status is missing in 22/218. Table 1 does not explicitly provide the outcome-specific sICH denominators, and missingness handling is not specified; denominators are not reconstructed from rounded percentages (p. 3).<br>D6: sICH requires imaging within 24 hours plus NIHSS worsening or death. Local clinical/imaging assessment is used; the reported blinding applies specifically to 90-day mRS, not demonstrably to sICH (p. 2).<br>D7: The registry is identified, but a result-specific analysis plan was not available in the inspected report; selection among secondary safety analyses cannot be verified (pp. 2–3).
Romoli 2026 / CERES-TANDEM / D00067	Estimand 1 stabilized IPTW, treatment-policy total-effect model (EFF0021/EFF0023). Baseline adjustment spans demographics, vascular risk, prestroke mRS/therapy, imaging, NIHSS, occlusion, IVT and treatment timing.<br>Whole-population treatment-policy effect of index eCAS versus conservative management, allowing later crossover; functional endpoint is mRS 0–2 OR unchanged from prestroke, not strictly mRS 0–2.<br>Results: EFF0021, EFF0023	D1: Extensive baseline IPTW is used, but procedural success and anatomy influence whether stenting is undertaken; the report notes that eCAS may be avoided without full recanalization. Baseline weights do not resolve this dynamic confounding by indication (PDF pp. 3–4, 8–9).<br>D2: Consecutive eligible tandem patients are identified using baseline clinical/imaging criteria in 49 centres; estimand 1 retains the whole population (pp. 2–4).<br>D3: The index procedure defines eCAS versus conservative management. Later crossover is explicitly distinguished rather than retrospectively relabelling baseline exposure (pp. 3–4).<br>D4: The treatment-policy estimand explicitly permits subsequent crossover and usual care, so later carotid procedures and antiplatelets are not themselves protocol deviations (pp. 3–4, 8).<br>D5: Data queries and imputation for covariates with less than 3% missingness are described, with exclusion otherwise. The inspected report does not fully enumerate endpoint/model-specific exclusions after these procedures (pp. 3–4, 8).<br>D6: Clinical and imaging outcomes were obtained locally from records/registries with site review and queries rather than blinded central adjudication (pp. 3, 9–10).<br>D7: A registered protocol and analysis plan are referenced, but that plan was not independently retrieved and compared with the selected binary results in this assessment (pp. 3–4).<br>Source qualification: Estimand 2 adjusts for recanalization/sICH; estimand 3 uses a restricted population. Neither applies to estimand 1, whose functional endpoint includes unchanged prestroke mRS.

Study/source	Result, model and estimand	Domain-specific grounds and source pages
Cavalcante 2026 / IRIS / D00081	Selected mixed logistic models (EFF0028/EFF0029): age, sex, NIHSS, onset-to-randomization, prestroke mRS, occlusion location, AF, IVT allocation; trial effects. Additional raw C/D results are assessed separately, not assigned the adjusted-model protections.<br>Assignment-policy association of acute carotid stenting with 90-day mRS 0–2 or sICH. The underlying trials randomized IVT, not carotid stenting.<br>Results: EFF0028, EFF0029, RAW_D00081_mrs, RAW_D00081_sich	D1: Carotid stenting remains nonrandom and operator selected. Main adjustment does not capture detailed carotid anatomy/etiology, stent timing or the decision-stage reperfusion state; the raw four-node result has no covariate adjustment (PDF pp. 2–4, 7–8 / journal pp. 28–30, 33–34).<br>D2: Baseline tandem cases are identified within six trials by imaging definitions; no explicit post-outcome eligibility restriction is reported. Missing exposure is assessed under D5 (pp. 2–4).<br>D3: Acute stenting, angioplasty-only and deferred/medical treatment are distinguished. The intact no-acute-stent comparator is kept mixed rather than recoded as a pure node A or B (p. 3).<br>D4: The assignment-policy target incorporates ordinary antiplatelet and later treatment choices; lack of a common adherence protocol is not automatically a deviation (pp. 3, 8).<br>D5: Eleven of 340 tandem patients lack stenting data and are excluded. Among 329 classified patients, only one mRS and two sICH observations are missing; multiple imputation is used for model data. Raw estimates use reported available-case denominators (pp. 4, 7).<br>D6: Standard mRS and imaging-plus-NIHSS sICH definitions are stated, but blinding of endpoint ascertainment for the stenting comparison is not established by the parent trials randomizing IVT (pp. 2–3).<br>D7: Several outcomes and alternative weighting models are reported. A stenting-specific, predating analysis plan was not available for verification in the inspected material (pp. 3–4).
Rosales 2026 / Argentina / D00808	Table 2 age-, sex- and admission-NIHSS-adjusted models (EFF0034/EFF0035); not the larger multivariable models in Tables 3–4.<br>Assignment-policy association of acute stenting versus intact mixed MT-alone management; 90-day mRS 0–2 and ECASS-III sICH within 48 hours.<br>Results: EFF0034, EFF0035	D1: Operator preference, anatomy and clinical factors determine stenting. Adjustment for age, sex and NIHSS does not control baseline infarct extent, lesion detail or treatment-era effects across the decade (preprint PDF pp. 5–7, 9, 12–14).<br>D2: Consecutive adults are selected by baseline tandem lesions and EVT within 24 hours; no outcome-based enrolment criterion is stated (p. 5).<br>D3: Stenting during thrombectomy is explicit; MT-alone includes balloon, aspiration and deferred/no carotid procedures and is retained as a mixed comparator (p. 5).<br>D4: Usual-care antithrombotic and cervical management choices belong to the assignment-policy contrast; they are not treated as nonadherence merely because they differ between groups (pp. 5–7, 13).<br>D5: Functional data are reported for only 87/111: 62/74 stented versus 25/37 nonstented, with differential attrition and no imputation/selection correction described. sICH is reported for 110/111 (19/73 versus 9/37) and has a different, low missing-data concern (pp. 6, 14).<br>D6: mRS is obtained from medical records and sICH uses ECASS-III criteria, but blinded independent outcome adjudication is not documented (pp. 5–6, 14).<br>D7: This assessment uses the available January 2026 preprint. It reports multiple functional/model definitions without an accessible predating analysis plan; selection and agreement with the final publication remain unverified (pp. 6–7, 14, 16–17).<br>Source qualification: Source is the medRxiv preprint, not the final journal version. The Methods give recruitment dates of January 2015–July 2025.

Study/source	Result, model and estimand	Domain-specific grounds and source pages
Marko 2022 / ESCAPE-NA1 / D00403	Baseline multivariable logistic mRS model (EFF0039) plus separate raw C/D functional and sICH results. Covariates: age, sex, NIHSS, ASPECTS, occlusion location, nerinetide-by-alteplase interaction.<br>Assignment-policy association of acute stenting versus no stenting in complete cervical carotid occlusion; 90-day mRS 0–2 and 24-hour Heidelberg sICH. Nerinetide, not stenting, was randomized.<br>Results: EFF0039, RAW_D00403_mrs, RAW_D00403_sich	D1: Stenting is operator selected and mostly follows intracranial treatment; reperfusion was 98.4% versus 67.9%. Reasons for nonstenting after thrombectomy were not recorded. Baseline adjustment does not remove this decision-stage confounding; raw results are unadjusted (PDF pp. 2–4 / journal pp. 430–432).<br>D2: The 115 cases are defined by pretreatment/initial angiographic complete cervical occlusion within the trial cohort, without a reported outcome-based selection condition (pp. 1–2).<br>D3: Case-report forms document stent placement and timing; uncertainty about angioplasty-only detail does not erase the acute stent/no-stent distinction (pp. 2, 4).<br>D4: Usual clinical care, including operator-dependent sequence and antiplatelets, is part of the assignment-policy comparison; no adherence contrast is claimed (pp. 2–3).<br>D5: All 115 have reported functional and sICH denominators (62 stented, 53 nonstented). For the adjusted model, completeness of covariates is not separately quantified and remains a moderate concern (pp. 2–3).<br>D6: Imaging and sICH definitions are standardized, but blinding of the mRS/clinical sICH assessment to carotid stenting is not documented in this secondary report (p. 2).<br>D7: The study is a secondary subgroup analysis with several outcomes and models; a predating carotid-management analysis plan was not available for verification (pp. 2–4).
Beltrán-Guaquetá 2026 / Colombia / D00030	Final Table 3 multivariable logistic model (EFF0040), retaining CAS and admission NIHSS. AF was considered in the analysis but was not included in the reported final model.<br>Assignment-policy association with mRS 0–2 at available, nonstandard follow-up (group means 151.9 versus 206.7 days), not a fixed 90-day estimand.<br>Results: EFF0040	D1: Stenting follows intracranial recanalization when the lesion remains flow limiting; unsuccessful procedures are concentrated in the nonstented arm. NIHSS-only final adjustment does not address anatomy, procedure-stage selection, AF/anticoagulants or treatment era (PDF pp. 3, 5–9).<br>D2: All 66 baseline-eligible tandem cases are identified from 421 EVT patients; exclusions relate to tandem status or prestroke mRS, not follow-up outcome (p. 2).<br>D3: Index carotid stenting versus EVT without a permanent carotid stent is explicitly described in procedural records; the comparator includes balloon-assisted crossing (pp. 2–3).<br>D4: Routine antiplatelet and subsequent care form part of the assignment-policy contrast; differential cointerventions alone do not establish deviations (pp. 3, 5, 8).<br>D5: The follow-up mRS table accounts for all 66 patients, including deaths (21 versus 45); no additional model exclusion is reported (pp. 6–7).<br>D6: mRS is assessed at variable available follow-up, with materially different group mean durations and no time-to-event/standardized-time adjustment. This undermines comparability of the measured functional endpoint; blinded ascertainment is also not stated (pp. 5–6, 9).<br>D7: The final model omits AF although broader descriptions say it was considered; several endpoints/time points are reported without a verifiable predating analysis plan (pp. 4, 7–9).<br>Source qualification: This result is presented as nonstandard-follow-up sensitivity or contextual evidence. The Methods state recruitment from October 2016 to June 2025.

Study/source	Result, model and estimand	Domain-specific grounds and source pages
Farooqui 2023 / PSM / D00285	1:1 propensity-score-matched comparison (EFF0053/EFF0057), caliper 0.2 SD. Supplement eFigure 2 includes sex, race, NIHSS, periprocedural antiplatelets and cervical stenosis.<br>Matched association of acute CAS versus intact nonstenting care with 90-day mRS 0–2 or ECASS-III sICH; not a clean baseline-adjusted total treatment-policy effect because matching conditions on periprocedural treatment.<br>Results: EFF0053, EFF0057	D1: The propensity score includes periprocedural antiplatelets, potentially conditioning on a consequence/cointervention of stenting, and does not comprehensively cover age, infarct burden and decision-stage anatomy. The selected PSM contrast therefore remains seriously confounded (main PDF pp. 3–5, 11; supplement PDF p. 8, eFigure 2).<br>D2: The underlying 17-centre cohort includes consecutive baseline-eligible patients with waived consent. Matching defines the analysis population, but no outcome-driven enrolment criterion is stated (main pp. 2–3).<br>D3: Acute CAS during EVT is recorded; balloon/aspiration, deferred carotid intervention and no intervention remain combined in the nonstenting comparator (main pp. 2–3).<br>D4: The index strategy allows routine subsequent care. The problem with periprocedural antiplatelet matching belongs to confounding/estimand definition, not an invented adherence protocol (main pp. 3–4; supplement p. 8).<br>D5: The matched analytic N and PSM-specific missing-data handling are not provided in the inspected PSM report/eFigure. Multiple imputation described for main regression cannot be assumed to validate this different matched analysis (main pp. 4–5, 7; supplement p. 8).<br>D6: mRS is assessed by neurologist visit or certified nurse telephone interview; no treatment-blind assessment is stated and there is no independent core adjudication of clinical/imaging outcomes (main pp. 3, 11).<br>D7: Multiple adjusted, unadjusted and PSM analyses are presented; no predating PSM-specific analysis plan was available for comparison (main pp. 4–5; supplement p. 8).<br>Source qualification: Supplementary eFigure 2 (p. 8) reports the selected ORs and matching variables. The full cohort N=623 is not the matched sample size.
Ashouri 2026 / ASSIST / D00027	Raw four-node C/D results from Table 3; not the multivariable mRS model that includes final eTICI.<br>Assignment-policy association of acute stenting versus intact no-stent management with 90-day mRS 0–2 or 24-hour sICH.<br>Results: RAW_D00027_mrs, RAW_D00027_sich	D1: Raw comparisons do not adjust the substantial differences in age, prestroke function and AF or the operator-selected treatment strategy; protection from the separate adjusted model cannot be transferred (PDF pp. 3–4, 6, 8).<br>D2: The report explicitly notes nonconsecutive recruitment and retrospective post-procedure consent, potentially excluding poor-outcome patients. Equal effects on treatment arms are asserted but not demonstrated (pp. 7–8).<br>D3: All stented patients in this comparison underwent acute stenting, and treatment is recorded prospectively (pp. 3–4, Table 3 p. 8).<br>D4: The assignment-policy contrast includes standard operator-directed management and antiplatelets; no adherence-restricted effect is claimed (pp. 3, 7–8).<br>D5: mRS is available in 79/83 versus 92/100, with reasons/handling of 12 missing observations not detailed. sICH is reported in all 183 patients and has low missing-data concern (Table 3, p. 8).<br>D6: An independent imaging core laboratory assesses hemorrhagic complications, but blinding for the clinical component of sICH and 90-day mRS is not explicitly established (p. 3).<br>D7: The prospective registry is registered, but a carotid-stenting secondary-analysis plan was not available for verification; several subgroup and outcome definitions are reported (pp. 2–4, 8).

Study/source	Result, model and estimand	Domain-specific grounds and source pages
Kojima 2023 / D00283	Unadjusted four-node C/D safety comparison from Table 2; no covariate-adjusted selected model. Assignment-policy association of acute carotid stenting versus mixed non-CAS management with sICH within 14 days; the paper functional endpoint is mRS ≤3 and is not used as mRS 0–2. Results: RAW_D00283_sich	D1: Stenting is selected after insufficient PTA or delayed perfusion, without fixed criteria; baseline NIHSS and infarct scores differ and no multivariable adjustment is used (PDF pp. 2–4, 9 / journal pp. e209–e211, e216).<br>D2: The cohort is defined by baseline tandem lesions in four hospitals, with dissection excluded; no post-outcome eligibility restriction is reported (p. 2).<br>D3: CAS versus non-CAS is explicit. Non-CAS contains 12 PTA and 2 untreated cases and is kept as an intact mixed comparator (pp. 3–4).<br>D4: Routine rescue selection and antiplatelets are described; with an assignment-policy target, subsequent usual care is not an adherence violation (pp. 3–4).<br>D5: Table 2 reports sICH for all 50 patients, with 4/36 stented and 0/14 non-CAS events (p. 3).<br>D6: CT is triggered by neurological worsening; sICH requires hematoma/SAH plus NIHSS worsening ≥4 within 14 days. Unblinded clinical detection/adjudication remains a concern (p. 3).<br>D7: Multiple imaging and clinical outcomes are examined without a verifiable predating analysis plan in the inspected report (pp. 3–4, 9).

Page numbers refer to the source PDF unless journal pagination is also specified. IRIS uses the 11-page journal PDF (journal pp. 27–37), not the repository copy with an additional cover page. Northern Ireland is the conference abstract, and Argentina is the available preprint.

D5 differs by outcome where stated: STOP-CAD functional M versus sICH L; Argentina functional S versus sICH L; ASSIST functional M versus sICH L. ESCAPE-NA1 raw outcomes have D5 L, while the adjusted model has D5 M because covariate completeness is not separately quantified. D7 NI identifies an unverified predating plan, not proven selective reporting.

Framework: original ROBINS-I (2016), https://www.riskofbias.info/welcome/home/original-2016-version-of-robins-i; interpretation of result-specific domains and overall judgement: Cochrane Handbook, Chapter 25, https://www.cochrane.org/authors/handbooks-and-manuals/handbook/current/chapter-25.

Table S6d. Source evidence and assessment coverage for additional report families

Family / report IDs	Source-supported information relevant to bias	Assessment coverage	Source
F04 / L37	D1: Retrospective treatment selection. Demographic adjustment is stated for treatment-use associations; the outcome model's full covariate set is not described. D2: National discharge-weighted sample; eligibility and overall totals/dates conflict within abstract. D3: CAS versus angioplasty identified administratively; procedure coding validity is not described in abstract. D4: Periprocedural antithrombotics, rescue procedures and crossovers are not described. D5: Missingness and outcome-specific denominators not reported. D6: Inpatient mortality/discharge endpoints; no 90-day mRS or sICH adjudication. D7: Outcome ORs reported without CIs; prospectively specified analysis plan not available. Selective reporting cannot be established from this information.	Source evidence recorded; available abstract is insufficient for a complete result-specific seven-domain assessment. No final rating assigned.	Table S5 report citation; Table S8h source locator.

Family / report IDs	Source-supported information relevant to bias	Assessment coverage	Source
F05 / L38, L41, L42, L44	L38/L41 remain abstract-level companion evidence. L38: propensity matching includes demographic, comorbidity, thrombolysis and hospital variables; stroke severity/anatomy, target tandem status in all controls, same-procedure timing, deviations and endpoint missingness are unresolved. Combined CAS/angioplasty is not a separable node. L41: emergent-versus-delayed CAS is related administrative evidence, not another independent co-primary sample. L42/L44: Full publication and supplementary data. D1: Figure 1 adjusts for age, sex, income, antiplatelets and IV thrombolysis; NIHSS, infarct/anatomical detail and procedural timing unavailable. D2: C/B inpatient cohort is admission-defined; readmission analysis conditions on discharge survival. D3: ICD-coded same-day C/B treatment without individual angiographic validation. D4: Later CAS/CEA belongs to usual-care initial-policy follow-up. D5: Survey-estimated totals; unweighted/model-specific N and missing-data handling unavailable. D6: Coded ICH is not sICH; death/home are objective but not 90-day function. D7: No accessible prospective plan; Figure 3's 90-day caption conflicts with 30-day methods/results.	L38 and L41 contribute source information only; L42 is not rated separately from its full publication. Three L44 result-level rows cover inpatient haemorrhagic and complication results, objective disposition outcomes, and readmission.	L38/L41: citations in Table S5. L42/L44: Garg 2021, PDF pp2–5, Table 1/Figure 1; supplement Tables S1–S3 (pp1–4); doi:10.1016/j.jns.2021.117588.
F13 / L50	Not applicable to the present co-primary synthesis because no cervical-strategy comparative result is extractable. This is not a low-risk judgment.	No eligible target-strategy comparative result is available from this report for the current network. This is not a low-risk judgment; no result-level network rating assigned.	Table S5 report citation; Table S8h source locator.
F14 / L52	D1: No randomized assignment or confounder-adjusted effects reported. D2: 120 tandem cases among 822 thrombectomy patients over June 2010-October 2017; completeness of case ascertainment not described. D3: No carotid treatment, angioplasty and stenting groups explicitly separated; procedural sequence not given. D4: Antiplatelets, rescue treatment and other cointerventions not reported. D5: Explicit reperfusion event counts available; denominator of untreated group derived from full treatment partition. Three-month arm follow-up denominators unavailable. D6: TICI 2b-3 named; assessor blinding not described. sICH definition absent; printed percentages inconsistent with event counts. D7: Only reperfusion numerically reported by strategy, function/sICH only qualitatively; no accessible prespecified plan.	Source evidence recorded; available abstract is insufficient for a complete result-specific seven-domain assessment. No final rating assigned.	Table S5 report citation; Table S8h source locator.
F16 / L54	D1: Retrospective nine-patient cohort with selected cervical treatments; no adjustment or baseline balance analysis reported. D2: Nine endovascularly treated tandem cases, July 2012-December 2017; complete/consecutive inclusion not established. D3: Acute angioplasty with or without concurrent carotid stent explicitly described; B3 and C6. D4: Antithrombotics and other cointerventions not described. D5: 90-day mRS ≤1 counts are reported as 3/6 and none of 3; detailed follow-up/missingness methods absent. D6: TICI and 90-day mRS named; mRS threshold ≤1. Blinding/sICH ascertainment not reported. D7: Collected endpoints named, but discharge NIHSS numerical values and sICH results not supplied; prespecified plan unavailable.	Source evidence recorded; available abstract is insufficient for a complete result-specific seven-domain assessment. No final rating assigned.	Table S5 report citation; Table S8h source locator.

Family / report IDs	Source-supported information relevant to bias	Assessment coverage	Source
F17 / L56, L57, L63	D1: Hypertension imbalance (77% vs 45%) and nonrandomized treatment choice; no adjusted outcome effects. D2: All tandem patients admitted for EVT to CHUM Notre-Dame December 2014-December 2016, according to abstract. D3: Acute stent classified, but nonstenting procedures not split into A/B. D4: Antiplatelet/cointervention details, rescue treatment and deviations unavailable. D5: Only baseline arms 26/47 and rounded outcome percentages; endpoint-specific denominators and follow-up completeness not explicit. D6: 90-day mRS 0-2, sICH and TICI 2b-3 named; sICH definition and assessor blinding unavailable. D7: Same results in all three reports; accessible protocol not identified. Multiple abstracts are duplicate reporting, not independent studies.	Source evidence recorded; available abstract is insufficient for a complete result-specific seven-domain assessment. No final rating assigned.	Table S5 report citation; Table S8h source locator.
F19 / L59	D1: Prospective but nonrandomized cervical treatment; no adjusted effects, baseline groups described only as comparable. D2: Consecutive non-cardioembolic tandem cases treated within six hours, April 2013-April 2017. D3: B40/C52 explicitly separated; self-expanding and balloon-expanding stents both map to C. D4: All self-expanding stents received early antiplatelets; 75% of balloon-expanding cases had none within 24 hours. D5: Baseline n40 does not yield an exact 86.5% fraction; assessed denominator for restenosis not supplied. Clinical follow-up denominators unavailable. D6: mRS ≥2 printed in source; no group mRS results. HT reported without total sICH counts; outcome blinding not described. D7: Several specified endpoints only qualitatively reported; no prospectively accessible analysis plan.	Source evidence recorded; available abstract is insufficient for a complete result-specific seven-domain assessment. No final rating assigned.	Table S5 report citation; Table S8h source locator.
F20 / L65	D1: Adjusted ordinal model includes age, NIHSS, time to recanalization, TICI and sICH; latter variables may be downstream of treatment, so adjustment does not establish total-effect control. D2: Retrospective 2012-2015 stroke-centre cohort, n60; subgroup analysis by aetiology; full eligibility details unavailable. D3: CAS recorded (32/60); non-CAS cervical procedures not specified. D4: Thrombolysis and stenting administered as clinically indicated; antiplatelet and other management details unavailable. D5: No arm/subgroup outcome-specific follow-up denominators or missing-data methods. D6: Three-month mRS named; assessor blinding not described; no source sICH outcome definition. D7: P values and qualitative ordinal-model conclusion only; no adjusted OR/CI or full mRS distribution. Prespecified analysis plan unavailable.	Source evidence recorded; available abstract is insufficient for a complete result-specific seven-domain assessment. No final rating assigned.	Table S5 report citation; Table S8h source locator.
F22 / F16	D1: Actual stenting was nonrandomized; unadjusted comparison, with sex/aetiology imbalance and clinical/accessibility-based selection. The reported mRS regression omits stenting. D2: Consecutive cohort retains all 70 EVT attempts, including 10 aborted procedures. D3: C37/D33 objectively recorded; A/B not separable. D4: Usual-care antiplatelets and subsequent management. D5: Three-month loss C1/37, D2/33; acute reperfusion/HT denominators complete in Table 3. D6: Neurologist-assessed mRS and ECASS II imaging HT; masking not documented. D7: No accessible prospective analysis plan. Possible TITAN overlap prevents independent pooling of this report; risk grades are assessed separately.	Four result-level rows cover mRS, mortality, TICI, and the two HT time points grouped together.	Fahed 2016, full report PDF pp2–7 / printed pp299–304; Table 3 (p302); doi:10.1159/000444068.

Family / report IDs	Source-supported information relevant to bias	Assessment coverage	Source
F23 / D00445	C/D treatment effects are reported in Table 3 without a documented adjusted model. Operator/anatomical treatment selection, accessibility restriction and outcome-specific missingness are addressed in the result-level assessment rows below.	The result-level judgements are provided below. Results are contextual only because raw arm counts and adjustment covariates are unavailable.	Table S5 report citation; Table S8h source locator.
F27 / D01132	Retrospective nonrandomized selection; comparator procedural history and outcome threshold not stated; baseline comparability asserted but no adjusted cervical-treatment effect provided. Formal ROBINS-I domains remain unassessed.	Source evidence recorded; available abstract is insufficient for a complete result-specific seven-domain assessment. No final rating assigned.	Table S5 report citation; Table S8h source locator.
F30 / D00320	C/D counts and adjusted models are available. The 118 missing-CAS exclusions are assessed under D5; treatment selection under D1. Table 1 co-primary counts are presented descriptively. Shared Careggi centre and recruitment periods indicate possible overlap with the retained TITAN report; its extent is unknown. Source discrepancies remain explicit.	The continued table presents result-level risk-of-bias judgements.	Table S5 report citation; Table S8h source locator.
F32 / D01000, D00943	D01000: Nonrandom treatment selection; measured baseline imbalance (AF, NIHSS, time and sex); regression and propensity matching mentioned; no adjusted treatment OR/CI given; outcome denominators and sICH definition missing. No bias rating assigned.<br>D00943: Retrospective nonrandomized treatment selection; IPSWR applied; adjustment variables are not completely listed, outcome denominators/definitions incompletely reported; multiplicity correction explicitly used. No ROBINS-I rating assigned.	Source evidence recorded; available abstract is insufficient for a complete result-specific seven-domain assessment. No final rating assigned.	Table S5 report citation; Table S8h source locator.
F33 / D00935	Nonrandomized retrospective selection; A13 contains different aetiologies; no confounder-adjusted effect, missingness or assessor masking described; conference-abstract reporting only. No rating assigned.	Source evidence recorded; available abstract is insufficient for a complete result-specific seven-domain assessment. No final rating assigned.	Table S5 report citation; Table S8h source locator.
F34 / D01439	Not applicable to the present tandem-only co-primary synthesis because target-subgroup outcomes cannot be isolated; no favourable or adverse overall rating inferred.	No eligible target-strategy comparative result is available from this report for the current network. This is not a low-risk judgment; no result-level network rating assigned.	Table S5 report citation; Table S8h source locator.

Family / report IDs	Source-supported information relevant to bias	Assessment coverage	Source
F35 / D00199	No result-specific assessment needed for the present A/B/C/D synthesis; any separate procedural-order review would have a different target comparison.	No eligible target-strategy comparative result is available from this report for the current network. This is not a low-risk judgment; no result-level network rating assigned.	Table S5 report citation; Table S8h source locator.
F36 / D00893	Small retrospective nonrandomized study; no adjusted treatment effect; outcome is discharge status; treatment-arm outcomes incompletely stratified; no formal sICH definition. No ROBINS-I rating assigned.	Source evidence recorded; available abstract is insufficient for a complete result-specific seven-domain assessment. No final rating assigned.	Table S5 report citation; Table S8h source locator.
F37 / D00869	Retrospective nonrandomized cohort; baseline variables described as collected but not tabulated; no adjustment, outcome missingness, assessor masking or numerical results reported. No formal rating assigned.	Source evidence recorded; available abstract is insufficient for a complete result-specific seven-domain assessment. No final rating assigned.	Table S5 report citation; Table S8h source locator.
F41 / D00087	Clinician-selected eCAS; eight-variable IPTW; 98 exclusions for missing critical follow-up. Central blinded 90-day mRS assessment, local unblinded imaging and one-year mRS assessment. No imputation; intended strategy and crossovers unreported.	The main report was assessed; its supplement was unavailable. Shared centres and recruitment periods prevent independent pooling alongside the selected ASSIST and IRIS reports.	Article pp. 2–5, 7 and 9–10; Figure 1, p. 5; Figure 2, p. 7.
F43 / D00029	Administrative retrospective study with nonrandom treatment choice; multivariable adjustment for demographics, severity and comorbidities; outcome ascertainment at discharge/in hospital; procedural and outcome coding detail unavailable from the abstract; reported participant total inconsistent. No result-level risk-of-bias grade is assigned.	Source evidence recorded; available abstract is insufficient for a complete result-specific seven-domain assessment. No final rating assigned.	Table S5 report citation; Table S8h source locator.
F50 / D00803	Figures 4–6 verify selected 79-person counts and allow the full 92-person initial-strategy reconstruction. The selected 79 exclude 13 later-treated patients (D2); the full 92 do not. Neither dataset separates the seven common-carotid cases by strategy and outcome, so both remain descriptive only.	The continued table presents result-level risk-of-bias judgements.	Table S5 report citation; Table S8h source locator.

This table presents source evidence for 21 families not covered in Tables S6a–S6c. D1, confounding; D2, participant selection; D3, intervention classification; D4, deviations from intended interventions; D5, missing data; D6, outcome measurement; D7, selection of the reported result. Absence of information is not evidence of low risk.

Repeated publications do not receive duplicate independent assessments. The randomized EASI assessment is reported in Table S3.

The full Fahed 2016, Garg 2021 and SECURIS 2025 main reports were assessed; Garg supplementary Tables S1–S3 were also inspected. Available conference abstracts provide limited methods, outcome-specific missingness and prospective analysis details.

Table S6d (continued). Result-level risk-of-bias judgements

Report and result	D1	D2	D3	D4	D5	D6	D7	Overall	Basis and source location
Nolan: 90-day mRS 0–2, reported OR	S	M	L	L	NI	NI	NI	Serious*	D1: stenting selected by clinician and stenosis>90%, without documented confounder control. D2: accessibility-restricted cohort. D5: seven of 62 lack 90-day mRS, with no arm-specific missingness. D6/D7: assessor details and prespecified model unavailable. Methods, Table 3 and attrition discussion.
Nolan: admission sICH, reported OR	S	M	L	L	L	M	NI	Serious*	D1/D2: same uncontrolled treatment selection and accessibility restriction. D5: inpatient N60 note supports complete ascertainment. D6: defined ECASS symptomatic PH1/PH2 without documented independent masked adjudication. D7: no prospective model specification. Methods and Tables 2–3.
Sallustio: three-month mRS 0–2, raw	S	L	L	L	S	NI	M	Serious*	D1: raw comparison leaves treatment selection and baseline NIHSS/lesion imbalance uncontrolled. D5:118/466 lack CAS status, linked partly to early registry years; mRS additionally missing C4/D15. D6: assessor method/masking unspecified. D7: declared models and null results displayed, but no time-stamped plan. PDF pp 2–5,7.
Sallustio:24h sICH, raw	S	L	L	L	S	M	NI	Serious*	D1: no control of treatment-indication and bleeding-risk differences. D5:25.3% missing CAS plus sICH missing C6/D9. D6: common 24h imaging and clinical/radiographic definition, no masked central adjudication. D7: Table 1 counts and the unadjusted ORs in Tables 2–3 are not concordant. PDF pp 2–5,7.
Sallustio: mRS 0–2, adjusted model 1	S	L	L	L	S	NI	M	Serious*	D1: imbalance-selected covariates omit important baseline domains and include procedure duration, potentially downstream of stenting. D5: missing CAS/outcomes and changing complete-case populations; model N unavailable. D6: mRS assessment details unavailable. D7: all three functional models displayed. Table 2, PDF p5; Methods p3; limitations p7.
Sallustio: mRS 0–2, adjusted model 2	S	L	L	L	S	NI	M	Serious*	D1: baseline adjustment includes age, prestroke mRS, NIHSS and ASPECTS, but omits collaterals and conditions on procedure duration/angioplasty. D5: substantial missing exposure and unclear model-specific exclusions. D6: mRS assessment details unavailable. Table 3, PDF p5; Methods p3; limitations p7.
Sallustio: mRS 0–2, adjusted model 3	S	L	L	L	S	NI	M	Serious*	D1: outcome-p-value covariate selection includes procedure time and cannot ensure total-effect confounding control. D5: substantial missing exposure and unclear complete-case N. D6: mRS assessment details unavailable. D7: selection rule and all three null functional models displayed. Table 4 footnote b, PDF p5; Methods p3; limitations p7.
Sallustio:24h sICH, adjusted model 3	S	L	L	L	S	M	NI	Serious*	D1: age/onset-to-puncture adjustment does not address major source-documented prognostic imbalances. D5:118 missing-CAS exclusions plus 15 missing sICH observations; model N unspecified. D6: common 24h definition without masked adjudication. D7: raw/adjusted dataset discrepancies unresolved. Table 4 footnote h, PDF p5; pp 3–4,7.

Report and result	D1	D2	D3	D4	D5	D6	D7	Overall	Basis and source location
Wichianrat: mRS 0–2, published 79	S	S	L	L	L	NI	M	Serious*	D1: explicitly unadjusted clinician-selected comparison across 2010–2023. D2:13 patients removed according to later carotid treatment and recovery. D5: all retained outcomes accounted for. D6: mRS assessment/masking unspecified. D7: endpoint and null comparison reported, no prospective plan. PDF pp 3–5,10–11,14,17.
Wichianrat: mRS 0–2, reconstructed 92	S	L	L	L	L	NI	M	Serious*	D1: full-cohort reconstruction does not control treatment-indication/era confounding. D2/D5: retaining all 92 removes later-treatment selection and accounts for all mRS outcomes across Figures 4/6 and Table 1. D6: assessor details unavailable. D7: labelled baseline-policy reconstruction includes all source groups. Seven CCA cases affect eligibility separately. PDF pp 3–7,11,14,17.
Wichianrat: sICH, published 79	S	S	L	L	L	M	M	Serious*	D1: uncontrolled indication/era confounding. D2: inclusion depends on later treatment, which may depend on early bleeding or recovery. D5: Figure 5 accounts for all 79 retained cases. D6: Heidelberg/NIHSS definition and 24h CT, without masked adjudication. D7: null comparisons reported, no prospective plan. PDF pp 3–5,10,14,17.
Wichianrat: sICH, reconstructed 92	S	L	L	L	L	M	M	Serious*	D1: uncontrolled treatment-indication/era confounding persists. D2/D5: all 92 retained and all four events accounted for; no exclusion based on future treatment. D6: defined imaging/clinical criteria without masked adjudication or exact final observation cutoff. D7: all component groups included. CCA eligibility remains separate. PDF pp 3–7,10,14,17.
Fahed 2016 [F16]: three-month mRS 0–2, C/D	S	L	L	L	M	NI	NI	S*	D1 S: Unadjusted stenting comparison, with clinical/accessibility-based selection and sex/aetiology imbalance. D5 M: Three-month follow-up is missing for 1/37 C and 2/33 D; reasons are unavailable. D6 NI: Follow-up neurologists are identified, but masking is unspecified. D7 NI: No accessible prospective analysis plan. Common D2–D4 evidence is in the coverage row. Source: pp299–304, Table 3 (p302).
Fahed 2016 [F16]: three-month mortality, C/D	S	L	L	L	M	L	NI	S*	D1 S: Clinical/anatomical treatment selection and baseline imbalance remain unadjusted. D5 M: Mortality uses 36/37 C and 31/33 D with three-month follow-up; missing-data reasons are unspecified. D6 L: Death is objective. D7 NI: No accessible prospective analysis plan. Common D2–D4 evidence is in the coverage row. Source: pp299–304, Table 3 (p302).
Fahed 2016 [F16]: TICI 2b–3, C/D	S	L	L	L	L	M	NI	S*	D1 S: Unadjusted strategy choice depends on accessibility, anatomy and clinical condition. D5 L: Table 3 reports reperfusion for all 37 C and 33 D. D6 M: TICI is angiographic, but independent masked assessment is not described. D7 NI: No accessible prospective analysis plan. Common D2–D4 evidence is in the coverage row. Source: pp299–304, Table 3 (p302).
Fahed 2016 [F16]: haemorrhagic transformation at 24 h / within 72 h, C/D	S	L	L	L	L	M	NI	S*	D1 S: Clinical/anatomical selection remains unadjusted; lysis and stent-associated antiplatelets may affect bleeding. D5 L: Both HT time points have complete reported denominators, C37/D33. D6 M: ECASS II imaging criteria are used; assessor masking is unspecified. D7 NI: No accessible prospective analysis plan. Common D2–D4 evidence is in the coverage row. Source: pp299–303, Tables 3–4.

Report and result	D1	D2	D3	D4	D5	D6	D7	Overall	Basis and source location
Garg 2021 [L44]: inpatient ICH/death composite, ICH and nonfatal complications, C/B	S	L	M	L	NI	M	NI	S*	D1 S: Adjusted models lack NIHSS, infarct/anatomical detail and procedural timing. D5 NI: Unweighted/model-specific denominators and missing-data handling are unavailable. D6 M: Administrative ICH/complication codes lack clinical adjudication; ICH is not sICH. D7 NI: No accessible prospective plan; selective reporting is not established. Common D2–D4 evidence is in the coverage row. Source: pp2–5, Table 1/Figure 1; supplement S1–S3.
Garg 2021 [L44]: in-hospital mortality / discharge home, C/B	S	L	M	L	NI	L	NI	S*	D1 S: Key clinical/anatomical confounders remain unavailable; no overall separate adjusted mortality OR is reported. D5 NI: Unweighted/model-specific denominators and missing-data handling are unspecified. D6 L: Death and discharge destination are objective administrative outcomes. D7 NI: No accessible prospective analysis plan. Common D2–D4 evidence is in the coverage row. Source: pp2–5, Table 1/Figure 1.
Garg 2021 [L44]: non-elective 30-day readmission, C/B	S	S	M	L	NI	M	NI	S*	D1 S: Clinical confounding and HR-specific adjustment remain unresolved. D2 S: Discharge-survivor selection conditions on a post-treatment event for the starting-strategy target. D5 NI: Exclusions are stated, but residual missingness/unweighted denominators are unclear. D6 M: Administrative readmission has limited cross-state capture. D7 NI: No prospective plan; a 90-day caption conflicts with 30-day methods/results. D3–D4: See coverage. Source: pp2–5, Figures 2–3.
SECURIS: 90-day mRS/function	S	L	L	NI	S	L	NI	S*	D1: clinician-selected CAS; D2: consecutive registry; D3: recorded procedure; D4: intended strategy/crossovers unreported; D5: 98 follow-up exclusions; D6: central blinded mRS; D7: no verified analysis plan. Article pp. 3–5 and 10.
SECURIS: 90-day mortality	S	L	L	NI	S	L	NI	S*	D1: clinician-selected CAS; D2: consecutive registry; D3: recorded procedure; D4: intended strategy/crossovers unreported; D5: 98 follow-up exclusions; D6: objective death outcome; D7: no verified analysis plan. Article pp. 3–5, 7 and 10.
SECURIS: one-year mRS/function	S	L	L	NI	S	M	NI	S*	D1: clinician-selected CAS; D2: consecutive registry; D3: recorded procedure; D4: intended strategy/crossovers unreported; D5: 98 initial exclusions plus one-year losses of 24/344 and 53/234; D6: unblinded assessment; D7: no verified analysis plan. Article pp. 3–5, 7 and 10.
SECURIS: reperfusion/relevant HT	S	L	L	NI	S	M	NI	S*	D1: treatment selection and possible reverse causality; D2: consecutive registry; D3: recorded procedure; D4: intended strategy/crossovers unreported; D5: follow-up exclusions and incomplete outcome data; D6: local unblinded imaging; D7: no verified analysis plan. Article pp. 3–6 and 9–10.

L, low; M, moderate; S, serious; C, critical; NI, information insufficient for a domain judgement. *At least serious: insufficient information in other domains could support a higher overall risk. These 23 result-level assessments do not determine statistical weights or the strict-network CINeMA judgements.

Target: the effect of starting the acute cervical strategy under usual care. D3 reflects objective index-stent classification. Usual-care antiplatelets or clinically indicated later treatment are not automatically D4 deviations. Missing CAS information is handled in D5; exclusion according to later treatment in D2, without duplicate grading of the same mechanism. Wichianrat judgments describe the source cohorts; their outcomes are not quantitatively pooled because ICA-only results cannot be isolated.

Framework: ROBINS-I 2016 seven-domain result-level approach, consistent with the manuscript's ROBINS-I-aligned description; this is not the full signalling-question instrument or ROBINS-I V2. Guidance: Sterne et al., BMJ 2016; 355:i4919, doi:10.1136/bmj.i4919; Cochrane Handbook Chapter 25. Complete primary citations are in Table S5 and source outcome locations in Table S8h.

Table S7. Individual-study event counts and effects in the strict A/B/C network

Study / report	Outcome / time	Nodes	First strategy, events/N	Second strategy, events/N	OR (95% CI)	Count provenance
Dorado 2017 [D01314]	mRS 0–2 at 90 days	C vs B	12/25	11/32	1.76 (0.60–5.14)	Secondary-source cross-check
Eker 2018 [D00589]	mRS 0–2 at 90 days	B vs A	3/8	9/14	0.33 (0.06–2.02)	Reported counts
Eker 2018 [D00589]	mRS 0–2 at 90 days	C vs A	38/96	9/14	0.36 (0.11–1.17)	Reported counts
Eker 2018 [D00589]	mRS 0–2 at 90 days	C vs B	38/96	3/8	1.09 (0.25–4.84)	Reported counts
Faybushevich 2025 [D01509]	mRS 0–2 at 90 days	C vs A	31/48	19/46	2.59 (1.13–5.96)	Reported counts
Gigliotti 2021 [D00393]	mRS 0–2 at 90 days	C vs B	13/26	27/66	1.44 (0.58–3.60)	Reported counts
Han 2026 (preprint) [D00806]	mRS 0–2 at 90 days	C vs B	38/74	20/38	0.95 (0.43–2.08)	Reported counts
Jadhav 2019 [L48]	mRS 0–2 at 90 days	C vs B	50/73	15/41	3.77 (1.69–8.43)	Secondary-source cross-check
Kalmar 2021 [D00399]	mRS 0–2 at 90 days	C vs B	37/68	15/29	1.11 (0.47–2.66)	Reported counts
Kang 2019 [D00577]	mRS 0–2 at 90 days	C vs B	25/40	13/22	1.15 (0.40–3.34)	Secondary-source cross-check
Kim 2020 [D00581]	mRS 0–2 at 90 days	C vs B	36/56	5/19	5.04 (1.58–16.05)	Reported counts
Li 2018 [D00632]	mRS 0–2 at 90 days	C vs B	12/19	9/18	1.71 (0.46–6.37)	Reported counts
Li 2021 (matched) [D00388]	mRS 0–2 at 90 days	C vs B	13/22	12/22	1.20 (0.36–3.97)	Reported counts
Marnat 2016 [F15]	mRS 0–2 at 90 days	C vs A	2/5	11/15	0.24 (0.03–2.03)	Reported counts
Papanagiotou 2018 [D00621]	mRS 0–2 at 90 days	B vs A	21/52	45/108	0.95 (0.48–1.86)	Reported counts
Papanagiotou 2018 [D00621]	mRS 0–2 at 90 days	C vs A	177/322	45/108	1.71 (1.10–2.66)	Reported counts
Papanagiotou 2018 [D00621]	mRS 0–2 at 90 days	C vs B	177/322	21/52	1.80 (0.99–3.27)	Reported counts
Yu 2023 [D00305]	mRS 0–2 at 90 days	C vs B	43/64	15/34	2.59 (1.10–6.10)	Counts uniquely recovered from reported rounding
Özdemir 2025 [D00124]	mRS 0–2 at 90 days	C vs A	23/35	10/19	1.73 (0.55–5.39)	Reported counts
Dorado 2017 [D01314]	sICH; 24-72 hours or index admission	C vs B	3/28	1/40	4.68 (0.46–47.54)	Secondary-source cross-check
Eker 2018 [D00589]	sICH; 24-72 hours or index admission	B vs A	0/9	1/14	0.47 (0.02–12.93)	Reported counts

Study / report	Outcome / time	Nodes	First strategy, events/N	Second strategy, events/N	OR (95% CI)	Count provenance
Eker 2018 [D00589]	sICH; 24-72 hours or index admission	C vs A	12/98	1/14	1.81 (0.22–15.14)	Reported counts
Eker 2018 [D00589]	sICH; 24-72 hours or index admission	C vs B	12/98	0/9	2.75 (0.15–50.15)	Reported counts
Faybushevich 2025 [D01509]	sICH; 24-72 hours or index admission	C vs A	3/48	2/46	1.47 (0.23–9.21)	Reported counts
Gigliotti 2021 [D00393]	sICH; 24-72 hours or index admission	C vs B	3/26	10/66	0.73 (0.18–2.90)	Reported counts
Han 2026 (preprint) [D00806]	sICH; 24-72 hours or index admission	C vs B	10/74	8/38	0.59 (0.21–1.63)	Reported counts
Jadhav 2019 [L48]	sICH; 24-72 hours or index admission	C vs B	2/69	1/38	1.10 (0.10–12.59)	Secondary-source cross-check
Kalmar 2021 [D00399]	sICH; 24-72 hours or index admission	C vs B	6/72	1/29	2.55 (0.29–22.13)	Reported counts
Kang 2019 [D00577]	sICH; 24-72 hours or index admission	C vs B	5/40	1/22	3.00 (0.33–27.46)	Secondary-source cross-check
Kim 2020 [D00581]	sICH; 24-72 hours or index admission	C vs B	6/56	3/19	0.64 (0.14–2.86)	Reported counts
Li 2018 [D00632]	sICH; 24-72 hours or index admission	C vs B	1/19	1/18	0.94 (0.05–16.33)	Reported counts
Li 2021 (matched) [D00388]	sICH; 24-72 hours or index admission	C vs B	2/22	3/22	0.63 (0.10–4.22)	Reported counts
Marnat 2016 [F15]	sICH; 24-72 hours or index admission	C vs A	1/5	0/15	10.33 (0.36–299.97)	Reported counts
Papanagiotou 2018 [D00621]	sICH; 24-72 hours or index admission	B vs A	0/52	5/108	0.18 (0.01–3.30)	Reported counts
Papanagiotou 2018 [D00621]	sICH; 24-72 hours or index admission	C vs A	20/322	5/108	1.36 (0.50–3.73)	Reported counts
Papanagiotou 2018 [D00621]	sICH; 24-72 hours or index admission	C vs B	20/322	0/52	7.12 (0.42–119.46)	Reported counts

Study / report	Outcome / time	Nodes	First strategy, events/N	Second strategy, events/N	OR (95% CI)	Count provenance
Wallocha 2019 [D00580]	sICH; 24-72 hours or index admission	C vs B	7/149	1/14	0.64 (0.07–5.62)	Reported counts
Yu 2023 [D00305]	sICH; 24-72 hours or index admission	C vs B	4/64	2/34	1.07 (0.19–6.14)	Reported counts

A: no targeted acute cervical revascularization; B: balloon angioplasty alone; C: emergent cervical carotid stenting. Denominators are outcome-specific evaluable participants. Functional outcomes include 15 cohorts and 1,556 participants; sICH includes 15 cohorts and 1,676 participants.

Three-arm studies are shown as three direct contrasts; shared arms are not summed repeatedly. Their within-study correlation is handled in the network analysis. The table contains 19 direct contrasts per outcome, not 19 independent cohorts.

Study-level ORs compare the first-listed with the second-listed strategy. Wald 95% CIs are calculated on the log-OR scale. A correction of 0.5 is added to all four cells only when at least one cell is zero. All displayed direct estimates use the same arm-level event counts and this method.

An OR above 1 favours the first-listed strategy for functional independence but indicates higher bleeding odds for sICH. Count derivations and secondary-source cross-checks are identified in the last column.

Table S8a. Source-reported adjusted effects by analysis role

Study / report	Outcome	Source group counts, events/N	Adjusted OR (95% CI)	Model / recorded covariates	Role in the review
Madden 2023 [D00989]	Functional independence, mRS 0-2; 90 days	C: 70%; D: 46%; outcome events/N NR (enrolled C=30, D=66)	2.50 (0.90–6.80)	Adjusted model reported by study; model N=NR; baseline NIHSS	Contextual adjusted evidence
Sousa 2025 [D00141]	Functional independence, mRS 0-2; 90 days	C: 84/130; D: 86/144	1.23 (0.82–1.86)	primary age/NIHSS-adjusted; model N=270; age and NIHSS	Sensitivity only: dissection-restricted population
Sousa 2025 [D00141]	Symptomatic intracranial haemorrhage; 24 hours	C: 11/150; D: 14/178	0.95 (0.41–2.20)	primary age/NIHSS-adjusted; model N=324; age and NIHSS	Sensitivity only: dissection-restricted population
Deliktas, 2025 [D00185]	Symptomatic intracranial haemorrhage; imaging within 24 hours	C: 10/NR (18.5%); D: 20/NR (15.6%)	1.45 (0.60–3.48)	Adjusted model reported by study; model N=NR	Contextual adjusted evidence
Romoli, 2026 [D00067]	mRS 0–2 or unchanged from prestroke, 90 days	C: 1188/2522; D: 535/1531	1.30 (1.13–1.51)	estimand 1 IPTW; model N=NR	Dominant adjusted cohort; report separately because of overlap

Study / report	Outcome	Source group counts, events/N	Adjusted OR (95% CI)	Model / recorded covariates	Role in the review
Romoli, 2026 [D00067]	Symptomatic intracranial haemorrhage; Post-treatment safety window (study-defined)	C: 280/2522; D: 143/1531	1.21 (0.93–1.56)	estimand 1 IPTW; model N=NR	Dominant adjusted cohort; report separately because of overlap
IRIS 2026 [D00081]	Functional independence, mRS 0-2; 90 days	C: 62/112; D: 99/216	1.28 (0.75–2.20)	adjusted; model N=NR	Core non-CERES adjusted evidence
IRIS 2026 [D00081]	Symptomatic intracranial haemorrhage; Post-treatment safety window (study-defined)	C: 7/112; D: 8/215	2.09 (0.78–5.59)	adjusted; model N=NR	Core non-CERES adjusted evidence
Rosales 2026 [D00808]	Functional independence, mRS 0-2; 90 days	C: 32/62; D: 8/25	1.95 (0.62–6.17)	adjusted for age, sex and admission NIHSS; model N=NR	Sensitivity only: preprint
Rosales 2026 [D00808]	Symptomatic intracranial haemorrhage; 48 hours	C: 19/73; D: 9/37	1.17 (0.44–3.06)	adjusted for age, sex and admission NIHSS; model N=NR	Sensitivity only: preprint
ESCAPE-NA1 2022 [D00403]	Functional independence, mRS 0-2; 90 days	C: 47/62; D: 35/53	2.00 (0.80–5.10)	multivariable logistic regression; model N=115; age, sex, baseline NIHSS, ASPECTS, intracranial occlusion location, and nerinetide-by-alteplase interaction	Core non-CERES adjusted evidence
Beltrán-Guaquetá 2026 [D00030]	Functional independence, mRS 0-2; variable follow-up	C: 9/21; D: 8/45	2.07 (0.56–7.75)	final multivariable logistic regression; model N=66; CAS, admission NIHSS and atrial fibrillation were considered; final table retained CAS and NIHSS	Sensitivity only: nonstandard follow-up
Farooqui 2023 [D00285]	Functional independence, mRS 0-2; 90 days	Matched C: NR; matched D: NR	2.10 (1.43–3.12)	propensity-score matched; model N=NR; matching on sex, race, admission NIHSS, periprocedural antiplatelets, and cervical ICA stenosis	Sensitivity only: preferred PSM adjusts for periprocedural antiplatelets

Study / report	Outcome	Source group counts, events/N	Adjusted OR (95% CI)	Model / recorded covariates	Role in the review
Farooqui 2023 [D00285]	Symptomatic intracranial haemorrhage; Post-treatment safety window (study-defined)	Matched C: NR; matched D: NR	1.40 (0.40–4.60)	propensity-score matched; model N=NR; same propensity-score variables	Sensitivity only: preferred PSM adjusts for periprocedural antiplatelets

The 14 adjusted estimates used in the review are shown with their roles in the core, contextual or sensitivity analyses. Alternative models are not presented as independent studies.

D is the source-defined mixed nonstenting comparator and is not decomposed into A and B. CERES is reported separately because of potential cohort overlap. Its functional endpoint includes mRS 0–2 or no worsening from prestroke.

Farooqui matched-arm events and outcome-specific denominators were not reported in the main article or either supplement. eFigure 2 (Supplement 1, page 8) reports only model estimates, CIs and P values. Unmatched counts are not substituted. Matching includes antiplatelet treatment and remains sensitivity-only evidence.

Other displayed counts are unweighted source outcome data, not adjusted-model or weighted pseudo-population sample sizes. NR means not reported or not uniquely verifiable; model N is never replaced by total enrollment.

The large-core ETIS sICH table gives event counts and percentages but not outcome-specific group denominators. The estimate is presented contextually; missing denominators are not back-calculated.

Table S8b. Additional co-primary event-count evidence and the randomized pilot

Study / report	Outcome / time	C, events/N	D, events/N	Unadjusted OR (95% CI)	Analysis role
EASI pilot [D00540]	mRS 0–2 at 90 days; 90 days	8/13	7/11	0.91 (0.17–4.81)	Randomized pilot; also included in four-node sensitivity
IRIS 2026 [D00081]	mRS 0–2 at 90 days; 90 days	62/112	99/216	1.47 (0.93–2.32)	Included in four-node sensitivity
ASSIST 2026 [D00027]	mRS 0–2 at 90 days; 90 days	56/79	45/92	2.54 (1.35–4.80)	Included in four-node sensitivity
ESCAPE-NA1 2022 [D00403]	mRS 0–2 at 90 days; 90 days	47/62	35/53	1.61 (0.71–3.63)	Included in four-node sensitivity
EASI pilot [D00540]	sICH; 24-hour brain imaging (ECASS-2)	0/13	1/11	0.26 (0.01–7.03)	Randomized pilot; also included in four-node sensitivity
Kojima 2023 [D00283]	sICH; 14 days after procedure	4/36	0/14	4.02 (0.20–79.58)	Included in four-node sensitivity
IRIS 2026 [D00081]	sICH; post-treatment safety follow-up	7/112	8/215	1.73 (0.61–4.89)	Included in four-node sensitivity
ASSIST 2026 [D00027]	sICH; 24 hours	2/83	3/100	0.80 (0.13–4.89)	Included in four-node sensitivity

Study / report	Outcome / time	C, events/N	D, events/N	Unadjusted OR (95% CI)	Analysis role
ESCAPE-NA1 2022 [D00403]	sICH; 24-hour follow-up imaging	2/62	3/53	0.56 (0.09–3.46)	Included in four-node sensitivity

These nine outcome-specific results supplement the strict-network data in the overlap-restricted four-node sensitivity analysis. Together with Table S7, they comprise 19 cohorts and 2,194 participants for functional independence and 20 cohorts and 2,375 participants for sICH. Additional source results are presented descriptively in Table S8h.

The 24-patient EASI pilot randomized the acute stenting policy; D comprises two balloon-angioplasty and nine no-targeted-treatment participants. EASI is also shown separately as randomized evidence. The other displayed source ORs are derived from arm-level event counts using the Table S7 formula.

Adjusted estimates in Table S8a and the unadjusted effects here address different estimands and are not pooled together.

Kojima defined sICH as occurring within 14 days after the procedure (source PDF p3; printed p e210).

Table S8c. Individual-study secondary and contextual binary outcomes

Study / report	Outcome / time	Nodes	C, events/N	Comparator, events/N	Descriptive OR (95% CI)	Provenance / limitation
ASSIST 2026 [D00027]	Mortality; 90 days	C vs D	4/83	10/100	0.46 (0.14–1.51)	Outcome-specific evaluable cohort; reported Source: PDF p8; PDF p8, Table 3
ASSIST 2026 [D00027]	Successful reperfusion, TICI 2b-3; end of procedure	C vs D	82/82	95/99	7.77 (0.41–146.56)	Published C-versus-D outcome population; reported Source: PDF p6, Table 2
Beltrán-Guaquetá 2026 [D00030]	Excellent reperfusion, TICI 2c-3; End of procedure	C vs D	16/21	21/45	3.66 (1.14–11.70)	Outcome-specific evaluable cohort; reported; variable follow-up, not a 90-day estimate Source: PDF pages 2-7, Tables 1-2
Beltrán-Guaquetá 2026 [D00030]	Mortality; variable follow-up	C vs D	4/21	23/45	0.23 (0.07–0.77)	Outcome-specific evaluable cohort; reported; variable follow-up, not a 90-day estimate Source: PDF p6; PDF p6, Table 2
Beltrán-Guaquetá 2026 [D00030]	Successful reperfusion, TICI 2b-3; end of procedure	C vs D	21/21	32/45	17.86 (1.01–316.53)	Outcome-specific evaluable cohort; reported; variable follow-up, not a 90-day estimate Source: PDF p6; PDF p6, Table 2
Deliktas, 2025 [D00185]	Any intracranial hemorrhage	C vs D	37/NR (68.5%)	73/NR (56.6%)	NE: denominator not reported	Events and percentages reported; evaluable arm denominators not printed. Source adjusted estimate shown separately; no reverse-inference of integer denominators. Source: PDF p3, Table 1

Study / report	Outcome / time	Nodes	C, events/N	Comparator, events/N	Descriptive OR (95% CI)	Provenance / limitation
Deliktas, 2025 [D00185]	Carotid patency; day 1	C vs D	32/NR (86.5%)	51/NR (68.9%)	NE: denominator not reported	Events and percentages reported; evaluable arm denominators not printed. Source adjusted estimate shown separately; no reverse-inference of integer denominators. Source: PDF p3, Table 1
Deliktas, 2025 [D00185]	Decompressive craniectomy	C vs D	4/NR (9.1%)	7/NR (6.2%)	NE: denominator not reported	Events and percentages reported; evaluable arm denominators not printed. Source adjusted estimate shown separately; no reverse-inference of integer denominators. Source: PDF p3, Table 1
Deliktas, 2025 [D00185]	Early neurological improvement	C vs D	20/55	50/141	1.04 (0.54–1.99)	Descriptive result, not pooled. Source: PDF p3, Table 1
Deliktas, 2025 [D00185]	Mortality; 90 days	C vs D	13/55	41/141	0.75 (0.37–1.55)	Descriptive result, not pooled. Source: PDF p3, Table 1
Deliktas, 2025 [D00185]	Parenchymal hematoma	C vs D	11/NR (24.4%)	21/NR (19.6%)	NE: denominator not reported	Events and percentages reported; evaluable arm denominators not printed. Source adjusted estimate shown separately; no reverse-inference of integer denominators. Source: PDF p3, Table 1
Deliktas, 2025 [D00185]	mRS 0–1; 90 days	C vs D	6/55	19/141	0.79 (0.30–2.09)	Descriptive result, not pooled. Source: PDF p3, Table 1
Deliktas, 2025 [D00185]	mRS 0–2; 90 days (contextual source, not pooled)	C vs D	16/55	37/141	1.15 (0.58–2.30)	Descriptive result, not pooled. Source: PDF p3, Table 1
Deliktas, 2025 [D00185]	mRS 0–3; 90 days	C vs D	29/55	58/141	1.60 (0.85–2.99)	Descriptive result, not pooled. Source: PDF p3, Table 1
Farooqui 2023 [D00285]	Intracranial or extracranial procedural complications	C vs D	18/NR (6.1%)	13/NR (6.4%)	NE: source discrepancy	Reported arm counts sum to 31, whereas total is 34; arm evaluable denominators are not given. No derived effect. Source: Main PDF p7, Table 2

Study / report	Outcome / time	Nodes	C, events/N	Comparator, events/N	Descriptive OR (95% CI)	Provenance / limitation
Farooqui 2023 [D00285]	Mortality; 90 days	C vs D	52/325	44/196	NE: source discrepancy	Unmatched outcomes. Main Table 2 (PDF p7) reports deaths C=52, D=44 but total=99, repeated in Supplement 1 eTable 2. Figure 2 (PDF p8) mRS category counts independently sum to outcome N=325 and 196 and deaths 52/44. The sum is 96, not 99; the discrepancy is retained and no OR is derived. Source: Main PDF pp7–8, Table 2 and Figure 2; Supplement 1 PDF p3, eTable 2
Farooqui 2023 [D00285]	Periprocedural hemodynamic impairment	C vs D	43/NR (11.8%)	13/NR (5.0%)	NE: denominator not reported	Unmatched source counts; arm outcome-specific N not printed. Footnote components: bradycardia requiring atropine 17 vs 4, hypertension requiring labetalol 1 vs 1, hypotension requiring vasopressors 25 vs 8. No outcome denominator is assumed from enrollment. Source: Main PDF p7, Table 2 and footnote f
Farooqui 2023 [D00285]	Successful reperfusion, TICI 2b-3; end of procedure	C vs D	306/338	171/212	2.29 (1.39–3.78)	Unmatched source counts. The reconstructed denominators 338/212 were uniquely recovered from reported rounding and sum to 550, also reported for this outcome in Supplement 1 eTable 1. Main Table 2 sample-size column instead says 548. This source discrepancy is reported; the descriptive arm-based calculation is not substituted into the propensity-matched analysis. Source: Main PDF p7, Table 2; Supplement 1 PDF p2, eTable 1

Study / report	Outcome / time	Nodes	C, events/N	Comparator, events/N	Descriptive OR (95% CI)	Provenance / limitation
Farooqui 2023 [D00285]	mRS 0–2; discharge	C vs D	74/256 (arm N uniquely reconstructed)	40/187 (arm N uniquely reconstructed)	NE: source OR differs from reconstructed counts	Unmatched discharge endpoint. Arm N256/187 uniquely follows the printed events 74/40, percentages 28.9%/21.4% rounded to one decimal, and explicit total N443. Arm Ns are reconstructed, not directly printed; PSM counts/N remain unavailable. Source-reported unadjusted OR 1.6 differs from the reconstructed 2×2 OR at its printed precision; no effect is derived or substituted. Source: main PDF p7, Table 2.
Faybushevich 2025 [D01509]	Mortality; 90 days	C vs A	5/48	9/46	0.48 (0.15–1.55)	Outcome-specific evaluable cohort; reported Source: Printed page 92, Table 3
Faybushevich 2025 [D01509]	Successful reperfusion, TICI 2b-3; End of procedure	C vs A	48/48	46/46	1.04 (0.02–53.66)	Outcome-specific evaluable cohort; reported Source: PDF page 4, Table 2
Gigliotti 2021 [D00393]	Mortality; 90 days	C vs B	1/26	8/66	0.29 (0.03–2.44)	Outcome-specific evaluable cohort; reported Source: PDF page 4, Table 2
Gigliotti 2021 [D00393]	Successful reperfusion, TICI 2b-3; End of procedure	C vs B	26/26	66/66	0.40 (0.01–20.61)	Outcome-specific evaluable cohort; reported Source: PDF page 3, Table 1 continued and Methods inclusion criterion
Han 2026 (preprint) [D00806]	Mortality; 90 days	C vs B	7/74	2/38	1.88 (0.37–9.53)	Outcome-specific evaluable cohort; reported Source: PDF page 10, Table 3 continued and Figure 2
Han 2026 (preprint) [D00806]	Successful reperfusion, TICI 2b-3; End of procedure	C vs B	66/74	34/38	0.97 (0.27–3.45)	Outcome-specific evaluable cohort; denominators uniquely reconstructed from the source Source: PDF page 9, Table 3
IRIS 2026 [D00081]	Any intracranial hemorrhage	C vs D	49/112	72/207	1.46 (0.91–2.33)	Descriptive result, not pooled. Source: PDF p8, Table 2 and missing-data footnotes

Study / report	Outcome / time	Nodes	C, events/N	Comparator, events/N	Descriptive OR (95% CI)	Provenance / limitation
IRIS 2026 [D00081]	Any procedural complication	C vs D	20/96	24/155	1.44 (0.74–2.77)	Descriptive result, not pooled. Source: PDF p5 / printed p30, Results
IRIS 2026 [D00081]	Embolization to a new territory	C vs D	7/106	6/184	2.10 (0.69–6.41)	Descriptive result, not pooled. Source: PDF p9, Table 3
IRIS 2026 [D00081]	Excellent reperfusion, eTICI 2c–3	C vs D	54/111	102/204	0.95 (0.60–1.50)	Descriptive result, not pooled. Source: PDF p9 / printed p34, Table 3
IRIS 2026 [D00081]	Procedural dissection	C vs D	0/102	4/182	0.19 (0.01–3.63)	Descriptive result, not pooled. Source: PDF p9, Table 3
IRIS 2026 [D00081]	Procedural perforation	C vs D	2/103	3/182	1.18 (0.19–7.19)	Descriptive result, not pooled. Source: PDF p9, Table 3
IRIS 2026 [D00081]	Recurrent stroke during follow-up	C vs D	1/68	3/113	0.55 (0.06–5.37)	Descriptive result, not pooled. Source: PDF p9, Table 3
IRIS 2026 [D00081]	Successful reperfusion, TICI 2b-3; end of procedure	C vs D	96/111	173/204	1.15 (0.59–2.23)	Published C-versus-D outcome population; reported Source: PDF p9 / printed p34, Table 3
IRIS 2026 [D00081]	mRS 0–1; 90 days	C vs D	42/112	48/216	2.10 (1.27–3.46)	Descriptive result, not pooled. Source: PDF p8 / printed p33, Table 2 and missing-data footnotes
IRIS 2026 [D00081]	mRS 5–6; 90 days	C vs D	20/112	60/216	0.57 (0.32–1.00)	Descriptive result, not pooled. Source: PDF p8 / printed p33, Table 2 and missing-data footnotes
Rosales 2026 [D00808]	Early neurological deterioration; 24 hours	C vs D	25/74	16/37	0.67 (0.30–1.50)	Descriptive result, not pooled. Source: PDF p14, Table 2
Rosales 2026 [D00808]	In-hospital mortality	C vs D	20/72	12/37	0.80 (0.34–1.89)	Descriptive result, not pooled. Source: PDF p14, Table 2
Rosales 2026 [D00808]	Mortality; 90 days	C vs D	22/62	12/25	0.60 (0.23–1.53)	Outcome-specific evaluable cohort; reported Source: PDF p14; PDF p14, Table 2
Rosales 2026 [D00808]	Successful reperfusion, TICI 2b-3; end of procedure	C vs D	72/74	29/37	9.93 (1.99–49.60)	Published C-versus-D outcome population; reported Source: PDF p14, Table 2

Study / report	Outcome / time	Nodes	C, events/N	Comparator, events/N	Descriptive OR (95% CI)	Provenance / limitation
Rosales 2026 [D00808]	mRS 0–1; 90 days	C vs D	26/62	3/25	5.30 (1.43–19.58)	Descriptive result, not pooled. Source: PDF p14, Table 2
Kalmar 2021 [D00399]	Mortality; 90 days	C vs B	16/68	4/29	1.92 (0.58–6.35)	Outcome-specific evaluable cohort; reported Source: PDF page 6, Table 2
Kalmar 2021 [D00399]	Successful reperfusion, TICI 2b-3; End of procedure	C vs B	62/72	22/29	1.97 (0.67–5.82)	Outcome-specific evaluable cohort; denominators uniquely reconstructed from reported rounding Source: PDF pages 4-6, Results/Table 2
Kim 2020 [D00581]	Mortality; 90 days	C vs B	4/56	6/19	0.17 (0.04–0.68)	Outcome-specific evaluable cohort; reported Source: PDF page 5, Table 2
Kim 2020 [D00581]	Successful reperfusion, TICI 2b-3; End of procedure	C vs B	53/56	12/19	10.31 (2.32–45.75)	Outcome-specific evaluable cohort; reported Source: PDF page 5, Table 2
Li 2021 (unmatched) [D00388]	Mortality; 90 days	C vs B	3/30	6/45	0.72 (0.17–3.14)	Unmatched full cohort; reported; these secondary outcomes use the unmatched cohort, not the 22-per-arm matched co-primary sample Source: PDF page 4, Table 3, before matching
Li 2021 (unmatched) [D00388]	Successful reperfusion, TICI 2b-3; End of procedure	C vs B	22/30	32/45	1.12 (0.40–3.14)	Unmatched full cohort; reported; these secondary outcomes use the unmatched cohort, not the 22-per-arm matched co-primary sample Source: PDF page 4, Table 2, before matching
Marko 2022 [D00403]	Any angiographic procedural complication	C vs D	5/62	2/53	2.24 (0.42–12.04)	Tandem stented versus tandem nonstented patients; not the separate tandem-versus-nontandem comparison Source: Supplement PDF p3, Table II

Study / report	Outcome / time	Nodes	C, events/N	Comparator, events/N	Descriptive OR (95% CI)	Provenance / limitation
Marko 2022 [D00403]	Excellent reperfusion, TICI 2c–3	C vs D	24/62	16/53	1.46 (0.67–3.18)	Tandem stented versus tandem nonstented patients; not the separate tandem-versus-nontandem comparison Source: Supplement PDF p3, Table II
Marko 2022 [D00403]	Mortality; 90 days	C vs D	4/62	6/53	0.54 (0.14–2.03)	Outcome-specific evaluable cohort; reported Source: PDF p3; PDF p3, Supplemental Table II
Marko 2022 [D00403]	Recurrent stroke during follow-up	C vs D	0/62	3/53	0.12 (0.01–2.29)	Tandem stented versus tandem nonstented patients; not the separate tandem-versus-nontandem comparison Source: Supplement PDF p3, Table II
Marko 2022 [D00403]	Successful reperfusion, TICI 2b-3; end of procedure	C vs D	61/62	36/53	28.81 (3.68–225.65)	Outcome-specific evaluable cohort; reported Source: PDF p3; PDF p3, Supplemental Table II
Romoli, 2026 [D00067]	Early neurological deterioration; within 48 hours (NIHSS increase ≥4)	C vs D	467/2522	333/1531	0.82 (0.70–0.96)	Descriptive result, not pooled. Source: PDF pp3,8, outcome definition and Table 2
Romoli, 2026 [D00067]	Successful reperfusion, TICI 2b-3; end of procedure	C vs D	2292/2522	1162/1531	3.16 (2.65–3.79)	Outcome-specific evaluable cohort; reported Source: PDF p8 / article e214528(8); PDF p8 / article e214528(8), Table 2
Romoli, 2026 [D00067]	mRS 0–1; 90 days	C vs D	733/2522	309/1531	1.62 (1.39–1.89)	Descriptive result, not pooled. Source: PDF p8, Table 2, estimand 1
Sousa 2025 [D00141]	Any intracranial hemorrhage; 24 hours	C vs D	44/150	31/178	1.97 (1.17–3.32)	Descriptive result, not pooled. Source: PDF p5, Table 2
Sousa 2025 [D00141]	Mortality; within 90 days	C vs D	6/150	11/178	0.63 (0.23–1.75)	Outcome-specific evaluable cohort; reported Source: PDF p5 / printed p812; PDF p5 / printed p812, Table 2
Sousa 2025 [D00141]	Recurrent ischemic stroke; 90 days	C vs D	8/150	10/178	0.95 (0.36–2.46)	Descriptive result, not pooled. Source: PDF p5, Table 2

Study / report	Outcome / time	Nodes	C, events/N	Comparator, events/N	Descriptive OR (95% CI)	Provenance / limitation
Sousa 2025 [D00141]	Successful reperfusion, TICI 2b-3; end of procedure	C vs D	129/144	131/171	2.63 (1.38–4.99)	Published C-versus-D outcome population; reported Source: PDF p5 / printed p812, Table 2
Sousa 2025 [D00141]	Symptomatic intracranial hemorrhage; 90 days	C vs D	13/150	14/178	1.11 (0.51–2.44)	Descriptive result, not pooled. Source: PDF p5, Table 2
Sousa 2025 [D00141]	mRS 0–1; 90 days	C vs D	51/130	51/144	1.18 (0.72–1.92)	Descriptive result, not pooled. Source: PDF p5 / printed p812, Table 2
Yu 2023 [D00305]	Mortality; 90 days	C vs B	4/64	3/34	0.69 (0.14–3.27)	Outcome-specific evaluable cohort; denominators uniquely reconstructed from reported rounding Source: Results/outcome table
Yu 2023 [D00305]	Successful reperfusion, TICI 2b-3; End of procedure	C vs B	63/64	28/34	13.50 (1.55–117.45)	Outcome-specific evaluable cohort; reported Source: PDF page 5, Table 5
Özdemir 2025 [D00124]	Hemorrhagic infarction type 1 or 2	C vs A	2/35	1/19	1.09 (0.09–12.87)	Reported source counts; nonstenting comparator is classified as node A Source: PDF p4 / printed p237, Table 2
Özdemir 2025 [D00124]	In-hospital mortality	C vs A	3/35	3/19	0.50 (0.09–2.76)	Reported source counts; nonstenting comparator is classified as node A Source: PDF p4 / printed p237, Table 2
Özdemir 2025 [D00124]	Mortality; 90 days	C vs A	3/35	6/19	0.20 (0.04–0.94)	Outcome-specific evaluable cohort; reported Source: Table 2
Özdemir 2025 [D00124]	Parenchymal hematoma type 1 or 2	C vs A	4/35	1/19	2.32 (0.24–22.41)	Reported source counts; nonstenting comparator is classified as node A Source: PDF p4 / printed p237, Table 2

Study / report	Outcome / time	Nodes	C, events/N	Comparator, events/N	Descriptive OR (95% CI)	Provenance / limitation
Özdemir 2025 [D00124]	Successful reperfusion, TICI 2b-3; End of procedure	C vs A	26/35	13/19 (Table 1); 15/19 (text)	NE: source discrepancy	Main Table 1 (PDF p4, printed p237) reports C=26/35, A=13/19; Results narrative (PDF p3, printed p236) reports A=15/19. Both are preserved as a source discrepancy; no OR is derived. Source: PDF pp3–4 / printed pp236–237, Results text and Table 1
Özdemir 2025 [D00124]	mRS 0–2; discharge	C vs A	21/35	9/19	1.67 (0.54–5.14)	Reported source counts; nonstenting comparator is classified as node A Source: PDF p4 / printed p237, Table 2

Secondary outcomes are shown for source cohorts contributing to the main, sensitivity or adjusted-context analyses. Additional outcomes were checked against the same reports; definitions and denominators remain source-specific.

Descriptive ORs and Wald 95% CIs use the Table S7 method, including its zero-cell correction. These secondary descriptive results are not pooled.

All tabulated ORs are descriptive C-versus-comparator contrasts. These secondary outcomes are presented without additional pooled models. Hazard ratios, ordinal effects and source-adjusted estimates are shown in Table S8d, not treated as interchangeable with these crude binary ORs.

NR: not reported or not uniquely verifiable. NE: effect not derived. Source inconsistencies are retained, including Farooqui mortality and procedural-complication totals and Özdemir reperfusion counts. Outcome-specific denominators are not substituted by enrollment or by a different analytic population.

IRIS missing-outcome denominators are directly obtained from its explicit Table 2 missing-data footnotes (not reconstructed from rounded percentages). CERES counts describe unweighted source groups. The ETIS mRS 0–2 result is additionally reported for completeness but does not enter the adjusted synthesis.

Li propensity-study secondary data are from the unmatched cohort; the matched co-primary sample is separately shown in Table S7. Colombia follow-up is variable and not relabelled as 90 days. Potentially overlapping cohorts are not summed.

Table S8d. Source-reported secondary effects and matched sensitivity estimates

Study / report	Outcome (C vs D)	Measure and estimate (95% CI)	Selected source model	Review role
Deliktas, 2025 [D00185]	Any intracranial hemorrhage; as labelled	OR 1.41 (0.69–2.86)	Adjusted for age, NIHSS, IV thrombolysis and onset-to-puncture time; model N NR	Descriptive; PDF p3, Table 1 and Results
Deliktas, 2025 [D00185]	Carotid patency; day 1; as labelled	OR 3.54 (1.14–11.01)	Adjusted for age, NIHSS, IV thrombolysis and onset-to-puncture time; model N NR	Descriptive; PDF p3, Table 1 and Results
Deliktas, 2025 [D00185]	Decompressive craniectomy; as labelled	OR 2.06 (0.41–10.23)	Adjusted for age, NIHSS, IV thrombolysis and onset-to-puncture time; model N NR	Descriptive; PDF p3, Table 1 and Results

Study / report	Outcome (C vs D)	Measure and estimate (95% CI)	Selected source model	Review role
Deliktas, 2025 [D00185]	Mortality; 90 days	adjusted OR 0.74 (0.33–1.68)	Adjusted model reported by study	Source primary/selected secondary estimate; descriptive, not pooled; Abstract, Results
Deliktas, 2025 [D00185]	Ordinal mRS; 90 days; as labelled	common OR 1.61 (0.88–2.95)	Adjusted for age, NIHSS, IV thrombolysis and onset-to-puncture time; model N NR	Descriptive; PDF p3, Table 1 and Results
Deliktas, 2025 [D00185]	Parenchymal hematoma; as labelled	OR 1.21 (0.49–3.02)	Adjusted for age, NIHSS, IV thrombolysis and onset-to-puncture time; model N NR	Descriptive; PDF p3, Table 1 and Results
Deliktas, 2025 [D00185]	mRS 0–1; 90 days; as labelled	OR 1.33 (0.43–4.09)	Adjusted for age, NIHSS, IV thrombolysis and onset-to-puncture time; model N NR	Descriptive; PDF p3, Table 1 and Results
Deliktas, 2025 [D00185]	mRS 0–2; 90 days; as labelled	OR 1.48 (0.65–3.35)	Adjusted for age, NIHSS, IV thrombolysis and onset-to-puncture time; model N NR	Descriptive; PDF p3, Table 1 and Results
Deliktas, 2025 [D00185]	mRS 0–3; 90 days; as labelled	OR 2.40 (1.10–5.21)	Adjusted for age, NIHSS, IV thrombolysis and onset-to-puncture time; model N NR	Descriptive; PDF p3, Table 1 and Results
Farooqui 2023 [D00285]	Mortality; 90 days	OR 0.78 (0.50–1.20)	Multivariable model after multiple imputation; adjusted for NIHSS and mTICI. Source endpoint N=521 before imputation; this is neither a matched sample size nor an explicitly stated post-imputation analytic sample size.	Descriptive, not pooled. Source: Supplement PDF p2, eTable1 and outcome-specific footnote; main Table2, PDF p7.
Farooqui 2023 [D00285]	Mortality; 90 days	OR 0.70 (0.44–1.02)	propensity-score matched	Propensity-matched sensitivity model; matched counts/N NR; descriptive only; PDF pp2-3,8, eTables 1-2 and eFigure 2
Farooqui 2023 [D00285]	Ordinal mRS; 90 days; as labelled	common OR 1.46 (1.02–2.10)	Source multivariable ordinal analysis; adjusted for last-known-well time, NIHSS, ASPECTS, center volume and reperfusion status; not a baseline-only total-effect model	Descriptive; Main PDF pp1,5, abstract and Results

Study / report	Outcome (C vs D)	Measure and estimate (95% CI)	Selected source model	Review role
Farooqui 2023 [D00285]	Successful reperfusion, TICI 2b-3; End of procedure	OR 1.70 (1.02–3.60)	Multivariable model after multiple imputation; adjusted for extracranial preprocedure stenosis and other procedures (intracranial angioplasty, intracranial stenting, or both versus other procedures). eTable1 states outcome N=550; main Table 2 instead states 548. The source sample-size discrepancy is retained.	Descriptive, not pooled. Source: Supplement PDF p2, eTable1 and outcome-specific footnote; main Table2, PDF p7.
Farooqui 2023 [D00285]	Successful reperfusion, TICI 2b-3; End of procedure	OR 2.20 (0.98–4.92)	propensity-score matched	Propensity-matched sensitivity model; matched counts/N NR; descriptive only; PDF pp2-3,8, eTables 1-2 and eFigure 2
Farooqui 2023 [D00285]	discharge mRS 0–2; as labelled	OR 1.20 (0.80–1.80)	Multivariable model after multiple imputation; adjusted for last-known-well time, NIHSS, ASPECTS and mTICI. Source endpoint N=443 before imputation; this is not a reported PSM analytic N.	Descriptive, not pooled. Source: Supplement PDF p2, eTable1 and outcome-specific footnote; main Table2, PDF p7.
Farooqui 2023 [D00285]	discharge mRS 0–2; as labelled	OR 1.70 (1.09–2.52)	propensity-score matched	Propensity-matched sensitivity model; matched counts/N NR; descriptive only; PDF pp2-3,8, eTables 1-2 and eFigure 2
IRIS 2026 [D00081]	Any intracranial haemorrhage; Post-treatment safety window (study-defined)	OR 1.30 (0.79–2.15)	adjusted	Source primary/selected secondary estimate; descriptive, not pooled; PDF pp1,8 / printed pp27,33, Abstract and Table 2

Study / report	Outcome (C vs D)	Measure and estimate (95% CI)	Selected source model	Review role
IRIS 2026 [D00081]	NIHSS at 5–7 days	SMD −0.06 (−0.32–0.19)	adjusted	Source primary/selected secondary estimate; descriptive, not pooled; PDF pp1,8 / printed pp27,33, Abstract and Table 2
IRIS 2026 [D00081]	Ordinal modified Rankin Scale at 90 days; 90 days	common OR 1.60 (1.03–2.47)	mixed-effect adjusted ordinal regression	Source primary/selected secondary estimate; descriptive, not pooled; PDF pp1,8 / printed pp27,33, Abstract and Table 2
IRIS 2026 [D00081]	Procedure duration; as labelled	standardized mean difference 0.25 (0.10–0.40)	adjusted	Source primary/selected secondary estimate; descriptive, not pooled; PDF pp1,8 / printed pp27,33, Abstract and Table 2
IRIS 2026 [D00081]	mRS 0–1; 90 days	OR 1.91 (1.05–3.46)	adjusted	Source primary/selected secondary estimate; descriptive, not pooled; PDF pp1,8 / printed pp27,33, Abstract and Table 2
IRIS 2026 [D00081]	90-day mRS 5–6; 90 days	OR 0.72 (0.38–1.35)	adjusted	Source primary/selected secondary estimate; descriptive, not pooled; PDF pp1,8 / printed pp27,33, Abstract and Table 2

Study / report	Outcome (C vs D)	Measure and estimate (95% CI)	Selected source model	Review role
Rosales 2026 [D00808]	Mortality; 90 days	OR 0.69 (0.24–2.01)	adjusted for age, sex and admission NIHSS	Source primary/selected secondary estimate; descriptive, not pooled; PDF pp7,14-15, Table 2 and Figure 1
Rosales 2026 [D00808]	Ordinal mRS; 90 days; as labelled	common OR 2.20 (0.93–5.26)	Unadjusted ordinal regression (explicit Table 2 footnote); not an adjusted model	Descriptive; PDF p14, Table 2
Rosales 2026 [D00808]	Successful reperfusion, TICI 2b-3; End of procedure	OR 13.26 (2.13–82.49)	adjusted for age, sex and admission NIHSS	Source primary/selected secondary estimate; descriptive, not pooled; PDF pp7,14-15, Table 2 and Figure 1
Rosales 2026 [D00808]	Early neurological deterioration; 24 hours	OR 1.03 (0.96–1.10)	reported as adjusted for age, sex and admission NIHSS	Source primary/selected secondary estimate; descriptive, not pooled; PDF pp7,14-15, Table 2 and Figure 1
Rosales 2026 [D00808]	In-hospital mortality; as labelled	OR 0.95 (0.38–2.42)	adjusted for age, sex and admission NIHSS	Source primary/selected secondary estimate; descriptive, not pooled; PDF pp7,14-15, Table 2 and Figure 1
Rosales 2026 [D00808]	mRS 0–1; 90 days	OR 6.80 (1.35–34.20)	adjusted for age, sex and admission NIHSS	Source primary/selected secondary estimate; descriptive, not pooled; PDF pp7,14-15, Table 2 and Figure 1

Study / report	Outcome (C vs D)	Measure and estimate (95% CI)	Selected source model	Review role
Romoli, 2026 [D00067]	Early neurological deterioration (NIHSS increase ≥4 within 48 hours); Within 48 hours	OR 0.82 (0.69–0.98)	estimand 1 IPTW	Source primary/selected secondary estimate; descriptive, not pooled; PDF pp4-5,8-9 / article e214528(4-5,8-9), Table 2, Results text and Figure 3
Romoli, 2026 [D00067]	Ordinal modified Rankin Scale at 90 days; 90 days	common OR 1.31 (1.17–1.47)	estimand 1, stabilized IPTW treatment-policy total effect	Source primary/selected secondary estimate; descriptive, not pooled; PDF pp4-5,8-9 / article e214528(4-5,8-9), Table 2, Results text and Figure 3
Romoli, 2026 [D00067]	Successful reperfusion, TICI 2b-3; End of procedure	OR 3.09 (2.53–3.77)	estimand 1 IPTW	Source primary/selected secondary estimate; descriptive, not pooled; PDF pp4-5,8-9 / article e214528(4-5,8-9), Table 2, Results text and Figure 3
Romoli, 2026 [D00067]	mRS 0–1; 90 days	OR 1.27 (1.08–1.50)	estimand 1 IPTW	Source primary/selected secondary estimate; descriptive, not pooled; PDF pp4-5,8-9 / article e214528(4-5,8-9), Table 2, Results text and Figure 3

Study / report	Outcome (C vs D)	Measure and estimate (95% CI)	Selected source model	Review role
Sousa 2025 [D00141]	Any intracranial haemorrhage; 24 hours	OR 2.02 (1.11–3.67)	primary age/NIHSS-adjusted	Source primary/selected secondary estimate; descriptive, not pooled; PDF pp5-6 / printed pp812-813, Tables 2-3
Sousa 2025 [D00141]	Mortality; 90 days	HR 0.63 (0.23–1.70)	primary age/NIHSS-adjusted	Source primary/selected secondary estimate; descriptive, not pooled; reported as an HR from a time-to-event model, not a binary OR; PDF pp5-6 / printed pp812-813, Tables 2-3
Sousa 2025 [D00141]	Recurrent ischemic stroke; 90 days; as labelled	HR 0.57 (0.21–1.56)	Primary model adjusted for age and NIHSS; analytic N=324	Descriptive; PDF p5, Table 2
Sousa 2025 [D00141]	Successful reperfusion, TICI 2b-3; End of procedure	OR 2.62 (1.52–4.50)	primary age/NIHSS-adjusted	Source primary/selected secondary estimate; descriptive, not pooled; PDF pp5-6 / printed pp812-813, Tables 2-3
Sousa 2025 [D00141]	Time-to-event symptomatic intracranial haemorrhage through 90 days; 90 days	HR 1.69 (0.01–240.60)	primary age/NIHSS-adjusted accelerated failure-time survival model	Source primary/selected secondary estimate; descriptive, not pooled; reported as an HR from a time-to-event model, not a binary OR; PDF pp5-6 / printed pp812-813, Tables 2-3

Study / report	Outcome (C vs D)	Measure and estimate (95% CI)	Selected source model	Review role
Sousa 2025 [D00141]	mRS 0–1; 90 days	OR 1.18 (0.84–1.64)	primary age/NIHSS-adjusted	Source primary/selected secondary estimate; descriptive, not pooled; PDF pp5-6 / printed pp812-813, Tables 2-3

One source primary or review-selected model per endpoint is used; Farooqui propensity-matched sensitivity estimates are reported alongside distinct main models. Repeated imputations, IPTW alternatives and duplicate results are not presented as independent evidence. SMD, standardized mean difference.

HR, common OR, binary OR and standardized mean difference retain the source effect-measure labels. The IRIS NIHSS and procedure-duration effects are standardized mean differences, not differences in raw NIHSS units or minutes. No means are inferred from medians.

STOP-CAD recurrent ischemic stroke is reported using the primary age/NIHSS-adjusted Table 2 HR of 0.57 (0.21–1.56). The source labels late sICH estimates as HR and notes Cox or accelerated-failure-time models; that label is retained without reinterpreting it as a binary OR.

The ESCAPE-NA1 estimate of 1.8 (0.7–4.7) is not pooled or interpreted as ordinal mRS because its endpoint is not identified in the report or supplement.

CERES estimand 1 is retained. Its Table 2 ordinal CI upper bound is 1.47, whereas Results text gives 1.46; the Table 2 interval is reported. Estimand-2 mediator coefficients are not misrepresented as C-versus-D effects on bleeding or reperfusion.

The ETIS source outcomes provide descriptive detail and do not enter the adjusted synthesis. The reported effect estimates and confidence intervals are from the identified sources.

Table S8e. Available ordinal mRS distributions and summaries

Study / report	Time / population	C distribution or summary	Comparator distribution or summary	Precision / interpretation
Farooqui 2023 [D00285]	90 days; unmatched outcome-evaluable patients	mRS 0–6 counts: 46, 73, 58, 47, 43, 6, 52; N=325	D: 14, 29, 29, 36, 33, 11, 44; N=196	Main Figure 2 (PDF p8); directly printed category counts, not read from bar lengths. PSM distribution/N NR; source adjusted ordinal effect in S8d.
Beltrán-Guaquetá 2026 [D00030]	Variable follow-up, not 90 days	mRS 0–6 counts: 3, 2, 4, 1, 6, 1, 4; N=21	D: 5, 3, 0, 2, 9, 3, 23; N=45	Source-reported mRS category counts at variable follow-up; not a fixed 90-day assessment.
IRIS 2026 [D00081]	90 days	Median 2 (IQR 1–4); N=112	D: median 3 (IQR 2–5); N=216	Main Table 2 (PDF p8); 1 C patient missing. Adjusted common OR in S8d.
Romoli, 2026 [D00067]	90 days; source unweighted summary	Median 3 (IQR 1–4); enrolled N=2522	D: median 4 (IQR 2–5); enrolled N=1531	Main Table 2 (PDF p8). Figure 2 bars are IPTW-weighted proportions, not raw integer event counts.

Study / report	Time / population	C distribution or summary	Comparator distribution or summary	Precision / interpretation
Rosales 2026 [D00808]	90 days	Median 2 (IQR 0–6); ordinal arm N NR	D: median 5 (IQR 2–6); ordinal arm N NR	Main Table 2 (PDF p14) states ordinal total N=85 but binary mRS denominators sum to 87; The binary denominators of 62/25 are not used as ordinal sample sizes. Unadjusted ordinal OR in S8d.

Ordinal category counts, medians and weighted distributions are different data types. No counts are inferred from weighted bars or summary medians. The SECURIS unweighted, complete-category reconstruction and its rounding discrepancy are explicitly documented in Table S8h. No ordinal analysis is fitted.

Table S8f. Available continuous outcome and follow-up summaries

Study / report	Outcome / unit	C summary	Comparator summary	Precision / source
Kalmar 2021 [D00399]	Puncture to revascularization time; minutes	Median 49 (IQR 33–65); N=72	B: Median 43 (IQR 30–60); N=29	Source CI NR; 6; Table 2
IRIS 2026 [D00081]	Procedure duration; minutes	Median 75 (IQR 55–109); N=109	D: Median 59 (IQR 37–95); N=212	Source CI NR; 33; Table 2 (adjusted standardized mean difference in S8d)
Beltrán-Guaquetá 2026 [D00030]	Follow-up duration; days	Mean 151.87 (SD 111); N=21	D: Mean 206.73 (SD 156); N=45	Source CI NR; 6; Table 1
IRIS 2026 [D00081]	NIHSS, 5–7 days or discharge if earlier	Median 5 (IQR 1–12); N=112	D: median 5 (IQR 1–14); N=207	Main Table 2, PDF p8; explicit 1/9 missing; adjusted standardized mean difference in S8d
ESCAPE-NA1 2022 [D00403]	NIHSS, 24 hours	Median 5 (IQR 2–12); arm outcome N NR	D: median 5 (IQR 2–13); arm outcome N NR	Supplement Table II, PDF p3; CI NR; no median-to-mean conversion
Özdemir 2025 [D00124]	Procedure duration; minutes	Median 60 (range 25–105); source C group N=35	A: median 45 (range 30–90); source A group N=19	Main Table 1, PDF p4; range is not IQR; CI NR
Farooqui 2023 [D00285]	Arterial puncture to reperfusion; minutes	Median 58 (IQR 38–88); arm N NR	D: median 54 (IQR 33–87); arm N NR	Main Table 1 continued, PDF p7; outcome total N=599; no split by arm reported; CI NR

Medians (IQR) are not converted into means/SDs. No unreported CI or mean difference is estimated. Follow-up duration is reported to clarify endpoint timing, not as an efficacy outcome.

Table S8g. Source-reported single-arm cervical-device outcomes

Study / report	Outcome	Available source result	Denominator qualification / source
Sousa 2025 [D00141]	Stent thrombosis	21/150 (14.0%) of stented cohort	Follow-up carotid imaging available in 127/150; source percentage uses 150. The published proportion is 21/150, not 21/127. PDF p6 / printed p813.

Study / report	Outcome	Available source result	Denominator qualification / source
Sousa 2025 [D00141]	Stent migration	1/150 (0.7%) of stented cohort	Noncomparative device event; no nonstented counterpart or comparative effect. PDF p6.
Özdemir 2025 [D00124]	Stent thrombosis	0 reported among 35 stented patients	Main Results PDF p3; noncomparative report, not a comparative safety-equivalence claim.
Madden 2023 [D00989]	Stent occlusion among patients with follow-up imaging	12/21 (57%)	ESOC 2023 abstract 567; PDF p225 / printed p227. Imaging N=21 of 30 stented patients. Mortality and sICH only described qualitatively, without usable counts or CIs.

Single-arm device events are not pooled with C-versus-D comparisons and cannot establish comparative safety or patency. Follow-up imaging completeness is retained.

Table S8h. Additional source-level results and synthesis roles

Report / family	Comparison and sample	Available outcome results	Synthesis role / source qualification	Source location
L37 (Wahood 2022)	C vs B; published weighted group estimates 6,632 vs 3,357	Mortality OR 0.58, p<0.001; non-routine discharge OR 0.78, p=0.063. CIs and event counts not reported in the abstract.	No 90-day mRS 0-2 or sICH; administrative weighted totals and recruitment periods inconsistent within source.	JVIR 2022; 33(6 Suppl):S107, Abstract 233, Results; doi:10.1016/j.jvir.2022.03.314.
L38 (Garg 2021, P553)	Combined CAS/angioplasty vs no cervical revascularization; 1,063 propensity-matched pairs	Index-admission haemorrhagic transformation OR 1.34 (95% CI 1.07–1.69). No numerical cervical-strategy mortality effect in the abstract.	Combined B+C is not a defined A/B/C/D node; no co-primary outcome; same-procedure timing and true tandem lesions in all controls not established in abstract.	Stroke 2021; 52(Suppl 1), Abstract P553, Methods/Results; doi:10.1161/str.52.suppl_1.P553; publisher-deposited abstract.
L44 (Garg 2021)	C=1,391 vs B=651 reported survey-weighted hospitalizations. Readmission subset C=912/B=451; not raw independent-patient counts.	Reported survey-weighted C/B counts: ICH 366/1,391 vs 147/651; in-hospital death 192/1,391 vs 75/651; discharge home 424/1,391 vs 167/651. ICH/death composite: 659/2,042 overall; arm composite counts unavailable. C/B aORs: composite 1.21 (95% CI 0.89–1.65), p=0.213; home 1.49 (1.05–2.12), p=0.025; adjusted for age, sex, income, antiplatelets and IV thrombolysis. Non-elective 30-day readmission: 95/912 vs 32/451; reported HR 1.57 (0.94–2.63), p=0.087 (HR-specific adjustment unspecified). Other reported C/B counts: seizure 36/17, oedema 323/175, MI 44/25, ventilation >24 h 252/135, tracheostomy 33/31 and PEG 159/79. These are weighted estimates, not raw binomial samples.	Discharge home is not 90-day mRS; coded ICH is not sICH. Weighted totals are not raw binomial events. The full publication is selected over L42. The readmission HR is conditional on discharge survival; its adjustment set is unspecified. Supplemental subgroup effects are descriptive, not additional independent network samples.	Table 1 (PDF p3); Figure 1/caption (p4); readmission Methods/Results (pp2–3); supplement Tables S1–S3; doi:10.1016/j.jns.2021.117588.

Report / family	Comparison and sample	Available outcome results	Synthesis role / source qualification	Source location
L44 (Garg 2021), continued	C vs B; same weighted cohort	Source aORs (95% CI): seizure 0.96 (0.33–2.79), p=0.934; cerebral oedema 0.82 (0.59–1.13), p=0.229; MI 0.82 (0.40–1.67), p=0.581.	Adjusted for age, sex, income, antiplatelets and IV thrombolysis. Descriptive inpatient outcomes from the same cohort.	Figure 1 and caption, PDF p4; doi:10.1016/j.jns.2021.117588.
L44 (Garg 2021), continued	C vs B; same weighted cohort	Source aORs (95% CI): ventilation >24 h 0.84 (0.58–1.20), p=0.327; tracheostomy 0.48 (0.23–1.01), p=0.053; craniectomy 0.19 (0.01–2.53), p=0.171; PEG 0.90 (0.59–1.36), p=0.619.	Same Figure 1 adjustment set. Supplement S2–S3 reports overlapping income/tPA strata, not independent evidence.	Figure 1, PDF p4; supplement S2–S3, pp2–4; doi:10.1016/j.jns.2021.117588.
L52 (Paipa Merchan 2018)	A=80, B=22, C=18	TICI 2b-3: A 53/80, B 18/22, C 13/18. Overall tandem mRS 0-2 reported as 41.2%; arm-specific functional independence and sICH counts unavailable. Source percentages for reperfusion do not exactly match printed counts.	Only reperfusion has arm counts. No eligible co-primary result is reported; reperfusion results are presented descriptively only.	ESOC 2018, AS07-089, printed p195; Methods/Results; supplement doi:10.1177/2396987318770127.
L54 (Ramakrishnan 2018)	C=6 vs B=3	TICI 2b-3: C 100% vs B 67%; 90-day mRS ≤1: C 3/6 vs B 0/3; direct discharge home: C 3/6 vs B 0/3. No numerical sICH result.	The function threshold is mRS ≤1, not 0-2. sICH events are not reported, rather than reported as zero.	JNIS 2018; 10(Suppl 2):A128, E-160, Methods/Results; doi:10.1136/neurintsurg-2018-SNIS.236.
L56/L57/L63 (Weill/CHUM family)	C=26 vs non-stenting/deferred intervention=47; A/B not separated	C/non-stenting: mRS 0-2 46%/30% (p=0.16); sICH 8%/4% (p=0.54); intracranial TICI 2b-3 77%/79% (p=0.86); ICA recanalization 92%/9% (p<0.0001). Endpoint-specific denominators and CIs unavailable.	Same 73-patient cohort in three reports; non-stenting comparator not split into A/B. Baseline sample sizes do not establish exact follow-up denominators for reconstructing expanded-network event counts.	L56: ESOC AS07-006, p172; L57: Int J Stroke 2018 abstract p124; L63: Int J Stroke 2017 p39.
L59 (Rodriguez-Villatoro 2018)	B=40 vs C=52	ICA stenosis/occlusion: B 86.5% vs C 21.2%, p<0.001; exact assessed denominators unavailable. No numerical group functional/HT/recurrence estimates. Source methods print mRS ≥2 at three months; this is not reported as mRS 0-2.	No arm co-primary events/effects. Cervical restenosis is distinct from intracranial TICI reperfusion.	Stroke 2018; 49(Suppl 1), WMP40, Methods/Results; doi:10.1161/str.49.suppl_1.WMP40; publisher-deposited abstract.
L65 (Motta 2016)	C=32 vs non-CAS=28; n60 overall, aetiology subgroup analyses	CAS term in atherosclerotic subgroup p=0.004; in cardioembolic subgroup p=0.29. Ordinal regression described CAS as associated with outcome only in atherosclerotic patients, but OR/CI and arm events unavailable.	Non-CAS procedures not separable into A/B; no numerical eligible effect. Regression includes post-treatment TICI and sICH, so it does not establish a total-effect estimate.	Cerebrovasc Dis. 2016; 41(Suppl 1):8, Methods/Results.

Report / family	Comparison and sample	Available outcome results	Synthesis role / source qualification	Source location
Fahed 2016 [F16]	C=37 vs D=33 EVT attempts; three-month analysed C=36/D=31. A/B composition not separable.	C/D: three-month mRS 0–2, 18/36 vs 15/31 (derived unadjusted OR 1.07, 95% CI 0.41–2.79); three-month death, 3/36 vs 6/31 (0.38, 0.09–1.66); TICI 2b–3, 25/37 vs 22/33 (1.04, 0.38–2.83); HT at 24 h, 3/37 vs 2/33 (1.37, 0.21–8.74); HT within 72 h, 4/37 vs 4/33 (0.88, 0.20–3.83). All ORs are calculated from Table 3 using a log-OR Wald CI, not author-reported adjusted effects. No strategy-adjusted OR or sICH result.	Descriptive only; not independently pooled alongside TITAN because partial overlap is plausible. The EVT-start cohort includes aborted procedures and does not isolate completed thrombectomy. No strict A/B/C split or sICH. Mortality, reperfusion and HT remain secondary descriptive outcomes.	Full report, Table 3 (PDF p5 / printed p302); Methods/Results PDF pp2–4 and discussion pp6–7; doi:10.1159/000444068. Overlap: TITAN centre/author list, doi:10.1016/j.jcin.2018.05.036; TITAN dates, doi:10.1136/neurintsurg-2018-014629.
Nolan 2021 [D00445]	C34 vs D21 (A13+B8); Table 3 outcome N not specified	90-day mRS 0–2: OR 0.64 (95% CI 0.19–2.19); symptomatic PH1/PH2 during admission: OR 1.86 (0.34–10.24); TICI 2b–3: OR 0.91 (0.29–2.87). Overall reperfusion 35/60; sICH 8/60. Stent patency 12/22 at median 7 days (IQR 1–70).	Contextual source-reported effects only. Table 3 supplies neither arm event counts nor covariates/adjustment status, so it is incompatible with the raw-count network and cannot be classified as a documented baseline-adjusted model. No A/B-separated outcomes.	Full article Tables 1–3, Methods/Results; PMCID PMC7855424.
Troja 2021 [D01132]	C35 vs non-stent 34; non-stent procedures unclear	At 90 days after discharge: re-occlusion p=0.434; neurological outcome p=0.82. No event counts, numerical effects or CIs; functional threshold unspecified.	Insufficient numerical comparative results; follow-up begins at the source-defined time origin.	Abstract 12852; doi:10.1161/circ.144.suppl_1.12852.
STAR [D01000/D00943]	WP164: C132/A444; matched 129/129. O-068: C132/B112. One report family.	WP164 C/A: 90-day mRS 0–2 37.5%/30.4%; mortality 17.5%/29.6%; sICH 11.1%/15.4%. O-068: adjusted ATEs (95% CIs), function −0.13 (−0.29 to 0.02), mortality 0.05 (−0.06 to 0.15), sICH 0.04 (−0.05 to 0.13). Reperfusion C/B 95%/83.9%.	Outcome-specific denominators missing. ATEs are not ORs; treatment coding is unspecified. WP164 raw reperfusion order contradicts its prose; matched C/A 94.8%/84.4%. Possible shared C arm; no double counting.	Abstract WP164, doi:10.1161/str.54.suppl_1.WP164; O-068, doi:10.1136/jnis-2024-snis.68.
Bajrami 2024 [D00935]	A13/B47/C142 (43 balloon-expandable +99 self-expandable stents)	Three-month mRS 0–2 favoured self-expandable stents ($p<0.05$); sICH group difference $p>0.05$; carotid patency specified but no numerical result.	No arm outcome counts, rates or effect sizes. Stent-subtype p values cannot be combined into a C-node effect.	WFITN abstract 1414; PDF p180, printed p181; doi:10.1177/15910199241285197.
Kaba–Beyazal 2025 [D00893]	C7 vs B13	End-procedure TICI 2b–3 C7/7 vs B13/13. Any haemorrhagic transformation at 24h: C3/7 vs B6/13, uniquely reconstructed from 43%/46% and checked against total 9/20. Discharge mRS 0–2 7/20, mRS3–5 4/20, deaths 9/20 overall.	Secondary descriptive evidence, not 90-day mRS or sICH. Reperfusion counts follow explicit success in all 20 patients. No uncorrected finite reperfusion OR.	Neuroradiology67(Suppl1): S90–S91; original indexed abstract, Embase accession 648927547.
Florez 2025 [D00869]	C26 vs B13	90-day mRS and procedural complications described as assessed; no numerical outcome results in the abstract.	No estimable cervical-strategy effect; no overlap exclusion presumed.	Abstract A205; doi:10.1136/jnis-2025-esmint.171.

Report / family	Comparison and sample	Available outcome results	Synthesis role / source qualification	Source location
Chen 2026 [D00029]	C1015 vs A3371; B541. Reported total 4928 vs displayed sum 4927.	Discharge functional status: adjusted common OR 1.27 (95% CI 1.04–1.55). In-hospital mortality: aOR0.62 (0.47–0.82), C11.2%/A21.6%. Any ICH: aOR 1.27 (0.99–1.64), C37.5%/A34.4%. B/A described only as p>0.05 without effect estimates.	Inpatient/discharge outcomes are not 90-day function,90-day mortality or sICH. The source total discrepancy and potential overlap with shorter NRD reports are noted.	Primary abstract PMID 42062037; doi:10.3174/ajnr.a9169.
TATTOO 2019 [L50]	n172; C41.7%, B29.2%, aspiration 7.7%, A21.4%	Overall three-month mRS≤2 49.7%, mortality 8.1%; sICH7%; TICI 2b–3 77.3%.	No strategy-specific outcome counts or cervical-strategy effect; procedural-order/general-anaesthesia effects are different contrasts.	ESOC2019 e-poster AS07-038, printed p481, Methods/Results.
Sallustio 2023 [D00320]	C122 vs D226 enrolled; D includes 23 angioplasty cases	Three-month mRS 0–2 C54/118 vs D77/211; mortality C14/118 vs D50/211. sICH≤24h C6/116 vs D5/217; TICI 2b–3 C97/122 vs D147/226. Table 4 sICH aOR2.477 (95% CI 0.707–8.684), adjusted for age and onset-to-puncture time.	Descriptive only; not independently pooled alongside the retained TITAN report because of possible partial Careggi overlap. This does not establish full duplication. Procedure-time-adjusted functional models remain contextual; source percentage/model conflicts are retained below.	Table 1 PDF p4/printed 478; Tables 2–4 p5/479; Methods pp 2–3/476–477; centres p7/481; doi:10.1007/s13760-022-02067-z.
Abousleiman 2025 [D01439]	Full cohort B26/C134; eligible tandem subgroup B19/C100	Published function, mortality and sICH outcomes concern all 160 dissection cases, including 41 isolated extracranial ICA cases.	Tandem-only outcomes cannot be isolated. The119 subgroup count is not the outcome denominator for the full-cohort tables.	Full article Methods/Results PDF pp 2–3; Tables 1–2 p4.
Simon 2024 [D00199]	Acute CAS before vs after thrombectomy:31 vs 19 (C vs C)	Three-month mRS 0–2 13/27 vs 9/19; ordinal common OR 0.81 (95% CI 0.29–2.30).	Wrong treatment contrast for A/B/C/D synthesis. The80 screened tandem cases include 30 nonstented patients without reported inter-node outcomes.	Full article selection/flow pp 2–3; Table 2 p5/printed 1369.
SECURIS 2025 [D00087]	C, 344; non-eCAS D, 234; 90-day mRS	Strict mRS 0–2: 154/344 versus 84/234, reconstructed from Figure 2. Mortality: 71/344 versus 50/234.	Descriptive only; possible centre and recruitment-period overlap with the retained ASSIST and IRIS reports prevents independent pooling. A/B composition is unresolved. The figure reconstruction and percentage discrepancy are described below.	Figure 2A, p. 7; mortality total of 121, p. 5.
Wichianrat 2026 [D00803]	Published C20 vs non-stenting 59; full 92 baseline C20/D72 includes 13 delayed treatments	Selected79: mRS 0–2 C13/20 vs 27/59; sICH1/20 vs 3/59; any ICH10/20 vs 12/59; mortality 3/20 vs 6/59. Reconstructed full 92 C/D: mRS 0–2 13/20 vs 38/72; sICH1/20 vs 3/72; mortality 3/20 vs 6/72; reperfusion 19/20 vs 60/72.	Descriptive only; not quantitatively pooled. Seven separately labelled CCA cases cannot be removed by acute strategy and outcome to isolate the eligible cervical-ICA population. Full-cohort numeric reconstruction does not solve this anatomical restriction.	Table 1 pp 6–7/printed 337–338; Table 4 p12/343; Figures 4–6 pp 11,14/342,345.

Report / family	Comparison and sample	Available outcome results	Synthesis role / source qualification	Source location
SECURIS 2025 [D00087]	C versus D; adjusted 90-day function	Ordinal cOR 1.47 (1.20–1.82). Source-defined favourable binary OR 1.43 (1.12–1.82); events, 158/344 versus 86/234.	IPTW includes procedural pass count; these estimates do not enter the adjusted pool. The binary endpoint includes mRS 3 if prestroke mRS was 3; neither estimate represents adjusted strict mRS 0–2.	Methods and Results, pp. 3–4.
SECURIS 2025 [D00087]	One-year mRS: C, 320; D, 181	Ordinal cOR 1.47 (1.16–1.85); source-defined favourable OR 1.35 (1.04–1.76). Strict mRS 0–2: 153/320 versus 73/181.	One-year context; 24 C and 53 D patients lack follow-up. Figure 2 mortality is 77/320 versus 53/181; body percentages differ.	Results, pp. 4–5; Figure 2B, p. 7.
SECURIS 2025 [D00087]	C versus D; mTICI ≥2b	Adjusted OR 4.28 (2.61–7.01). Table 1 counts: 313/342 versus 170/233; total, 483/575.	Six-covariate logistic model; covariates and denominator derivation are listed below. The narrative gives C = 315, whereas Table 1 gives 313. No unqualified raw pooling.	Results, p. 5; Table 1, p. 6; Table 2 footnote d, p. 9.
SECURIS 2025 [D00087]	Relevant HT and sICH at 24–36 h	Relevant HT (sICH or any PH): C, 58/344; D, 29 events with an unclear evaluated denominator. Overall sICH: 51; no arm split.	Relevant HT cannot substitute for sICH. The printed D percentage, 12.5%, disagrees with 29/234; 51 (8.9%) also disagrees with cohort N = 578.	Methods, p. 3; Results, p. 5; Table 2, p. 9.

A, no acute targeted cervical treatment; B, angioplasty alone; C, acute carotid stenting; D, intact source-defined nonstenting management. Report-family grouping does not prove patient-level independence. Results are source-reported unless an arithmetic reconstruction is stated. Results in this table are descriptive and do not contribute additional participants to the pooled analyses.

Enrollment is not substituted for outcome-specific follow-up. Source percentages without uniquely verifiable denominators remain percentages. Absence of a numerical result is not zero events. Discharge function, any ICH, mRS 0–1 and procedural-order comparisons are not relabelled as the co-primary outcomes.

Sallustio: outcome N uses Table 1 explicitly bracketed missing values: mRS/mortality C122−4 and D226−15; sICH C122−6 and D226−9. Table 1 prints C54(36.5%) for mRS 0–2, inconsistent with 54/118. Functional models 1–3 adjust for procedure time: ORs1.162(0.595–2.269),1.196(0.639–2.241),1.374(0.710–2.658); they are not baseline-only total-effect estimates. Tables 2–3 label sICH OR 1.091(0.307–3.739) as unadjusted; it is not reconciled with the Table 1 counts or substituted for Table 4 adjusted OR.

Sallustio/TITAN: both identify Careggi University Hospital, Florence (Mangiafico); IRETAS centres are listed on PDF p7 and TITAN Methods/affiliations on PDF p2. Their recruitment periods overlap. TITAN is retained in the pooled analyses, and IRETAS is presented descriptively without adding potentially overlapping participants. This is possible partial overlap, not proof that all 348 IRETAS patients are duplicated.

Chen 2026 [D00029] source-adjusted discharge/in-hospital effects retain their original endpoint and effect-measure labels. Table S5 preserves the original total 4928 versus displayed group sum 4927. Companion reports L41/L42, L56/L57/L63 and STAR are not counted as independent replications.

Wichianrat reconstruction: Figure 4 mRS 0–6 counts are C[4,6,3,1,3,0,3] and non-stent[16,7,4,9,7,10,6]. Figure 6 gives delayed-treatment favourable outcome 11/13; thus D favourable 27+11=38 of 59+13=72. Figure 5 prints sICH C1/20 and non-stent 3/59; the delayed group has no sICH bar, corroborated by total 4 minus 1 minus 3=0. Whole-cohort mortality 9 and reperfusion 79 minus C3 and C19 yield D6 and D60. These are cross-table aggregate reconstructions, not explicitly published 92-person C/D tables. Seven CCA cases include 2 favourable and 5 poor outcomes but lack acute-strategy cross-tabulation; they cannot be subtracted by treatment arm.

Fahed: the single Rothschild treating centre and recruitment period (November 2011–August 2014) intersect TITAN (January 2012–September 2016). The source includes ten procedures stopped during/after initial angiography and describes starting EVT, not completed thrombectomy alone. Fahed is presented descriptively; TITAN remains the pooled report. Overlapping patient counts are not inferred.

Garg: survey-weighted national hospitalization estimates are descriptive, not raw binomial inputs. Main Figure 1 adjusts for age, sex, income, antiplatelets and IV thrombolysis. Supplementary Tables S2 and S3 report overlapping income and thrombolysis strata, not additional independent cohorts. Figure 3 is captioned 90 days, but its axis and Methods/Results specify 30 days; 127/1,363 readmissions is 9.3%, despite an approximately 12% statement in Discussion.

SECURIS abbreviations: eCAS, emergent carotid artery stenting; D, unresolved non-eCAS management; mRS, modified Rankin Scale; HT, hemorrhagic transformation; PH, parenchymal hemorrhage; IPTW, inverse probability of treatment weighting; cOR, common odds ratio. Intervals are 95% confidence intervals.

The result-level grades follow the seven ROBINS-I (2016) domains: S, serious; L, low; M, moderate; NI, no information. S* denotes at least serious overall risk; insufficient information in other domains could support a higher overall risk. D4 and D7 have insufficient information. The 98 exclusions for missing critical follow-up are assessed under D5, without also counting them under D2.

Functional models: the propensity score includes prestroke mRS, age, atrial fibrillation, baseline NIHSS, ASPECTS, time from last seen well to groin puncture, prior intravenous thrombolysis, and number of passes. IPTW-adjusted ordinal logistic regression evaluates 90-day and one-year mRS shifts. The 90-day favourable binary model also uses IPTW; the one-year binary result is reported as adjusted. Number of passes is a procedural variable, so the IPTW estimates are not baseline-only total-effect estimates and are retained outside the adjusted pool. Exact model-specific sample sizes are not given. Source: Methods, pp. 3–4; Results, pp. 4–5.

Reperfusion, mortality and relevant-HT models: multivariable logistic regression includes number of passes, prior intravenous thrombolysis, time from last seen well to groin puncture, age, ASPECTS, and baseline NIHSS. The main Results give the adjusted reperfusion effect but not numerical adjusted mortality or relevant-HT effects. Source: pp. 4–5.

Figure 2A reconstruction: C mRS 0–6 counts are 39, 55, 60, 65, 28, 26 and 71. D mRS 1–6 counts are 19, 45, 38, 36, 26 and 50. These categories imply D mRS 0 = 20; however, 20/234 normally rounds to 8.5%, whereas the figure prints 8.6%. Strict D mRS 0–2 is the complement of mRS 3–6: 234 − 38 − 36 − 26 − 50 = 84. Counts are reconstructed, not directly tabulated. Source: Figure 2A, p. 7.

Reperfusion denominators are recoverable, not assumed from enrolment. Table 2 footnote d reports 342 assessed eCAS patients; Results report 575 assessed patients overall, giving D = 575 − 342 = 233. Table 1 values 313 (91.5%) and 170 (73.0%) independently permit only these respective denominators within the enrolled arm sizes. The narrative gives C = 315, and Table 2 prints 362 successful cases in a no-HT subgroup of 286; these source errors remain unresolved. Source: pp. 5–6 and 9.

The source does not report an observed A/B or deferred-treatment split within non-eCAS. Its discussion of deferred management does not establish how many patients received it.

ASSIST registration NCT03845491 lists Vall de Hebron, and SWIFT DIRECT registration NCT03192332 lists Vall d'Hebron and Germans Trias i Pujol. Their recruitment overlaps SECURIS in 2017–2023. This establishes shared centres and periods, not an exact duplicate-patient count. ASSIST and IRIS remain in the pooled analyses; SECURIS is presented descriptively. CERES-TANDEM remains separate.

ESCAPE-NA1 registration NCT02930018 lists no Spanish sites, supporting geographical independence from SECURIS.

At one year, Figure 2 gives mortality of 24.1% versus 29.3% (77/320 versus 53/181), whereas Results give 24.0% versus 29.9%. These discrepancies do not affect the 90-day functional reconstruction.

D7 is NI for all four results. The article describes prespecified outcomes, covariates and subgroups, but no dated analysis plan was verified. The available report does not establish whether results were selected from alternative measurements or analyses. Missing numerical secondary estimates and internal source discrepancies do not independently establish selective reporting. Source: Methods, pp. 3–4; Results, pp. 4–5.

Table S9. Local direct–indirect evidence separation in the strict network

Outcome	Nodes	Direct OR (95% CI)	Indirect OR (95% CI)	Direct/indirect ratio of ORs (95% CI)	SIDE P
mRS 0–2 at 90 days	B vs A	0.76 (0.31–1.86)	0.80 (0.36–1.77)	0.96 (0.29–3.18)	0.944
mRS 0–2 at 90 days	C vs A	1.33 (0.77–2.29)	1.69 (0.19–14.61)	0.79 (0.09–7.29)	0.833
mRS 0–2 at 90 days	C vs B	1.73 (1.22–2.46)	1.44 (0.06–33.94)	1.21 (0.05–29.03)	0.908
sICH	B vs A	0.27 (0.03–2.44)	1.94 (0.72–5.21)	0.14 (0.01–1.55)	0.110
sICH	C vs A	1.45 (0.69–3.09)	7.75e-05 (4.21e-10–14.25)	1.88e+04 (0.10–3.53e+09)	0.112
sICH	C vs B	1.00 (0.60–1.67)	NE	NE	NE

SIDE: separating indirect from direct evidence. Direct and indirect estimates were obtained from the random-effects network model, with comparison orientations harmonized.

NE: not estimable. For C versus B on sICH, the direct estimate is available but the indirect estimate, direct/indirect ratio and SIDE P value are not estimable.

SIDE direct effects use the fitted network framework and are not the separately reported Hartung–Knapp pairwise summaries. Non-significant inconsistency tests do not establish consistency; sparse indirect paths yield very wide intervals. Scientific notation preserves very small positive bounds rather than rounding them to zero.
